# p38γ and δ promote heart hypertrophy by targeting the mTOR-inhibitory protein DEPTOR for degradation

**DOI:** 10.1038/ncomms10477

**Published:** 2016-01-22

**Authors:** Bárbara González-Terán, Juan Antonio López, Elena Rodríguez, Luis Leiva, Sara Martínez-Martínez, Juan Antonio Bernal, Luis Jesús Jiménez-Borreguero, Juan Miguel Redondo, Jesús Vazquez, Guadalupe Sabio

**Affiliations:** 1 Fundación Centro Nacional de Investigaciones Cardiovasculares Carlos III, CNIC, 28029 Madrid, Spain; 2 Hospital de La Princesa, 28006 Madrid, Spain

## Abstract

Disrupted organ growth leads to disease development. Hypertrophy underlies postnatal heart growth and is triggered after stress, but the molecular mechanisms involved in these processes are largely unknown. Here we show that cardiac activation of p38γ and p38δ increases during postnatal development and by hypertrophy-inducing stimuli. p38γ/δ promote cardiac hypertrophy by phosphorylating the mTORC1 and mTORC2 inhibitor DEPTOR, which leads to its degradation and mTOR activation. Hearts from mice lacking one or both kinases are below normal size, have high levels of DEPTOR, low activity of the mTOR pathway and reduced protein synthesis. The phenotype of *p38γ/δ*^**−/−**^ mice is reverted by overactivation of mTOR with amino acids, shRNA-mediated knockdown of *Deptor*, or cardiomyocyte overexpression of active p38γ and p38δ. Moreover, in WT mice, heart weight is reduced by cardiac overexpression of DEPTOR. Our results demonstrate that p38γ/δ control heart growth by modulating mTOR pathway through DEPTOR phosphorylation and subsequent degradation.

## Abstract (web summary)

mTOR signalling pathway is a critical regulator of cardiac hypertrophy. Here the authors show that two kinases, p38γ and p38δ, control heart growth by promoting mTOR activity via phosphorylation and consequent proteasome degradation of mTOR inhibitor DEPTOR, extending our knowledge of cardiac hypertrophy regulation.

## Graphical abstract

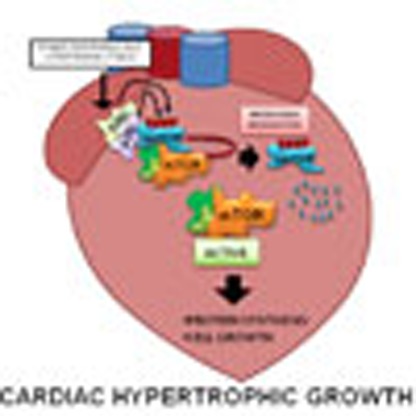

Cardiac growth is tightly regulated to ensure that the heart reaches its appropriate size. Cardiomyocytes rapidly proliferate during fetal life, but soon after birth differentiated cardiomyocytes enter a postmitotic state and the ability to proliferate is lost. Postnatal cardiac growth is therefore mainly achieved through increases in cell size (physiological hypertrophy) associated with increased protein synthesis^1^ together with the expansion of non-myocyte populations^2^. Hypertrophic growth is also the adaptive response of cardiomyocytes to stress stimuli, including cardiac pressure or volume overload, cytoskeletal abnormalities and intrinsic contractility defects. Although cardiomyocyte hypertrophy initially enhances cardiac output in stress situations, prolonged hypertrophy, known as pathological hypertrophy, is associated with an increased risk of morbidity and mortality due to cardiovascular diseases such as diastolic and systolic heart failure and arrhythmia^3^. Understanding the mechanism of cardiac growth thus has important clinical implications. The key unanswered questions are how cells perceive extracellular hypertrophic stimuli and convert them into intracellular signals, and how these signals lead to cardiac hypertrophy^4^.

Several studies identified mammalian TOR (mTOR) as a key regulator of cardiac hypertrophy; for example, the mTOR inhibitor rapamycin prevents heart-weight gain in an overload model of hypertrophy^5^ and blocks cardiomyocyte size increases induced by AngII^6^ and phenylephrine^7^, likely by inhibiting protein synthesis^7^. mTOR is a conserved serine/threonine kinase with a key regulatory function in cardiovascular physiology and pathology^8^. mTOR integrates signals from growth factors, nutrients and stresses to regulate multiple processes, including translation, cell cycle progression, autophagy and cell survival^9^. mTOR function is regulated by the formation of two multi-protein complexes: mTOR complex 1 (mTORC1) and mTOR complex 2 (mTORC2). mTORC1 is composed of the mTOR catalytic subunit and five associated proteins: Raptor, PRAS40, mLST8/GβL, DEPTOR and Tti1/Tel2. In mTORC2, the mTOR catalytic subunit is associated with six proteins: mLST8/GβL, DEPTOR, Tti1/Tel2 (in common with mTORC1) and Rictor, mSin1 and Protor^10^. The activities of the mTORC1 and mTORC2 pathways are regulated by the common inhibitory component DEPTOR^11^.

Stress-inducing stimuli in the heart activate several mitogen-activated protein kinases (MAPK), which are known to participate in hypertrophy, remodelling, contractility and heart failure^12^,^13^. MAPKs are key regulators of cell stress responses, including cell proliferation, differentiation and apoptosis^14^,^15^. The MAPK family consists of the extracellular signal-regulated protein kinases (ERK), the c-Jun NH2-terminal protein kinases (JNK) and the p38 MAP kinases^16^. Several studies have proposed that ERK cascade is involved in the hypertrophic response^17^. The p38 MAPKs are activated in response to proinflammatory cytokines, hormones and stress signals. Four p38 MAPK isoforms have been identified, and can be grouped into two subsets based on sequence homology, substrate specificity and sensitivity to chemical inhibitors: p38α and p38β, and p38γ and p38δ (ref. 15). MKK3 and MKK6, the kinases acting upstream of p38 MAPKs, appear to play a role in cardiac hypertrophy^18^, and susceptibility to cardiac hypertrophy is promoted by cardiac-specific overexpression of dominant-negative p38α (ref. 19). However, findings in mice lacking p38α in cardiomyocytes indicate that this effect is p38α independent^20^, whereas heart development requires endogenous p38β (ref. 21). Although the p38γ isoform is highly abundant in the heart^22^ and p38δ is also expressed in cardiomyocytes^23^, little is known about the role of these isoforms in cardiac hypertrophy^24^.

Here, we report that p38γ and the closely related kinase p38δ are activated by pathological and physiological hypertrophic stimuli and are required for cardiac physiological and pathological hypertrophy. Using p38γ/δ null mice, we show that p38γ and p38δ interact with and phosphorylate the inhibitory mTOR complex component DEPTOR, inducing its degradation and thus promoting cell growth.

## Results

### p38γ and p38δ modulate postnatal cardiac hypertrophic growth

**Figure 1:**
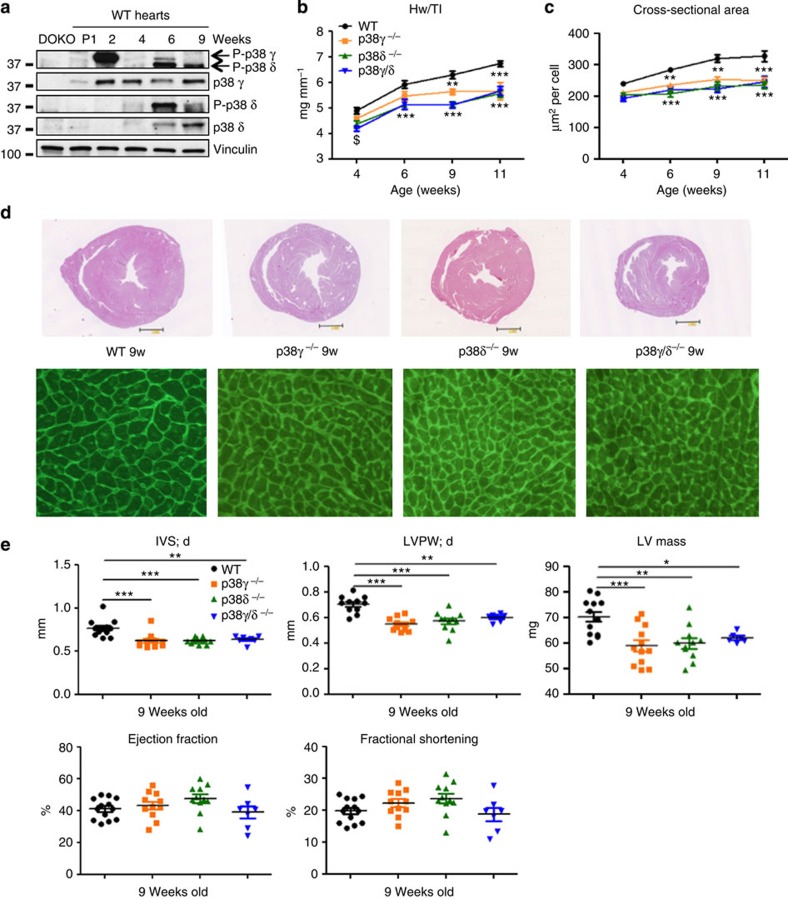
p38γ and p38δ modulate postnatal cardiac hypertrophic growth. (**a**) Immunoblot analysis of p38γ and p38δ activation and expression in heart extracts prepared from wild-type (WT) mice at different ages. (**b**,**c**) WT, p38γ^−/−^, p38δ^−/−^ and p38γ/δ^−/−^ mice were killed at 4, 6, 9 and 11 weeks. (**b**) Heart-weight-to-tibia-length ratio. (**c**) Cardiomyocyte cross-sectional area quantified in wheatgerm agglutinin (WGA)-stained hearts. Data are means±s.e.m. (*n*=5–13). **P<0.01; ***P<0.001 (two-way analysis of variance (ANOVA) coupled to Bonferroni post tests). (**d**) Top: representative haematoxylin and eosin staining of transverse heart sections from 9-week-old WT, p38γ^−/−^, p38δ^−/−^ and p38γ/δ^−/−^ mice. Bottom: Representative staining with FITC-WGA (green) in hearts from 9-week-old WT, p38γ^−/−^, p38δ^−/−^ and p38γ/δ^−/−^ mice. (**e**) Echocardiography results for 9-week-old WT, p38γ^−/−^, p38δ^−/−^ and p38γ/δ^−/−^ mice. IVS;d (inter-ventricular septum in diastole); LVPW;d (left ventricle posterior wall in diastole); LV (left ventricle). Data are means±s.e.m. (*n*=5–13). *P<0.05; **P<0.01; ***P<0.001 (one-way ANOVA coupled to Bonferroni post tests).

Stress stimuli in the heart activate several MAPKs, which have been implicated in mechanisms of cardiomyocyte hypertrophy and survival^13^. p38γ is abundant in heart tissue, and the closely related isoform p38δ is also well expressed^23^. Stress-induced hypertrophy appears to modulate the levels of both kinases^23^, but their function and activation in the heart remain unsolved. Analysis of wild-type (WT) mice showed that expression of both kinases increases during postnatal growth and that they become activated, p38γ activity peaking on postnatal week 2 coinciding with the transition to hypertrophic growth^25^, and p38δ activity peaking on postnatal week 6 (Fig. 1a). To assess the role of p38γ and p38δ in cardiac growth, we used knockout mice lacking p38γ, p38δ or both kinases, confirming the deletion of these kinases in the heart by western blot (Supplementary Fig. 1a). Ventricular mass between postnatal weeks 4 and 11 was monitored relative to tibia length in WT mice and mice lacking one or both of the kinases. Ventricular mass was notably lower in p38γ^−/−^, p38δ^−/−^ and p38γ/δ^−/−^ mice (Fig. 1b). Lower heart mass in the knockout animals correlated with the presence of cardiomyocytes of below-normal size, determined from cell cross-sectional area on histological sections, suggesting a defect in postnatal hypertrophic growth (Fig. 1c,d). Similar analysis conducted at postnatal day 1 showed no differences between genotypes in heart or body size (Supplementary Fig. 1b,c), indicating that p38γ/δ kinases modulate hypertrophic heart growth in the postnatal period, and not in the fetus.

Echocardiography of 9-week-old mice revealed a thinner diastolic inter-ventricular septum (IVS) and telediastolic left ventricular posterior wall (LVPW;d) in p38γ^−/−^, p38δ^−/−^ and p38γ/δ^−/−^ mice than in age-matched WT mice, and this correlated with a below-normal corrected left ventricular mass (Fig. 1e). However, the knockout models had normal systolic function, with no observed differences from WT in ejection fraction or fractional shortening (Fig. 1e). Moreover, haematoxylin and eosin and Picrosirius red staining revealed no differences in fibrosis or cardiac morphology (Supplementary Fig. 1d,e).

### p38γ and p38δ control pathological hypertrophy

**Figure 2:**
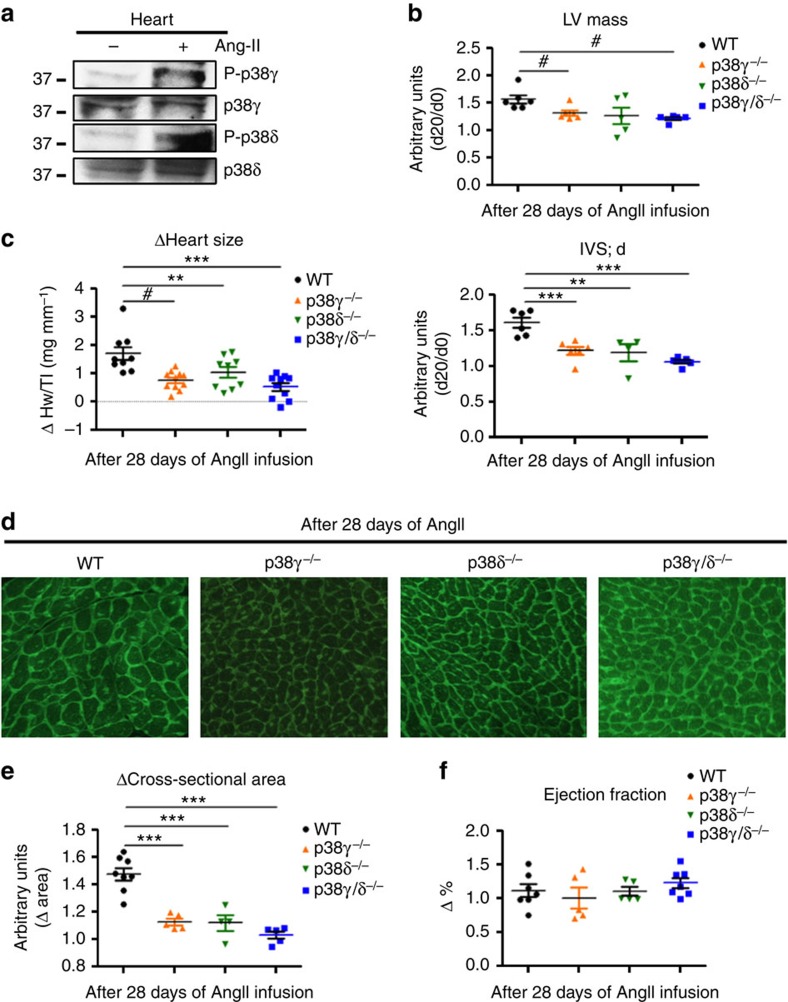
p38γ and p38δ control angiotensin II-induced hypertrophy. WT, p38γ^−/−^, p38δ^−/−^ and p38γ/δ^−/−^ mice were treated for 28 days with angiotensin II (AngII) (1 μg kg per minute) or saline, delivered by subcutaneously implanted osmotic minipumps. (**a**) Immunoprecipitation analysis of the phosphorylation and protein levels of p38γ and δ isoforms in heart extracts prepared from WT mice treated with AngII or saline. (**b**) Echocardiography results from AngII-treated WT, p38γ^−/−^, p38δ^−/−^ and p38γ/δ^−/−^ mice shown as the change relative to saline-treated controls. (**c**) Heart-weight-to-tibia-length ratios for WT, p38γ^−/−^, p38δ^−/−^ and p38γ/δ^−/−^ after 28 days of Ang II treatment. (**d**,**e**) Top: representative FITC-WGA staining (green) in hearts from 9-week-old WT, p38γ^−/−^, p38δ^−/−^ and p38γ/δ^−/−^ mice after AngII treatment. (**e**) Cardiomyocyte cross-sectional area quantified in WGA-stained hearts. (**f**) Echocardiography evaluation of systolic cardiac function increment after AngII treatment. IVS;d (inter-ventricular septum in diastole); LV (left ventricle). Data are means±s.e.m. (*n*=6–12). **P<0.01; ***P<0.001 (one-way analysis of variance coupled to Bonferroni post-tests); ^#^*P*<0.05 (*t*-test between indicated groups).

The involvement of p38γ and p38δ in physiological postnatal hypertrophic heart growth prompted us to examine whether these kinases are activated by angiotensin (Ang) II, a stress stimulus that induces hypertrophy in the adult heart^26^,^27^. AngII induced phosphorylation and activation of p38γ and p38δ kinases in the heart (Fig. 2a). Osmotic minipump administration of AngII over 28 days increased systolic arterial pressure in WT and all the three knockout models (Supplementary Fig. 2a), but only WT mice showed above-baseline increases in LV mass and IVS thickness (Fig. 2b and Supplementary Fig. 2b,c). In WT mice, heart mass (measured as the ventricular-weight-to-tibia-length ratio) increased almost 2-fold after 28 days of AngII infusion, whereas p38γ/δ^−/−^ mice showed no hypertrophic growth (Fig. 2c and Supplementary Fig. 2d). Analysis of heart sections showed that AngII increased cardiomyocyte cross-sectional area in WT mice by around 50%; in contrast, cardiomyocyte cross-sectional area in AngII-treated p38γ/δ^−/−^, p38γ^−/−^ and p38δ^−/−^ mice did not differ from that observed in saline-treated mice (Fig. 2d,e and Supplementary Fig. 2e). AngII treatment did not affect ejection fraction in WT or single or double KO mice, indicating preserved systolic ventricular function (Fig. 2f and Supplementary Fig. 2f), and the extent of fibrosis was similar in all genotypes (Supplementary Fig. 2g).

### Impaired mTOR activation in hearts of p38γ or p38δ null mice

**Figure 3:**
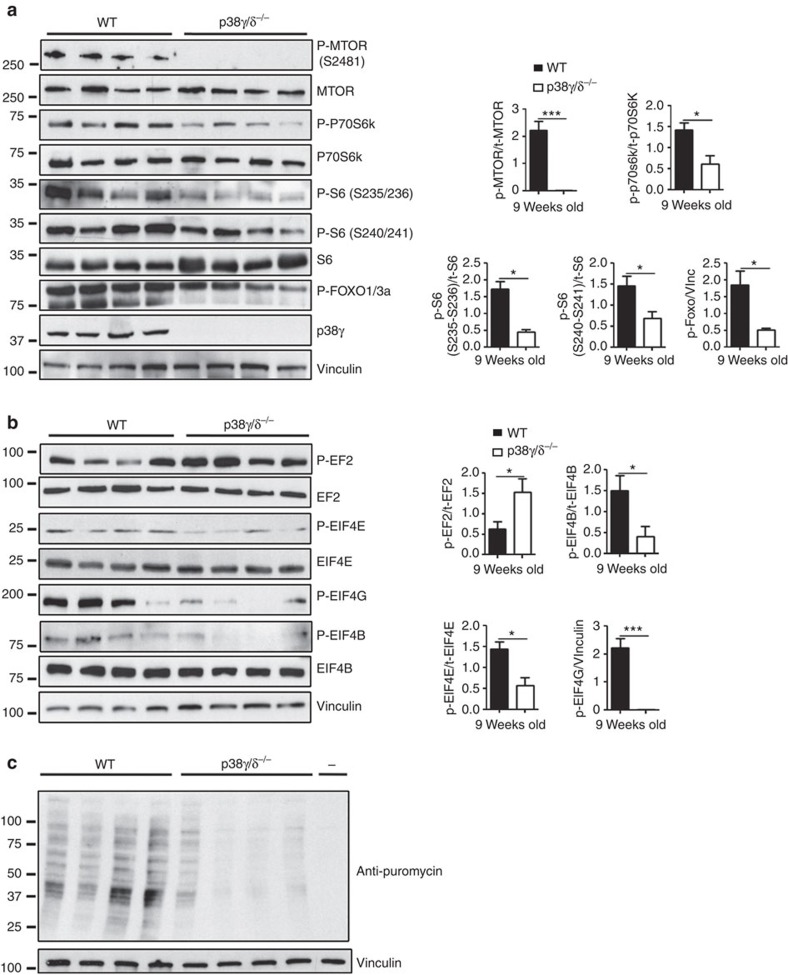
Hearts from p38γ/δ^−/−^ mice show reduced mTOR activation. (**a**,**b**) Immunoblot analysis of mTOR signalling pathway activity (**a**) and the activation status of translation factors (**b**) in heart lysates of 9-week-old WT and p38γ/δ^−/−^ mice. Bar charts show quantification of total protein or vinculin-normalized band intensities (*n*=4). Data are means±s.e.m. *P<0.05; ***P<0.001 (*t*-test). (**c**) *In vivo* measurement of protein synthesis. Mice were injected intraperitoneally with 0.040 μmol g^−1^ puromycin dissolved in 100 μl PBS. Exactly 30 min after injection, tissues were extracted and frozen in liquid N_2_ for immunoblot analysis with anti-puromycin antibody (*n*=4).

The size of mammalian cells is controlled by the mTOR pathway^28^. To assess whether mTOR signalling is altered in mice lacking p38γ and p38δ (Supplementary Fig. 3a), we analysed heart protein extracts by immunoblot. We found that cardiac activation of mTORC1 and mTORC2 was impaired in p38γ/δ^−/−^, p38γ^−/−^ and p38δ^−/−^ mice, as assessed by the phosphorylation of the mTOR targets p70S6K, S6 and FOXO1/3a and by mTOR autophosphorylation on Ser 2481 (Fig. 3a, Supplementary Fig. 3b,c); in contrast, no changes were detected in the expression or activation of p38α (Supplementary Fig. 3d). A major function of mTORC1 is the promotion of protein synthesis^8^, an essential driver of hypertrophic cardiomyocyte growth. Immunoblot analysis of translation initiation/elongation factors in the hearts of p38γ/δ^−/−^ mice revealed weak phosphorylation of EIF4E, EIF4G and EIF4B and strong phosphorylation of EF2 (Fig. 3b), indicating that cardiac protein synthesis is impaired in these mice. To confirm this finding, we injected puromycin into WT and p38γ/δ^−/−^ mice 30 min before heart extraction and measured its incorporation into newly synthesized proteins^29^. p38γ/δ^−/−^ hearts contained fewer puromycin-labelled peptides than WT hearts, indicating a lower rate of protein synthesis (Fig. 3c). The p38γ/δ pathway thus appears to support growth induction in the heart by promoting the activation of mTOR-regulated protein synthesis.

### p38γ and p38δ interact with mTOR through DEPTOR

**Figure 4:**
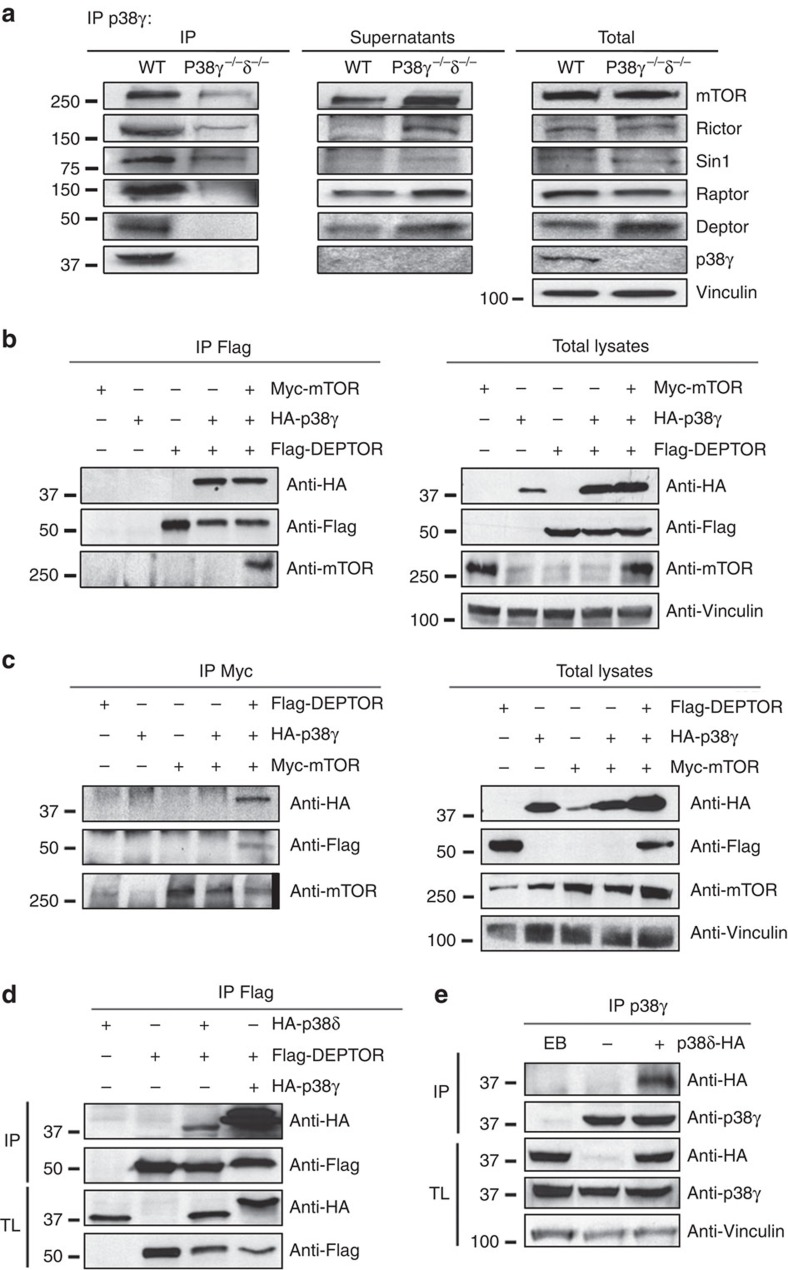
p38γ and p38δ interact with mTOR through DEPTOR. (**a**) Endogenous mTOR, Raptor, Rictor, Sin-1 and DEPTOR co-immunoprecipitate with endogenous p38γ. We immunoprecipitated p38γ from WT and p38γ/δ^−/−^ MEF lysates using specific antibodies; immunoprecipitates (IP), supernatants and total lysates were analysed by SDS–PAGE using the antibodies indicated. (**b**,**c**) p38γ interacts with mTOR through DEPTOR. HEK-293 cells were transfected with HA-p38γ, Flag-DEPTOR or Myc-mTOR or a combination of these and immunoprecipitated with the indicated antibodies targeting the c-myc epitope (**b**) or Flag (**c**). Immunoblots were probed with the indicated antibodies. (**d**) Co-immunoprecipitation of p38γ and p38δ with DEPTOR in HEK-293 cells. HA-p38γ or HA-p38δ expression vectors were co-expressed with Flag-DEPTOR in HEK-293T cells. Anti-Flag immunoprecipitates were analysed by SDS–PAGE. (**e**) HA- p38δ co-immunoprecipitates with endogenous p38γ. p38γ immunoprecipitates from HEK-293T cells transfected with HA-p38δ were analysed by SDS–PAGE. IP, immunoprecipitation; TL, total lysate.

We next investigated whether p38γ/δ isoforms interact with components of the mTOR complexes. Immunoblot analysis of immunoprecipitated p38γ from mouse embryonic fibroblasts (MEFs) surprisingly detected co-immunoprecipitation of several components of both mTORC1 and mTORC2 (Fig. 4a). To hone the search for specific interactions, we focused on mTOR and DEPTOR, components common to the mTORC1 and mTORC2 complexes. DEPTOR has an inhibitory action on the mTOR pathway and contains a PDZ domain^11^, which has the potential to interact with the C-terminal of p38γ, as occurs with other PDZ-containing proteins^30^,^31^. To test DEPTOR–p38γ interaction, we transfected HEK-293 cells with HA-tagged p38γ, Flag-tagged DEPTOR and myc-tagged mTOR, alone and in combination. p38γ co-immunoprecipitated with DEPTOR regardless of whether the cells co-expressed mTOR (Fig. 4b); in contrast, p38γ only co-immunoprecipitated with mTOR when it was expressed together with DEPTOR (Fig. 4c), thus indicating that p38γ is a DEPTOR-interacting protein. To determine which DEPTOR domain interacts with p38γ, we transfected HEK-293 cells with HA-tagged p38γ and Flag-tagged DEPTOR domains (the DEP domain or PDZ domain). p38γ interacted with the PDZ domain and not the DEP binding site (Supplementary Fig. 4b). To determine whether DEPTOR also interacts with p38δ, we transfected HEK-293 cells with Flag-DEPTOR and either HA-p38γ or HA-p38δ. Although both kinases co-immunoprecipitated with DEPTOR, the interaction with HA-p38γ appeared to be stronger (Fig. 4d). However in MEF, we detected co-immunoprecipitation of DEPTOR with p38δ (Supplementary Fig. 4a). Moreover, *in vitro* analysis indicated direct interaction of p38γ and p38δ with DEPTOR (Supplementary Fig. 4d). Surprisingly neither the PDZ nor DEP domains are sufficient for this interaction, because while p38δ interacted with whole DEPTOR, it did not bind the individual DEP or PDZ domains (Supplementary Fig. 4c). Moreover, HA-p38δ co-immunoprecipitated with endogenous p38γ, indicating that p38γ and p38δ form part of the same complex (Fig. 4e).

### p38γ and p38δ modulate DEPTOR protein levels

**Figure 5:**
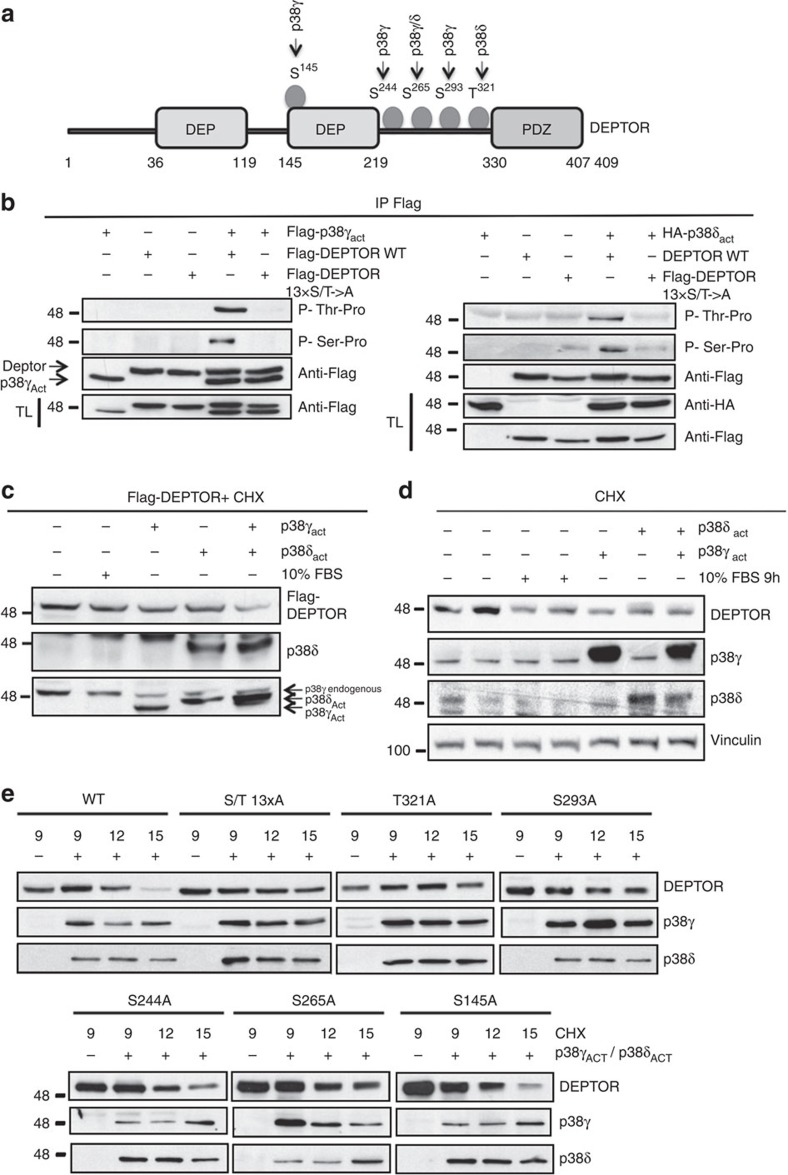
p38γ and p38δ phosphorylate and downregulate DEPTOR protein levels. (**a**) Structural organization of DEPTOR, indicating p38γ and p38δ phosphorylation sites found by *in vitro* kinase assay. (**b**) p38γ and p38δ phosphorylate native DEPTOR on the canonical serine-proline MAPK phosphorylation residues *in vivo*. p38γ or p38δ active mutants were co-expressed in HEK-293T cells with Flag-DEPTOR or Flag-13xS/T→A DEPTOR (a mutated form with alanine substitutions of the S/T target residues). Flag-DEPTOR proteins were immunoprecipitated from cell lysates. Immunoprecipitates were analysed by SDS–PAGE and blotted with anti-phospho-threonine-proline and anti-phospho-serine-proline antibody; TL, total lysate. (**c**,**d**) Constitutively active p38γ and p38δ mutants induce DEPTOR degradation. (**c**) Flag-DEPTOR was expressed in HEK-293T cells alone or together with constitutively active p38γ and p38δ mutants, singly or together. HEK-293T cells starved for 30 h were incubated for 9 h with 10 μM cycloheximide (CHX) with or without 10% FBS. Cell lysates were analysed by immunoblotting with the indicated antibodies. (**d**) Endogenous DEPTOR levels are reduced when the active p38γ and p38δ mutants are overexpressed in HELA cells. HELA cells were serum-starved for 30 h and treated as in **c**. (**e**) DEPTOR phosphorylation mutants were expressed in HEK-293T alone or together with constitutively active p38γ and p38δ mutants. Fresh media without serum was added together with 10 μM cycloheximide (CHX) and cells collected at the times indicated. DEPTOR degradation was analysed by immunoblot.

DEPTOR contains several potential MAPK phosphorylation sites (S/T-P), located outside the PDZ domain. An *in vitro* kinase assay showed p38γ-mediated phosphorylation of DEPTOR at four residues (Ser145, Ser244, 265 and Ser293) and p38δ-mediated phosphorylation at two (Ser265 and Thr321); no phosphorylation by p38α was detected (Fig. 5a, Supplementary Fig. 5a). DEPTOR phosphorylation on (S/T-P) residues in live cells was confirmed by immunoprecipitation/immunoblot analysis of HEK-293 cells transfected with DEPTOR or DEPTOR (13xS/T→A) mutant together with constitutively active p38γ or p38δ mutants (Fig. 5b) and was confirmed by mass spectrometry to selected peptides (Supplementary Table 1 and Supplementary Fig. 5a,b).

DEPTOR levels increase in response to serum deprivation, resulting in mTOR inhibition, whereas serum stimulation leads to phosphorylation-dependent degradation of DEPTOR and enhanced mTOR activity^11^,^32^. To investigate whether p38γ or p38δ promote mTOR activity by triggering DEPTOR degradation, we blocked translation of new protein with cycloheximide in HEK 293 cells transfected with DEPTOR and the active p38 mutants. Active p38γ or p38δ reduced DEPTOR protein levels to a similar extent as serum stimulation (Fig. 5c, Supplementary Fig. 6a), and the effect was stronger in cells co-transfected with both kinases (Fig. 5c). Similar results were obtained for endogenous DEPTOR in HeLa cells (Fig. 5d, Supplementary Fig. 6b). Moreover, the DEPTOR (13xS/T→A) mutant was not degraded, confirming that DEPTOR degradation was phosphorylation-dependent (Supplementary Fig. 6c). We next determined which phosphorylation sites in DEPTOR are necessary for its degradation by generating single-point DEPTOR mutants through the substitution of serine/threonine with alanine in codons 145, 244, 265, 293 or 321 (Supplementary Table 2). The protein half-life of two mutants (S293A and T321A) was extended to the same level as that of the mutated DEPTOR (13xS/T→A) mutant (Fig. 5e and Supplementary Fig. 6d), whereas a more moderate half-life extension was observed for S244A and S265A mutants (Fig. 5e and Supplementary Fig. 6d). These data indicate that p38γ- and p38δ-mediated phosphorylation of key residues targets DEPTOR for degradation. DEPTOR is a substrate of SCF E3 ubiquitin ligase^33^, and DEPTOR poly-ubiquitination is dependant on DEPTOR phosphorylation^33^. Then we assayed whether phosphorylation *in vitro* of DEPTOR by p38γ or p38δ promote ubiquitination of DEPTOR. Our results indicate that DEPTOR presented higher ubiquitination after *in vitro* phosphorylation by p38γ and p38δ (Supplementary Fig. 6e). Moreover, we observed that in HEK293, active p38γ or p38δ significantly enhanced poly-ubiquitination of native DEPTOR but not the non-phosphorylatable DEPTOR (13xS/T→A) mutant (Supplementary Figs 6f and 7a).

**Figure 6:**
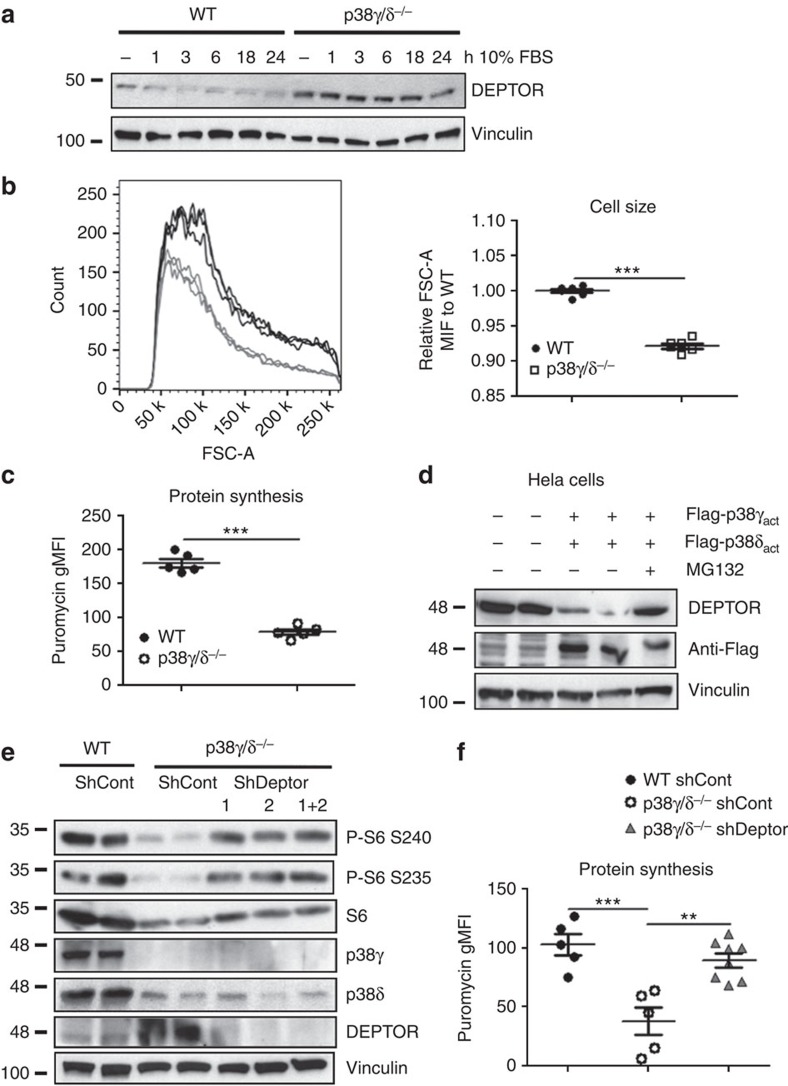
p38γ/δ control cell size and protein synthesis through DEPTOR levels. (**a**) p38γ/δ^−/−^ MEFs present altered serum-induced DEPTOR degradation. WT and p38γ/δ^−/−^ MEFs were serum-starved for 30 h, followed by serum addition. Cells were collected at successive time points for immunoblotting with the indicated antibodies. (**b**) p38γ/δ^−/−^ MEFs are of below-normal size. Cell size was measured by flow cytometry (forward scatter). Right: representative histogram. Left: quantification graph of the forward scatter mean fluourescence intensity (FSC-A MFI) relative to WT. Data are means±s.e.m. ***P<0.001 (*t*-test). (**c**) p38γ/δ^−/−^ MEFs have downregulated protein synthesis. SUnSET was performed by pulsing 10 min 10 μg ml^−1^ puromycin and chasing for 1 h before FACS analysis with anti-puromycin 12D10 antibody and anti-mouse IgG conjugated with PE. Data are means±s.e.m. ***P<0.001 (*t*-test). (**d**) p38γ/δ-induced DEPTOR degradation by the proteasome. HELA cells co-transfected with active p38γ and p38δ mutants were serum-starved for 30 h. Cells were treated with MG132 (10 μM) or vehicle together with 10 μM cycloheximide (CHX) for 9 h, and were analysed by immunoblotting with the indicated antibodies. (**e**) Silencing DEPTOR in p38γ/δ^−/−^ MEF cells restores mTOR signalling. MEFs were singly or doubly infected with two different DEPTOR lentiviral shRNA constructs for 24 h. Uninfected cells were eliminated by selection with 3 μg ml^−1^ puromycin for 1 week. The resulting cell lines were then serum-starved for 24 h before collecting. Equal amounts of whole-cell lysates were immunoblotted with the indicated antibodies. (**f**) Silencing of DEPTOR in p38γ/δ^−/−^ MEFs increases protein synthesis. MEFs were infected as in **e**. In the resulting cell lines, the protein concentration per cell was measured by SUnSET assay, performed as in **b**. Data are means±s.e.m. **P<0.01; ***P<0.001 (one-way analysis of variance coupled to Bonferroni post tests).

To determine whether p38γ is involved in DEPTOR degradation after serum stimulation, we first verified the activation of the kinase in this setting (Supplementary Fig. 7b). MEFs lacking p38γ/δ had above-normal levels of DEPTOR and were resistant to DEPTOR degradation after serum stimulation (Fig. 6a). No changes were found in the mTOR complex components Raptor or Sin1 (Supplementary Fig. 7c). The above-normal and serum-independent DEPTOR content in p38γ/δ^−/−^ cells correlated with impaired mTOR activation, measured by below-normal phosphorylation of p70S6K and its target S6 (Supplementary Fig. 7c). Moreover, the serum-independent DEPTOR content in these cells correlated with increased binding to mTOR (Supplementary Fig. 7d). Consistent with impaired mTOR pathway activation (Supplementary Fig. 7e), p38γ/δ^−/−^ MEFs had lower protein synthesis activity and were smaller than WT counterparts (Fig. 6b,c).

To confirm whether p38γ/δ isoforms trigger DEPTOR degradation through a posttranscriptional mechanism, we treated MEFs with the protein synthesis inhibitor cycloheximide (CHX). DEPTOR levels in WT MEFs declined after 12 h exposure to CHX, whereas high DEPTOR levels were sustained in p38γ/δ null MEFs after 24 h (Supplementary Fig. 7f). Consistent with these results, DEPTOR levels in HeLa cells were reduced by expression of the constitutively active p38γ and p38δ mutants, and this effect was blocked by the proteasome inhibitor MG132 (ref. 34; Fig. 6d). As expected, shRNA-mediated suppression of DEPTOR in WT cells resulted in greater mTOR activation, protein synthesis and in consequence increased cell size (Supplementary Fig. 8a–c). Interestingly, treatment with DEPTOR shRNA in p38γ/δ^−/−^ cells restored WT mTOR activity, protein synthesis and cell size (Fig. 6e,f, Supplementary Fig. 8d). These results thus suggest that p38γ/δ isoforms are key regulators of DEPTOR degradation by the proteasome, thereby promoting mTOR-dependent protein synthesis and cell growth.

### p38γ/δ regulation of mTOR activity controls heart hypertrophy

**Figure 7:**
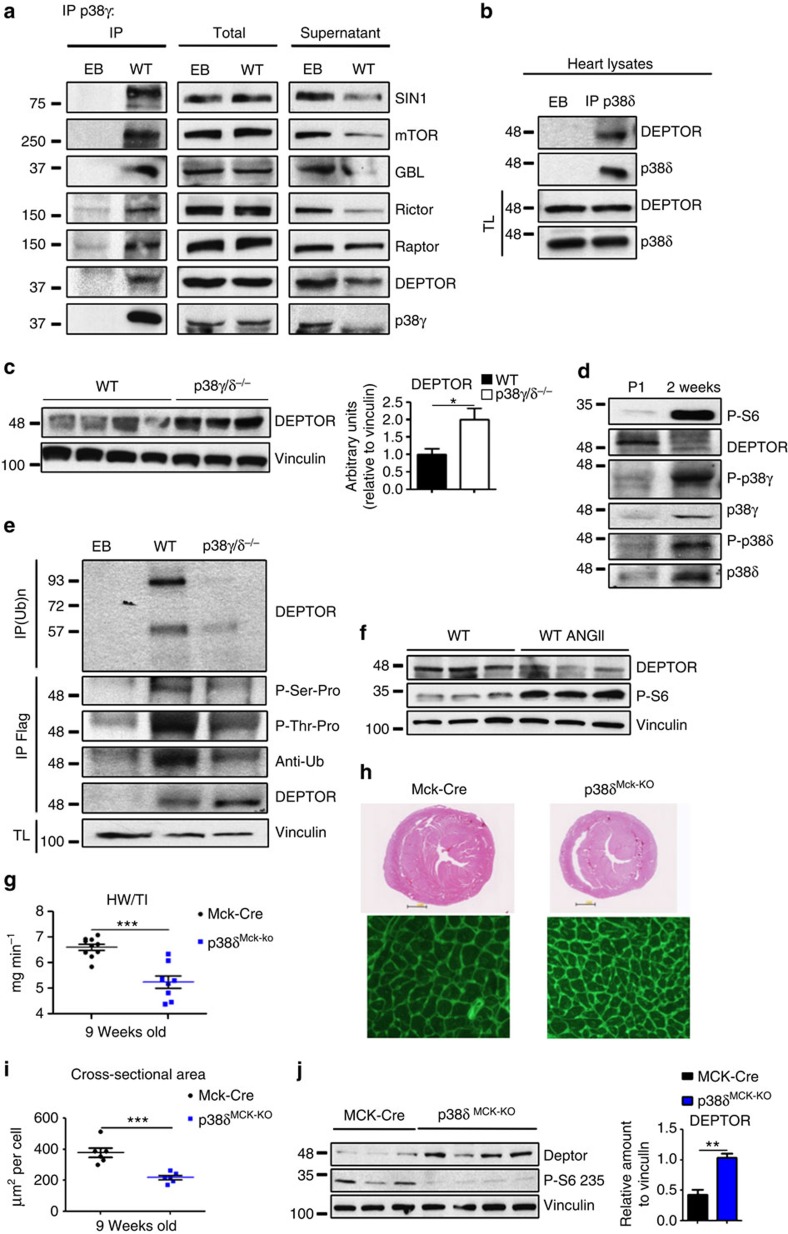
Cardiac p38γ/δ activation correlates negatively with DEPTOR levels. (**a**) Endogenous cardiac Sin-1, mTOR, GβL, Rictor, Raptor and DEPTOR co-immunoprecipitate with endogenous p38γ. p38γ immunoprecipitates (IP), total lysates and Co-IP supernatants from WT heart lysates were analysed by SDS–PAGE. EB: beads with IgG control. (**b**) Endogenous cardiac DEPTOR co-immunoprecipitates with p38δ. Immunoblot analysis of p38δ immunoprecipitates (IP) and total lysates (TL) from the hearts of 9-week-old p38γ/δ^−/−^ mice infected with AAV-TnT-p38γ_act_ and AAV-TnT-p38δ_act_. (**c**) p38γ/δ^−/−^ hearts express above-normal levels of DEPTOR protein. Immunoblot analysis of heart lysates from WT and p38γ/δ^−/−^ mice starved for 4 h and re-fed for 2h (*n*=3–4). (**d**) Cardiac DEPTOR levels during postnatal development correlate negatively with p38γ and p38δ activation and mTOR pathway activation. Heart lysates from WT p1 and 2-week-old mice were analysed by immunoblot (*n*=6). (**e**) Levels of DEPTOR phosphorylation and ubiquitination *in vivo* are reduced in p38γ/δ^−/−^ hearts. Upper panel: poly-ubiquitinated proteins were IP from WT and p38γ/δ^−/−^ heart lysates and immunoprecipitates were immunoblotted with anti-DEPTOR antibody. Lower panels: WT and p38γ/δ^−/−^ mice were intravenously injected with AAV-TNT-Flag-DEPTOR and hearts harvested at the age of 2 weeks. Flag-DEPTOR was immunoprecipitated from heart lysates, and immunoprecipitates were analysed by immunoblotting with the indicated antibodies. (*n*=5). (**f**) Angiotensin II (ANGII) treatment induces a reduction in DEPTOR levels in WT hearts. WT mice were treated with ANGII or saline for 21 days. Heart lysates were analysed by immunoblotting. (*n*=3). (**g**–**i**) MCKdelta KO mice have small hearts. (**f**) Heart-weight-to-tibia-length ratios in WT and MCKdelta KO (p38δ^MCK−KO^) mice killed at 9 weeks. (**g**) Top: representative haematoxylin and eosin staining of transverse heart sections from 9-week-old WT and MCKdelta KO mice. Bottom: representative staining with FITC-WGA (green) in hearts from 9-week-old WT and MCKdelta KO mice. (**h**) Cardiomyocyte cross-sectional area quantified from WGA-stained hearts. (**j**) MCKdelta KO hearts have higher protein levels of DEPTOR. MCK-Cre control mice and MCKdelta KO (p38δ^MCK−KO^) mice were starved for 4 h before being killed and tissue was collected. Heart lysates were analysed by immunoblotting; the bar chart shows quantification of vinculin-normalized band intensities (ImageJ; *n*=4). Data are means±s.e.m. (*n*=5). **P<0.01; ***P<0.001 (*t*-test).

To determine whether p38γ and p38δ interact with cardiac mTOR complexes *in vivo*, we immunoprecipitated p38γ and p38δ from heart extracts. The immunoprecipitates contained several mTORC1 and mTORC2 components, including DEPTOR (Fig. 7a,b Supplementary Fig. 9a,b). Moreover, p38γ immunoprecipitates contained p38δ and vice versa, confirming that the two kinases form part of the same complex *in vivo* (Supplementary Fig. 9c,d). Further analysis in heart extracts of p38γ^−/−^ and p38δ^−/−^ mice revealed that each kinase is needed for the interaction of the other in the mTOR complexes (Supplementary Fig. 9c), providing a probable explanation for the shared phenotype of p38γ and p38δ null mice.

The control of heart size by p38γ/δ-induced DEPTOR degradation in cardiomyocytes predicts that hearts from p38γ and p38δ null mice will present higher protein levels of DEPTOR, and this was confirmed by immunoblot analysis of hearts from 9-week-old animals (Fig. 7c). Interestingly, DEPTOR mRNA levels were below normal in p38γ and p38δ null hearts, indicating that the increased DEPTOR protein expression is not owing to higher DEPTOR transcription (Supplementary Fig. 9e). Moreover, DEPTOR levels were strongly reduced from P1 to 2 weeks in WT hearts, a period in which the heart switches from proliferation to hypertrophic growth. This correlated with maximal activation of p38γ and p38δ, and with the activation of mTOR (Fig. 7d). Moreover, DEPTOR phosphorylation and ubiquitination in WT mice were increased during this period, whereas phosphorylation and ubiquitination were reduced in p38γ and p38δ null mice (Fig. 7e). In WT mice, angiotensin II, which activates p38γ and p38δ (Fig. 2a), also correlated with DEPTOR degradation and mTOR activation (Fig. 7f). These results suggest that p38γ and p38δ modulate heart growth *in vivo* by controlling DEPTOR degradation and in consequence mTOR activity.

**Figure 8:**
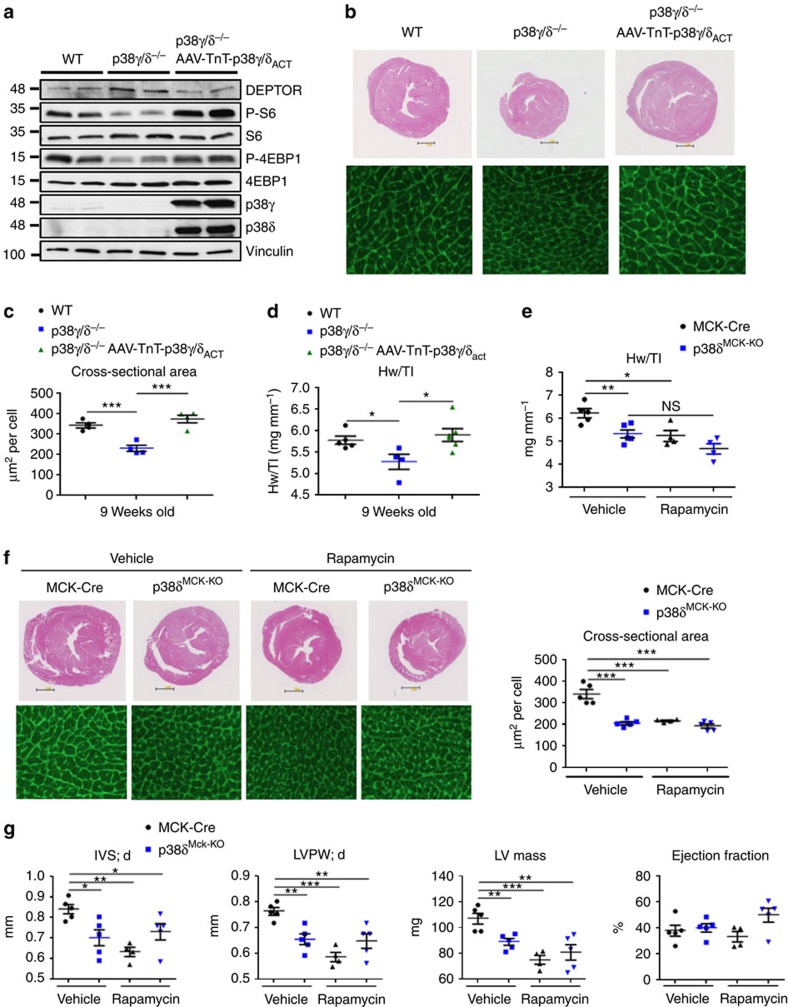
Cardiac p38γ/δ control heart growth by modulating mTOR activity. (**a**–**d**) Cardiac-specific expression of active p38γ and p38δ mutant forms in p38γ/δ^−/−^ hearts restores normal heart size. p38γ/δ^−/−^ mice were intravenously injected at 4 weeks of age with AAV-TnT-p38γ_act_ and AAV-TnT-p38δ_act_, and hearts were harvested from 9-week-old WT, p38γ/δ^−/−^ and p38γ/δ^−/−^ AAV-TnT-p38γ/δ_act_ mice. (**a**) Immunoblot analysis of heart lysates. (**b**) Top: representative haematoxylin and eosin (H&E) staining of transverse heart sections. Bottom: representative staining with FITC-WGA (green). (**c**) Cardiomyocyte cross-sectional area quantified from WGA-stained hearts. (**d**) Heart-weight-to-tibia-length ratio. (**e**–**g**) Rapamycin treatment preserves normal heart size in MCKdelta KO hearts. MCK-Cre (control) and MCKdelta KO (p38δ^MCK−KO^) mice were intraperitoneally injected daily with rapamycin (2 mg kg^−1^ per day) from 4 to 9 weeks of age. (**e**) Heart-weight-to-tibia-length ratio. (**f**) Top: representative H&E stained transverse heart sections from 9-week-old MCK-Cre and MCKdelta KO mice after rapamycin treatment. Bottom: representative FITC-WGA staining (green) in hearts from 9-week-old MCK-Cre and MCKdelta KO mice after rapamycin treatment. (**g**) Echocardiography analysis of 9-week-old MCK-Cre and MCKdelta KO mice treated with rapamycin or vehicle. IVS;d (interventricular septum in diastole); LVPW;d (left ventricle posterior wall in diastole); LV Mass (left ventricle) and ejection fraction. Data are means±s.e.m. (*n*=5). *P<0.05; **P<0.01; ***P<0.001 (one-way analysis of variance coupled to Bonferroni post test). NS, not significant.

To determine whether the impaired heart growth in the p38γ/δ^−/−^ animals results from an autonomous effect on postnatal cardiomyocyte growth, we generated mice lacking p38δ in striated muscle by crossing mice carrying *lox*P-flanked *p38δ* with MCK Cre mice (p38δ^Mck−KO^), which reach peak Cre expression on postnatal day 10 (ref. 35). The cardiac phenotype of mice lacking p38δ specifically in striated muscle resembled that of p38δ^−/−^ mice, suggesting that p38δ-mediated control of heart growth is cell autonomous (Fig. 7g–j and Supplementary Fig. 10a). To further confirm that the phenotype is cardiac specific, we treated p38γ/δ^−/−^ mice with cardiac-specific adeno-associated viruses expressing p38γ/δ active mutants under the troponin T promoter (AAV-TnT-p38γ/δact). Cardiac-specific expression of p38γ/δ-active mutant reduced DEPTOR levels in p38γ/δ^−/−^ heart to the levels of WT mice and increased mTOR activation (Fig. 8a). In consequence, reduction of heart growth was reverted in p38γ/δ^−/−^ mice treated with AAV-TnT-p38γ/δact and cardiomyocyte cross-sectional area increased to WT levels (Fig. 8b,c).

To confirm whether p38-mediated modulation of the mTOR pathway is responsible for normal postnatal heart growth, we treated MCK-cre and p38δ^Mck-KO^ mice with the mTOR inhibitor rapamycin from weeks 4 to 9 after birth. Rapamycin treatment reduced the heart weight and cardiomyocyte size only in control mice (MCK-cre), equalizing with heart weight in p38δ^Mck-KO^ mice (Fig. 8e,f). Moreover, echocardiographic studies showed that rapamycin reduced diastolic IVS, LVPW and LV mass in MCK-Cre mice but not p38δ^Mck-KO^ mice (Fig. 8g), without between-genotype differences in ejection fraction (Fig. 8g). Thus the small hearts in p38δ^Mck-KO^ and p38γ/δ KO mice appear to result from the inhibition of mTOR activation in these mice.

**Figure 9:**
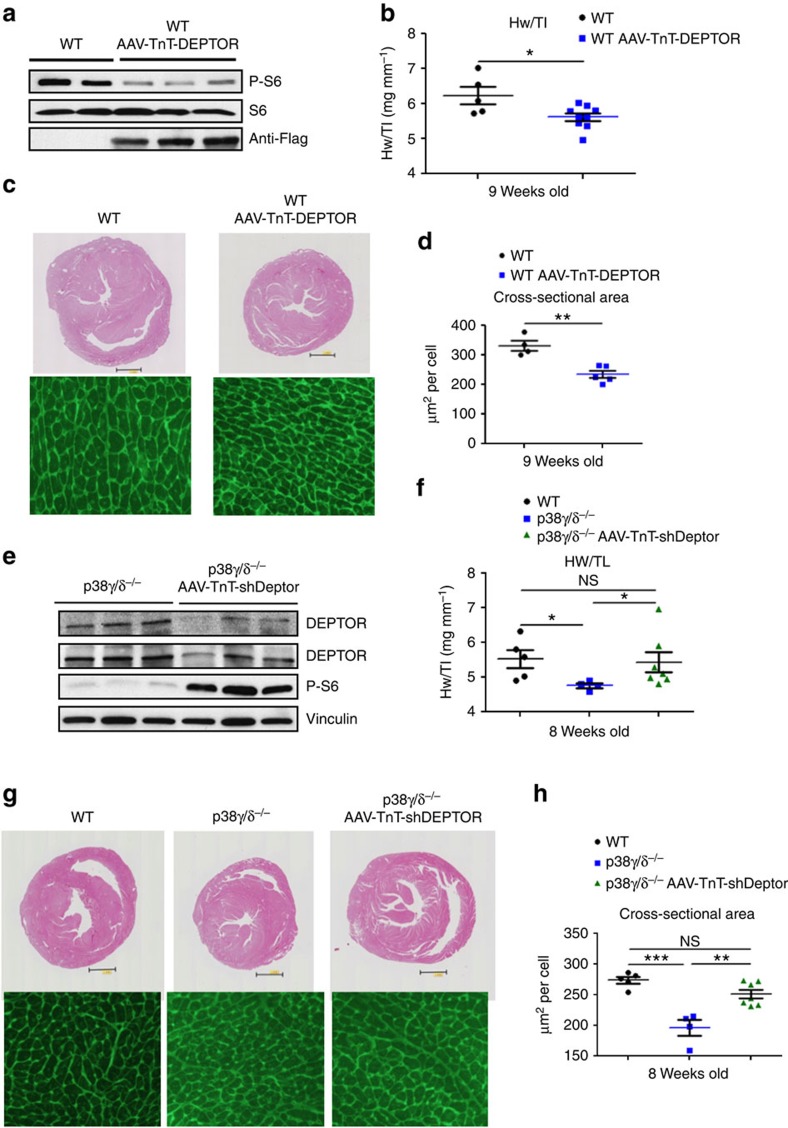
Deptor cardiac specific expression reduces heart size. (**a**–**d**) Cardiac-specific expression of Flag-DEPTOR in WT hearts reduces heart size. WT mice were intravenously injected at 4 weeks of age with AAV-TnT-Flag-DEPTOR and hearts harvested at 9 weeks of age. (**a**) Immunoblot analysis of heart lysates. (**b**) Heart-weight-to-tibia-length ratio (**c**) Top: representative haematoxylin and eosin (H&E) staining of transverse heart sections from untreated (WT) and AAV-TnT-Flag-DEPTOR-injected mice. Bottom: representative staining with FITC-WGA (green). (**d**) Cardiomyocyte cross-sectional area quantified from WGA-stained heart. (*n*=5–9). Data are means±s.e.m. (*n*=5). *P<0.05; **P<0.01 (*t*-test). (**e**–**h**) Cardiac-specific DEPTOR silencing in p38γ/δ^−/−^ mice restores normal heart size. Hearts from WT, p38γ/δ^−/−^ and p38γ/δ^−/−^ mice intravenously injected at birth with AAV-TnT-shDeptor were harvested at 9 weeks of age. (**e**) Immunoblot analysis of heart lysates. (**f**) Heart-weight-to-tibia-length ratio (**g**) Top: representative H&E staining of transverse heart sections from untreated WT and p38γ/δ^−/−^ and from AAV-TnT-shDeptor injected p38γ/δ^−/−^ mice. Bottom: representative staining with FITC-WGA (green). (**h**) Cardiomyocyte cross-sectional area quantified from WGA-stained hearts. (*n*=5–7). Data are means±s.e.m. (*n*=5). *P<0.05; **P<0.01; ***P<0.001 (one-way analysis of variance coupled to Bonferroni post test). NS, not significant.

To test whether decreased mTOR signalling could account for the weak heart growth in p38γ/δ^−/−^ mice, we hyperactivated mTOR with aminoacids (aa) to restore heart growth in p38γ/δ KO mice. As expected, daily aa injection activated the mTOR pathway in p38γ/δ^−/−^ hearts (Supplementary Fig. 10b). Compared with saline treatment, aa supplementation significantly increased heart size, LV mass and cardiomyocite cross-sectional area (Supplementary Fig. 10c–f). Consistently, cardiac-specific overexpression of DEPTOR in WT mice resulted in reduced mTOR activation and lower heart mass, correlating with the presence of cardiomyocytes of below-normal size, determined from cell cross-sectional area on histological sections (Fig. 9a–d).

To confirm that decreased DEPTOR degradation in cardiomyocytes contributes to the phenotype of p38γ/δ KO mice, we used DEPTOR-targeted shRNA under the promoter of cardiac troponin T (AAV-TnT-shDEPTOR). Cardiac-specific reduction of DEPTOR expression in p38γ/δ^−/−^ mice was confirmed by western blot and was sufficient to restore mTOR activation (Fig. 9e). Furthermore, these animals showed re-established cardiac growth and increased cardiomyocyte cross-sectional area (Fig. 9f–h). Moreover, a control, AAV-Tnt or AAV-TnT-shDEPTOR did not affect cardiac growth in WT mice (Supplementary Figs 11 and 12). These results confirm that reduction of heart growth observed in p38γ/δ^−/−^ mice is driven by the accumulation of DEPTOR caused by the absence of DEPTOR phosphorylation and degradation. DEPTOR accumulation inhibits mTOR pathway and consequently growth.

## Discussion

In this study, we provide evidence that two stress kinases, p38γ and p38δ, promote the activity of mTOR, a critical sensor of metabolic and nutrient stresses, by targeting the mTOR inhibitory partner DEPTOR for proteasome degradation. The finding that DEPTOR is a physiological p38γ/δ substrate is, to our knowledge, the first demonstration of DEPTOR interaction with a non-mTOR complex protein. Moreover, our results show that p38γ or p38δ are both able to phosphorylate DEPTOR and induce its subsequent proteasomal degradation, contrary to the proposal that DEPTOR phosphorylation was only mTOR dependent^11^. The p38γ/δ-mTOR signalling pathway controls protein synthesis and cell growth, and molecular and functional evidence indicates that this pathway plays an important physiological role in the regulation of ventricular cardiac hypertrophy.

Our conclusion is supported by several lines of evidence: (1) mTOR activity is below-normal in hearts and cells from mice deficient in p38γ and p38δ; (2) low mTOR activation in cells lacking p38γ and p38δ leads to reduced cell size and protein synthesis; (3) p38γ and p38δ co-immunoprecipitate with mTORC1 and mTORC2 complexes; (4) p38γ and p38δ bind to DEPTOR; (5) DEPTOR is phosphorylated by p38γ and p38δ; (6) DEPTOR protein half-life is shortened by active p38γ and p38δ mutants, but extended by knockout of p38γ and p38δ; (7) DEPTOR levels are elevated in hearts lacking p38δ or p38γ/δ; (8) reduction of DEPTOR levels correlates with p38γ and p38δ activation; (9) silencing of DEPTOR in cells lacking p38γ and p38δ increases their protein content and size; (10) rapamycin treatment in the postnatal period equalizes heart size in WT and KO animals; (11) mTOR activation by aa injection into p38γ/δ^−/−^ mice results in heart growth; (12) cardiac-specific overexpression of active p38γ/δ reduces DEPTOR levels, increasing mTOR activity and cardiomyocyte growth; (13) cardiac overexpression of DEPTOR phenocopies the p38γ/δ^−/−^ phenotype; (14) cardiac-specific silencing DEPTOR in p38γ/δ^−/−^ mice increases their heart size and cardiomyocyte growth.

Despite the physiological and pathological importance of cell-size regulation, our understanding of the underlying mechanisms remains very limited. In the heart, the regulation of cell size is a key process that modulates the organ’s response to external stimuli. Identifying molecular mechanisms through which hypertrophy can be suppressed without provoking circulatory insufficiency is a major challenge. Our data strongly indicate that lack of p38γ and p38δ reduces the capacity for cardiomyocyte growth without having functional implications: cardiac function remained normal in these mice even in the presence of AngII-induced hypertrophy, and they showed no increase in fibrosis with respect to WT mice.

Surprisingly, although mTOR is necessary for embryonic cardiovascular development and for the postnatal maintenance of cardiac function^8^, inhibition of mTOR as a result of the lack of p38γ and p38δ does not have a deleterious effect on heart physiology in basal condition. This is likely because p38γ and p38δ act through the mTOR modulator DEPTOR, so that the lack of p38γ and p38δ results only in partial inhibition of mTOR activity. This finding is of great interest because other models in which the heart is incapable of growing display signs of cardiac pathology such as cardiac dilation or heart failure^36^,^37^. The fact that the modulation of these kinases results in reduced heart hypertrophy without obvious secondary effects makes p38γ and p38δ potential targets for therapeutic intervention. However, it would be interesting to further study whether modulation of these kinases could have functional implication after a stronger challenge such as myocardial infarction.

Our findings provide definition of the previously uncharacterized role of p38γ and p38δ in cardiac hypertrophy^38^, demonstrating that p38γ and p38δ are activated during postnatal growth and by stress stimuli in the heart. This activation correlates with DEPTOR phosphorylation, ubiquitination and degradation, modulating cardiac growth in tissue culture and animal models. Mice deficient in these kinases presented reduced DEPTOR phosphorylation and degradation, correlating with impaired postnatal and pathologically induced cardiac hypertrophy.

The finding that lack of just one of these kinases produces the defect is quite surprising because each normally compensates the loss of the other in other biological processes. The non-redundancy of these isoforms in the induction of cardiac hypertrophy is likely explained by their interaction *in vivo*. Both kinases cooperate in the binding to the mTOR complex and in the modulation of its activity through the phosphorylation and subsequent ubiquitination and proteasomal degradation of DEPTOR. Although p38γ interacts with DEPTOR through the PDZ domain, the exact interaction between DEPTOR and p38δ remains unclear. Moreover, our experiments with single-point mutants indicated that both kinases are needed to induce DEPTOR degradation. Lack of phosphorylation on S293 or T321, residues respectively phosphorylated by p38γ and p38δ, are sufficient to abolish DEPTOR degradation, indicating a requirement for cooperation between both kinases to induce DEPTOR degradation. Moreover, mTOR induces DEPTOR phosphorylation on other residues, which are also necessary for DEPTOR ubiquitination and degradation^11^,^32^,^33^. Additional studies will be needed to clarify how these kinases cooperate to phosphorylate DEPTOR and to determine whether p38 γ and δ act as a heterodimer. Moreover it would be interesting to study whether this mechanism acts in other tissues and in response to other stimuli.

Cell autonomous control of cell hypertrophy by p38γ and p38δ was validated by several independent approaches, including the use of mice lacking p38δ in myocytes, and immortalized fibroblasts from p38γ/δ^−/−^ mice. Furthermore, hearts from p38γ/δ null animals are able to grow after AAV-mediated cardiomyocyte overexpression of p38γ and p38δ, and AAV-mediated DEPTOR overexpression in WT hearts reduces cardiomyocyte size. Finally, reduction of DEPTOR levels in cardiomyocytes from p38γ/δ null animals increased heart growth to the level seen in WT mice, indicating that the phenotype found in p38γ/δ null animals is mediated by DEPTOR.

Our results identify p38γ and p38δ signalling as a key regulator of mTOR activity, promoting protein synthesis and cell hypertrophy by phosphorylating the mTOR inhibitor DEPTOR and inducing its ubiquitination and degradation. p38γ/δ signalling through mTOR complexes is an important mediator of cardiac hypertrophy, and this pathway may also be important in other physiological processes mediated by mTOR, such as cancer and autophagy. These results reveal a new avenue in the control of mTOR activation and open a route to the development of new treatment strategies for disease.

## Methods

### Mice

Mice deficient for p38γ (B6.129-Mapk12tm1) and p38δ (B6.129-Mapk13tm1) were backcrossed for 10 generations to the C57BL/6J background (Jackson Laboratory). To generate mice lacking p38δ in striated muscle, p38δ (B6.129-Mapk13tm1) mice were crossed with the FVB-Tg(Ckmm-cre)5Khn/J line on the C57BL/6J background (Jackson Laboratory). Genotype was confirmed by PCR analysis of genomic DNA. For signalling studies, animals were killed by cervical dislocation. For AngII experiments, 9-week-old male mice were treated with AngII (1 μg kg^−1^ per minute, 28 days) via subcutaneously implanted mini-osmotic pumps (Alzet); saline was administered as a control. For rapamycin treatment, mice were daily injected intraperitoneally with rapamycin (LC Laboratories, R-5000) (2 mg kg^−1^ per day) or vehicle (0.25% polyethylene glycol (Sigma), 0.25% Tween-20 (Sigma) in PBS); injections started at 4 weeks of age and continued until 9 weeks of age, when heart size was analysed by echocardiography and mice were killed. Amino acid (aa) supplementation was achieved by daily intraperitoneal injections, starting at the day of birth, of aa solution at a dose of 0.1 ml g^−1^ body weight, with a maximal dose of 2 ml per day^39^. All aa were purchased from Sigma-Aldrich. The solution contained the following aa concentrations: 6.0 g l^−1^ isoleucine, 12.0 g l^−1^ leucine, 7.2 g l^−1^ valine, and 6.04 g l^−1^ arginine (pH 7.4; Sigma). PBS (pH 7.4) was used as a control. Four-week-old mice were intravenously injected with 1 × 10^12^ viral particles encoding human p38γ and p38δ active mutants or wild-type human Flag-DEPTOR under the TnT promoter. Hearts were harvested from 9-week-old mice. Day-old mice were intravenously injected with 0.5 × 10^12^ viral particles encoding wild-type human Flag-DEPTOR under the TnT promoter and hearts were harvested for analysis of DEPTOR phosphorylarion/ubiquitination at 2 weeks of age. To avoid DEPTOR degradation, mice were injected the proteasome inhibitor MG132 (Shelleckchem; 0.1 mg kg^−1^ per day intraperitoneally) on the 2 days before harvesting the hearts. In the cardiac-specific DEPTOR silencing experiment, 1-day-old mice were intravenously injected with 0.5 × 10^12^ viral particles encoding shDEPTOR and the cardiac phenotype was studied at 8 weeks of age. All animal procedures conformed to EU Directive 86/609/EEC and Recommendation 2007/526/EC regarding the protection of animals used for experimental and other scientific purposes, enacted under Spanish law 1201/2005.

### Histology

Tissue samples were fixed in 10% formalin for 48 h, dehydrated and embedded in paraffin. Sections (8 μm) were cut and stained with haematoxylin and eosin (American Master Tech Scientific). Fibrosis was assessed with Picrosirius red staining (Sigma). For wheatgerm agglutinin (WGA) immunofluorescence, 8 μm heart sections were prepared, washed in PBS, incubated for 2 h in WGA-Alexa 488 lectin (Invitrogen, Carlsbad, CA, USA), and washed and mounted in anti-fade reagent. Four images ( × 20) were taken from each heart, and the diameter and areas of 100–200 cross-sectionally oriented myocytes were measured and analysed with *Image J* software.

### Echocardiography

Mice were anaesthetized by inhalation of isoflurane and oxygen (1.25% and 98.75%, respectively), and echocardiography was performed with a 30-MHz transthoracic echocardiography probe. Images were obtained with the Vevo 2100 micro-ultrasound imaging system (VisualSonics, Toronto, Canada). Short-axis, long-axis, B-mode and two-dimensional M-mode views were obtained. In summary, scans were conducted by two experienced researchers blinded to the mouse genotype. Measurements of left parasternal long and short axes and M-mode (left parasternal short axis) images were obtained at a heart rate of 500–550 b.p.m. LV end-diastolic diameter (LVEDD), LV end-systolic diameter (LVESD) and wall thickness were measured from M-mode tracings, and the average of three consecutive cardiac cycles is reported. The LV fractional shortening percentage was calculated as ([LVEDD−LVESD]/LVEDD) × 100 MRI of lung was performed with a 7-T Agilent scanner (Agilent, Santa Clara, CA, USA) equipped with a DD2 console and an actively shielded gradient set (205/120 insert of maximum 130 mT m^−1^ gradient strength). For image acquisition, we used a combination of volume coil/surface coil coil to enhance signal-to-noise ratio formed by a 72-mm inner diameter quadrature birdcage TX coil (Rapid Biomedical GmBH, Germany) and an actively detuning 30-mm flexible customized surface RX coil (Neos Biotec, Pamplona, Spain). Following a tripilot gradient-echo image, a gradient-echo sequence without gating was used to acquire oblique coronal slices (one to two slices) and axial slices (7–10 slices covering the entire lung, 72-s acquisition time per slice) using the following parameters: TR/TE=6.7/2.2 ms, flip angle=10 degree, bandwidth=100 kHz, field of view=3 × 3 cm, matrix=256 × 128, slice thickness=1 mm (ref. 40). From these images, interventricular septum and left ventricle posterior wall thicknesses and left ventricle corrected mass were determined; the short-axis M-mode quantification was chosen as the most representative. Function was estimated from the ejection fraction and fractional shortening obtained from M-mode views by a blinded echocardiography expert. For ejection fraction measurements, a long- or short-axis view of the heart was selected to obtain an M-mode registration in a line perpendicular to the left ventricular septum and posterior wall at the level of the mitral chordae tendinea.

### Immunoblot analysis

Tissue extracts were prepared in Triton lysis buffer (20 mM Tris (pH 7.4), 1% Triton X-100, 10% glycerol, 137 mM NaCl, 2 mM EDTA, 25 mM β-glycerophosphate, 1 mM sodium orthovanadate, 1 mM phenylmethylsulfonyl fluoride, and 10 μg ml^−1^ of aprotinin and leupeptin), and for co-immunoprecipitation experiments, lysis buffer was supplemented with 0.3% Chaps. Extracts (20–50 μg protein) and immunoprecipitates (prepared from 2–10 mg protein) were examined by immunoblot. For the immunoprecipitation assay, heart extracts were incubated with 4 μg of a specific antibody coupled to protein-G-Sepharose. After incubation overnight at 4 °C with agitation, the captured proteins were centrifuged at 10,000*g*, the supernatants collected, and the beads washed four times in lysis buffer. Beads were boiled for 5 min 95 °C in 10 μl sample buffer. Extracts and immunoprecipitates were examined by SDS–PAGE and blotted with antibodies to the following targets: p38γ and p38δ (refs 41, 42) at 1 μg ml^−1^; vinculin and Flag (Sigma); puromycin (Millipore clone 12D10); HA and Myc (Bethyl Laboratories, Inc.); ubiquitin (ThermoFisher); and phospho-p38, phospho-mTOR (Ser2481), mTOR, Myc-Tag, phospho-p70S6 kinase, p70S6 kinase, phospho-S6 (Ser 235/236), phospho-S6 (Ser 240/244), S6 ribosomal protein, phospho-FOXO1/3a, phospho-eEF2, eEF2, phospho-EIF4E, EIF4E, phospho-EIF4G, EIF4G, phospho-EIF4B, EIF4B, DEPTOR, phospho-MAPK/CDK substrate (PXSP or SPXR/K) (34B2), phospho-Threonine-Proline, Raptor, Rictor, Sin1, GβL, phospho-4EBP1, 4EBP1, phospho-AKT (Ser473) and AKT (Cell Signaling) all were used at 1:1,000. Immunocomplexes were detected by enhanced chemiluminescence (GE Healthcare Lifesciences). Full scans of all blots and gels are available as Supplementary Fig. 13.

### Cell lines and cell culture

HeLa and HEK-293T (American Type Culture Collection (ATCC), USA) cells and mouse embryonic fibroblasts (MEF) were cultured in DMEM supplemented with 10% heat-inactivated fetal bovine serum (FBS; Sigma), glutamine (2 mM) and penicillin/streptomycin (100 μg ml^−1^).

### Lentivirus vector production and cell infection

Lentiviruses were produced by transient calcium phosphate co-transfection of HEK-293T cells was done with the pLKO mouse shRNA 1 DEPTOR (plasmid #21337 Addgene), pLKO mouse shRNA 2 DEPTOR (plasmid #21338 Addgene), together with pΔ8.9 and pVSV-G. Supernatants containing the LV particles were collected 48 and 72 h after removal of the calcium phosphate precipitate and were centrifuged at 700*g* at 4 °C for 10 min and filtered through 0.45-μm filter units (Corning). Once filtered, the virus-containing medium was added (1:1 volume) to MEF culture medium. After incubation for 24 h at 37 °C and 5% CO_2_, the medium was changed, and puromycin selection (3 μg ml^−1^ puromycin) was initiated 48 h after infection. The selection medium was changed every 2 days during 1 week, and the cells were subsequently used for experiments.

### Adeno-associated virus vector production and cell infection

The AAV shuttle and helper plasmids were transfected into HEK 293A cells by calcium-phosphate co-precipitation. A total of 840 μg plasmid DNA (mixed in an equimolar ratio) was used per Hyperflask (Corning) seeded with 1.2 × 108 cells the day before. Seventy-two hours after transfection, the cells were collected by centrifugation and the cell pellet was resuspended in TMS (50 mM Tris HCl, 150 mM NaCl, 2 mM MgCl_2_) on ice before digestion with DNase I and RNaseA (0.1 mg ml^−1^ each; Roche) at 37 °C for 60 min. Clarified supernatant containing the viral particles was obtained by iodixanol gradient centrifugation^44^. Gradient fractions containing virus were concentrated using Amicon UltraCel columns (Millipore) and stored at −70 °C (ref. 43).

AAV plasmids were cloned and propagated in the Stbl3 *E. coli* strain (Life Technologies). pRK5 FLAG human DEPTOR (plasmid #21334, Addgene), pCEFL Flag p38γ D129A, pCMV Flag p38δ F324S and pGIPZ mouse shRNA DEPTOR clone 1 (V3LMM_485571) were cloned into the pAcTnT AAV plasmid to generate pAAV-TnT-GFP-Luc, pAAV-TnT-p38γ_act_, pAAV-TnT-p38δ_act_, pAAV-TnT-Flag-DEPTOR and pAAV-TnT-shDeptor. These AAV plasmids were packaged into AAV-9 capsids with the use of pAdDF6 helper plasmids (providing the three adenoviral helper genes) and pAAV2/9 (providing rep and cap viral genes), obtained from PennVector. Shuttle vectors were generated by direct cloning (GeneScript) of synthesized NheI-SalI fragments into pAcTnT cut with the same restriction enzymes.

The AAV shuttle and helper plasmids were transfected into HEK 293A cells by calcium-phosphate co-precipitation. A total of 840 μg plasmid DNA (mixed in an equimolar ratio) was used per Hyperflask (Corning) seeded with 1.2 × 10^8^ cells the day before. Seventy-two hours after transfection, the cells were collected by centrifugation and the cell pellet was resuspended in TMS (50 mM Tris HCl, 150 mM NaCl, 2 mM MgCl_2_) on ice before digestion with DNase I and RNaseA (0.1 mg ml^−1^ each; Roche) at 37 °C for 60 min. Clarified supernatant containing the viral particles was obtained by iodixanol gradient centrifugation^44^. Gradient fractions containing virus were concentrated using Amicon UltraCel columns (Millipore) and stored at −70 °C.

Standard curves were constructed using known copy numbers (10^5^–10^8^) of the respective plasmid (pAAV-TnT-GFP-Luc, pAAV-TnT-p38γ_act_, pAAV-TnT-p38δ_act_, pAAV-TNT-Flag-DEPTOR and pAAV-TNT-shDeptor) carrying the appropriate complementary DNA.

### *In vivo* protein synthesis assay

For all *in vivo* measurements of protein synthesis, mice were injected intraperitoneally with 0.040 μmol g^−1^ puromycin dissolved in 100 μl PBS. Exactly 30 min after injection, tissues were extracted and frozen in liquid N_2_ for subsequent immunoblot analysis of protein-incorporated puromycin.

### Measurement of *in vitro* protein synthesis by SUnSET assay

All the experiments were performed following the protocol described in ref. 45 with some modifications. MEFs were seeded in six-well plates with 10^6^ cells per well in 1 ml complete DEMEM, and treated with 1 μg ml^−1^ puromycin solution (Sigma) for 10 min at 37 °C and 5% CO_2_ (=pulse). The cells were washed twice with pre-warmed complete medium, resuspended in 1 ml of the same medium and incubated for 50 min at 37 °C and 5% CO_2_ (=chase). The cells were washed twice with cold PBS, and harvested. They were then washed once more by centrifugation at 4 °C in cold PBS/0.1% BSA, and pellets were incubated in 30 μl of staining mix (mouse IgG2 anti-puromycin antibody (12D10) diluted 1/50 in PBS/BSA) for 30 min. The cells were washed twice with PBS/BSA and incubated in 30 μl of PE-labelled anti-mouse IgG (Invitrogen) diluted 1/100 in PBS/BSA. The cells were washed and resuspended in 200 μl PBS/BSA and analysed by FACS (fluorescence-activated cell sorting).

### cDNA transfection-based experiments

Cells were plated at 60% confluence in DMEM/10%FBS 12–15 h before transfection. The cells were transfected using the calcium phosphate method^30^ with the pRK5-based cDNA expression plasmids indicated in the figures. The culture medium was replaced 12 h after transfection with fresh complete medium, and cells were collected 48 h later. For DEPTOR degradation experiments in HEK 293T or HELA cells, the medium was changed after 24 h for medium lacking serum, and after a further 24 h, the cells were incubated in medium with 10% FBS or no FBS together with 10 μM cycloheximide. At the times indicated, the cells were collected and analysed by western blot. To block DEPTOR degradation induced by the constitutionally active p38γ/δ mutants, cells were preincubated with the proteasome inhibitor MG132 (Shelleckchem) at a final concentration of 10 μM.

The plasmids used in the different experiments were pRK5 myc Rat mTOR (plasmid #1861, Addgene); pRK5 FLAG human DEPTOR (plasmid #21334, Addgene); pRK5 FLAG human DEPTOR (13xS/T→A; plasmid #21702, Addgene); pRK5 FLAG DEPTOR (PDZ domain; plasmid #21701, Addgene); pRK5 FLAG DEPTOR (DEP domains; plasmid #21700, Addgene); pcDNA3-myc3-CUL1 (plasmid #19896, Addgene); pcDNA3-myc3-bTrCP (plasmid #20718, Addgene); HA-Ubiquitin (plasmid #18712); pcDNA3 HA human p38γ and pcDNA3 HA human p38δ, kindly provided by Roger Davis (University of Massachusetts Medical School, Worcester, USA); and pCEFL Flag p38γ^D129A^ and pCMV Flag p38δ^F324S^, kindly provided by David Engelberg (The Hebrew University of Jerusalem, Israel).

### *In vitro* kinase assay

One microgram of human GST-DEPTOR protein (H00064798, Novus Biologicals) was incubated for 30 min with 1 μg of active recombinant p38γ, p38γ or p38α kinases (provided by MRC Protein Phosphorylation and Ubiquitylation Unit, Dundee, UK) in the presence of 200 μM cold ATP. The reaction was stopped by adding SDS-containing sample buffer, and proteins were resolved by SDS–PAGE and visualized by staining with colloidal Coomassie Blue. The band containing GST-DEPTOR was excised and treated with DTT to reduce disulfide bonds and with iodoacetamide to derivatize cysteine residues. The protein was in-gel digested with trypsin, and the resulting peptides were extracted from the gel and analysed by nanoscale-microcapillary reversed phase liquid chromatography tandem mass spectrometry^46^,^47^.

### Flow cytometry cell size analysis

MEFs were trypsinized, washed and analysed by FACS to determine cell size based on the mean forward scattered light intensity.

### Blood pressure measurement

Blood pressure in mice was measured using the noninvasive tail-cuff method^48^.

### Statistical analysis

Differences between groups were examined for statistical significance by two-tailed Student’s *t*-test, one-way or two-way analysis of variance coupled to the Bonferroni post test.

The rest of the materials and methods are found in the Supplementary Methods.

## Additional information

**How to cite this article:** González-Terán, B. *et al*. p38γ and δ promote heart hypertrophy by targeting the mTOR-inhibitory protein DEPTOR for degradation. *Nat. Commun.* 7:10477 doi: 10.1038/ncomms10477 (2016).

## Supplementary Material

Supplementary Information Supplementary Figures 1-13, Supplementary Tables 1-2, Supplementary Methods and Supplementary References
