# Supplementary material for: p38γ and δ promote heart hypertrophy by targeting the mTOR-inhibitory protein DEPTOR for degradation
Source: Nat Commun. 2016 Jan 22;7:10477. doi: 10.1038/ncomms10477 (PMC5476828; doi:10.1038/ncomms10477)
Supplement: Supplementary Information — Supplementary Figures 1-13, Supplementary Tables 1-2, Supplementary Methods and Supplementary References [file ncomms10477-s1.pdf]

SUPPLEMENTARY INFORMATION

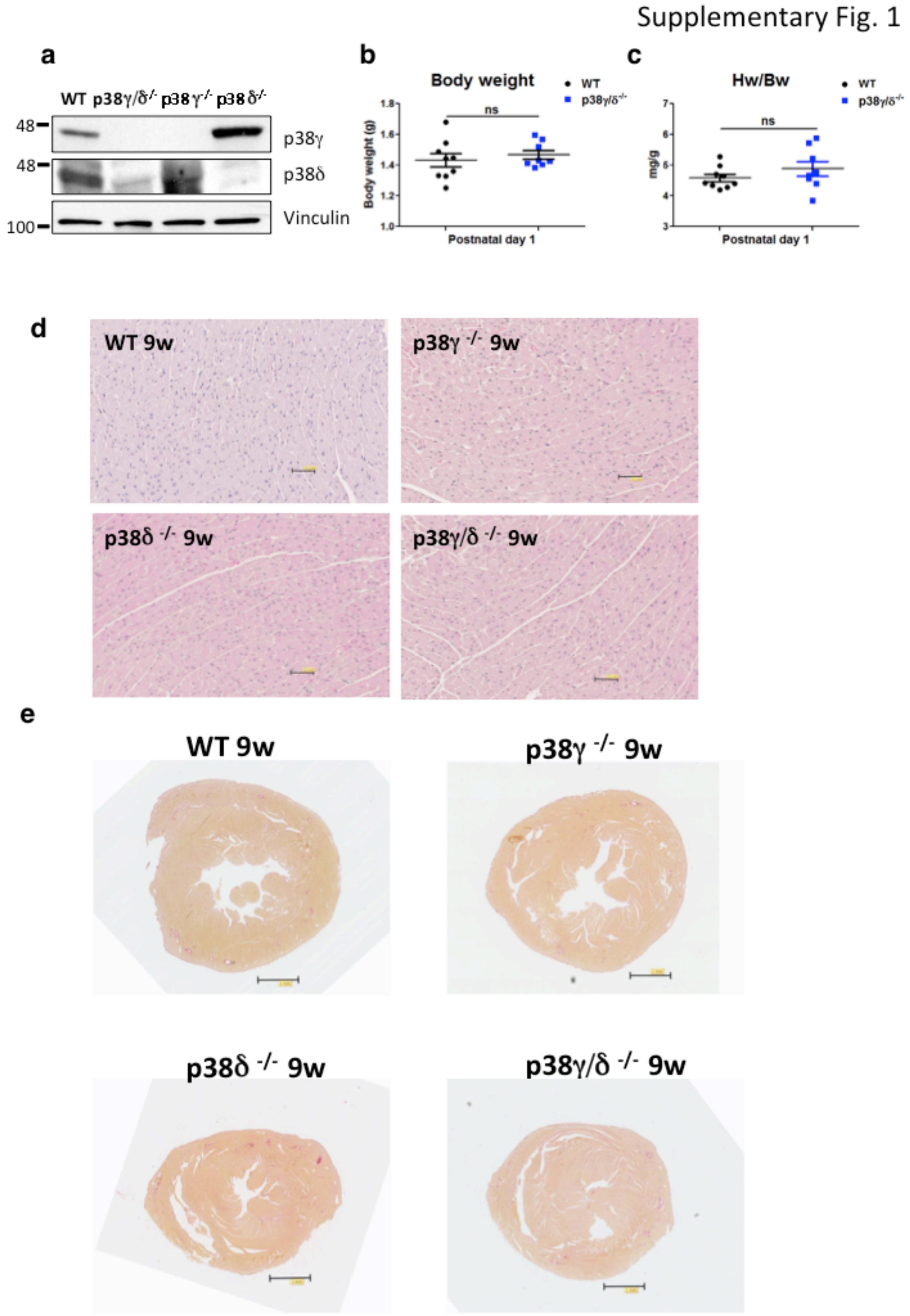

Supplementary Figure 1. p38γ and δ deficiency results in small hearts without fibrosis development.

(a) WT, p38γ/δ$^{-/-}$, p38γ$^{-/-}$, p38δ$^{-/-}$ mice were sacrificed at 9 weeks of age and cardiac p38γ and p38δ expression was analysed in heat lysates.

(b & c) Wild type (WT) and p38γ/δ$^{-/-}$ male mice were sacrificed at postnatal day one and body weight (**b**) and heart weight:tibia length ratio (**c**) were measured (n=8-9). Data are means ± SEM (n=5-13). Ns, P>0.05 (*t*-test).

(d) Representative H&E heart micrographs from 9-week-old WT, p38γ$^{-/-,}$ p38δ$^{-/-}$ and p38γ/δ$^{-/-}$ mice. Scale bar, 50.2μm (n=8-10).

(e) Representative picrosirius red stained transverse heart sections from 9-week-old WT, p38γ$^{-/-}$, p38δ-/- and p38γ/δ-/- mice. Scale bar, 1mm, (n=8-10).

Data are means ± SEM. (*t*-test).

Supplementary Fig. 2

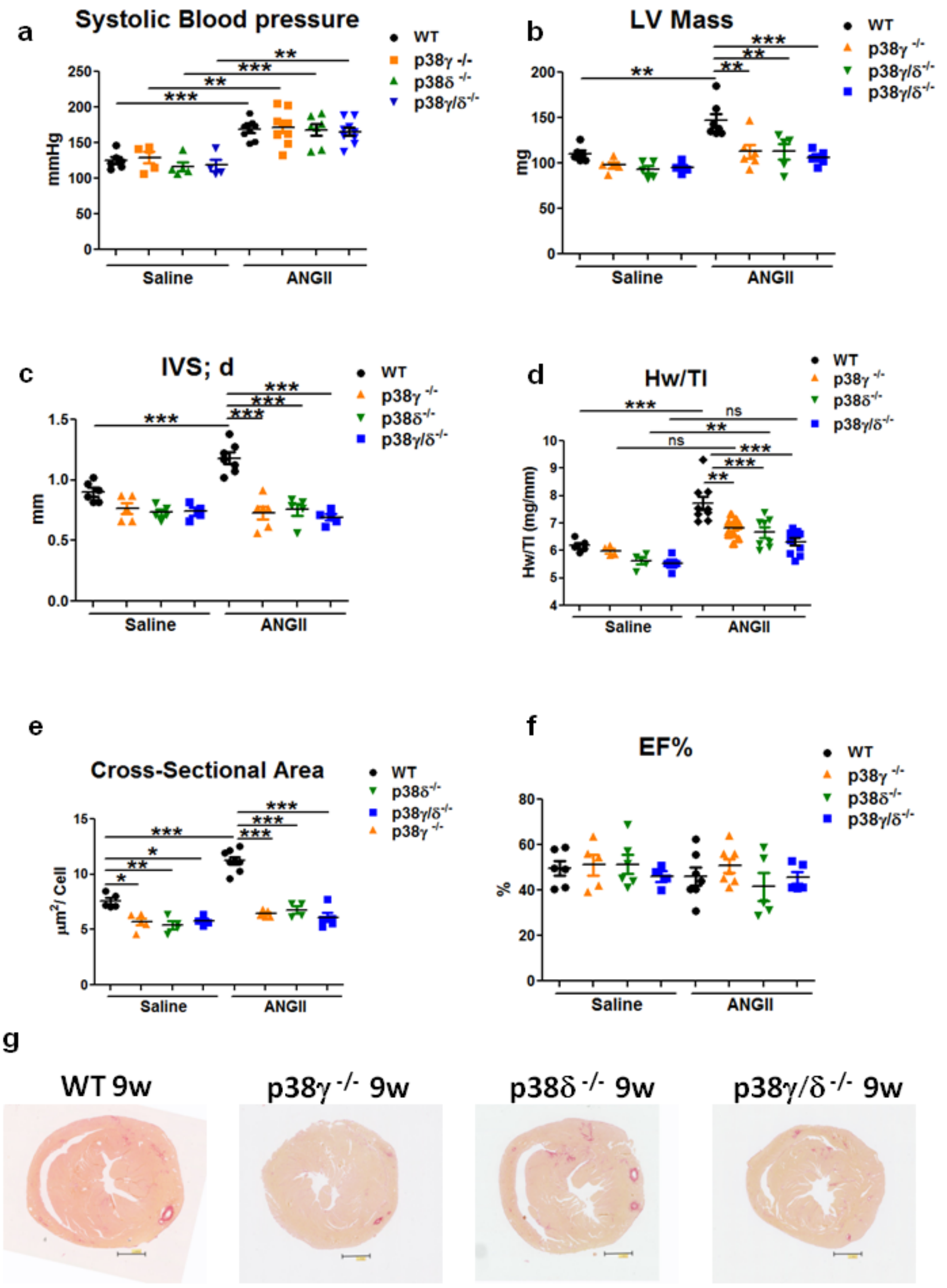

Supplementary Figure 2. p38γ and δ are involved in angiotensin-II -induced hypertrophy in vivo.

WT, $p38\gamma^{-/-}$, $p38\delta^{-/-}$ and $p38\gamma/\delta^{-/-}$ mice were treated for 28 days with angiotensin II (AngII) (1µg/kg/min) or saline, delivered by subcutaneously implanted osmotic minipumps.

(a, b, c & d) $p38\gamma^{-/-}$, $p38\delta^{-/-}$ and $p38\gamma/\delta^{-/-}$ are protected against angiotensin II-induced cardiac hypertrophy. **(a)** WT, $p38\gamma^{-/-}$, $p38\delta^{-/-}$ and $p38\gamma/\delta^{-/-}$ mice treated for 28 days with AngII or Saline show no differences in systolic blood pressure development. **(b & c)** Echocardiography evaluation of LV Mass and IVS;d after AngII or Saline treatment. **(d)** Heart weight to tibia length ratios for WT, $p38\gamma^{-/-}$, $p38\delta^{-/-}$ and $p38\gamma/\delta^{-/-}$ after 28 days of Ang II or saline treatment. (n=4-7). (**e**) Cardiomyocyte cross-sectional area quantified in WGA-stained hearts. **(f)** Echocardiography evaluation of systolic cardiac function after AngII or Saline treatment. IVS;d (inter-ventricular septum in diastole); LV (left ventricle).

(g) WT, $p38\gamma^{-/-}$, $p38\delta^{-/-}$ and $p38\gamma/\delta^{-/-}$ mice treated for 28 days with AngII show no differences in fibrosis development. Representative picrosirius red stained transverse heart sections from 9-week-old WT, $p38\gamma^{-/-}$, $p38\delta^{-/-}$ and $p38\gamma/\delta^{-/-}$ mice treated for 28 days with AngII. Scale bar, 1mm (n=6-12).

Data are means ± SEM. *P<0.05; **P<0.01; ***P<0.001 (1-way ANOVA coupled to Bonferroni's post test).

Supplementary Fig. 3

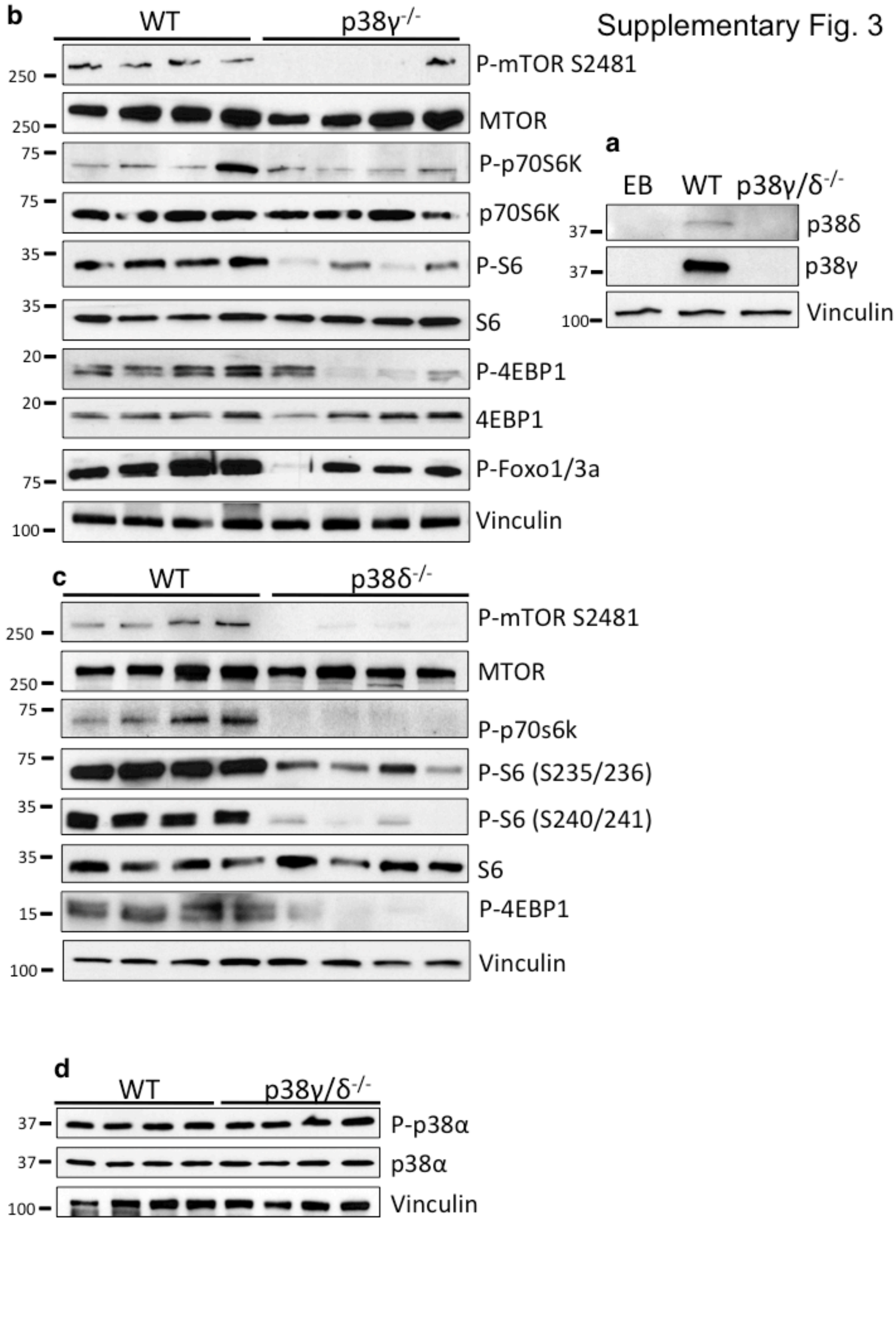

Supplementary Figure 3. Hearts of p38γ$^{-/-}$ or p38δ$^{-/-}$ mice show below-normal mTOR activation.

(**a**) p38γ or p38δ were immunoprecipited from WT and p38γ/δ$^{-/-}$ heart lysates and analyzed by immunoblot. (n=4)

(b & c) WT and p38γ$^{-/-}$ or p38δ$^{-/-}$ heart lysates from 9-week-old male mice were analyzed by immunoblot for mTOR signalling pathway components. (n=4).

(d) WT and p38γ/δ$^{-/-}$ heart lysates from 9-week-old male mice were analyzed by immunoblot for p38 alpha activation and protein levels. (n=4).

Supplementary Fig. 4

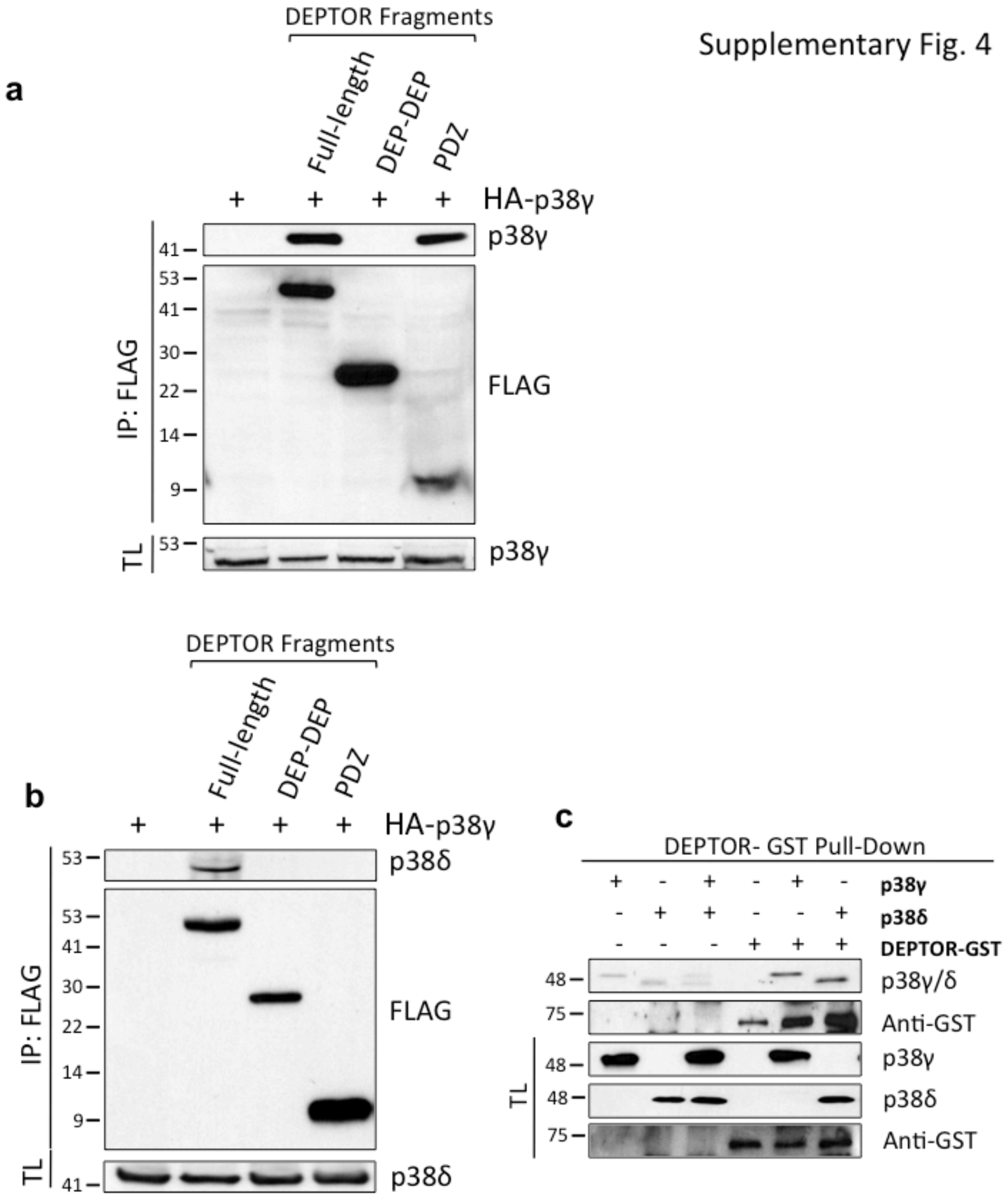

Supplementary Figure 4. p38γ directly interacts with DEPTOR through its PDZ domain.

(a) p38γ interacts with DEPTOR through its PDZ domain**.** Full length Flag-DEPTOR, Flag-DEP DOM and Flag-PDZ DOM were expressed in HEK-293T cells together with HA-p38γ or HA-p38γ alone. Anti-Flag co- immunoprecipitates or total lysates were analyzed by SDS-PAGE.

(b) p38δ interacts with DEPTOR through un unkown domain**.** Full length Flag-DEPTOR, Flag-DEP DOM and Flag-PDZ DOM were expressed in HEK-293T cells together with HA-p38δ or HA-p38δ alone. Anti-Flag co- immunoprecipitates or total lysates were analyzed by SDS-PAGE.

(**c**) p38γ and p38δ directly interact with DEPTOR. GST Pull down experiment. GST-DEPTOR was used to precipitate untagged p38γ and p38δ recombinant proteins.

Supplementary Fig. 5

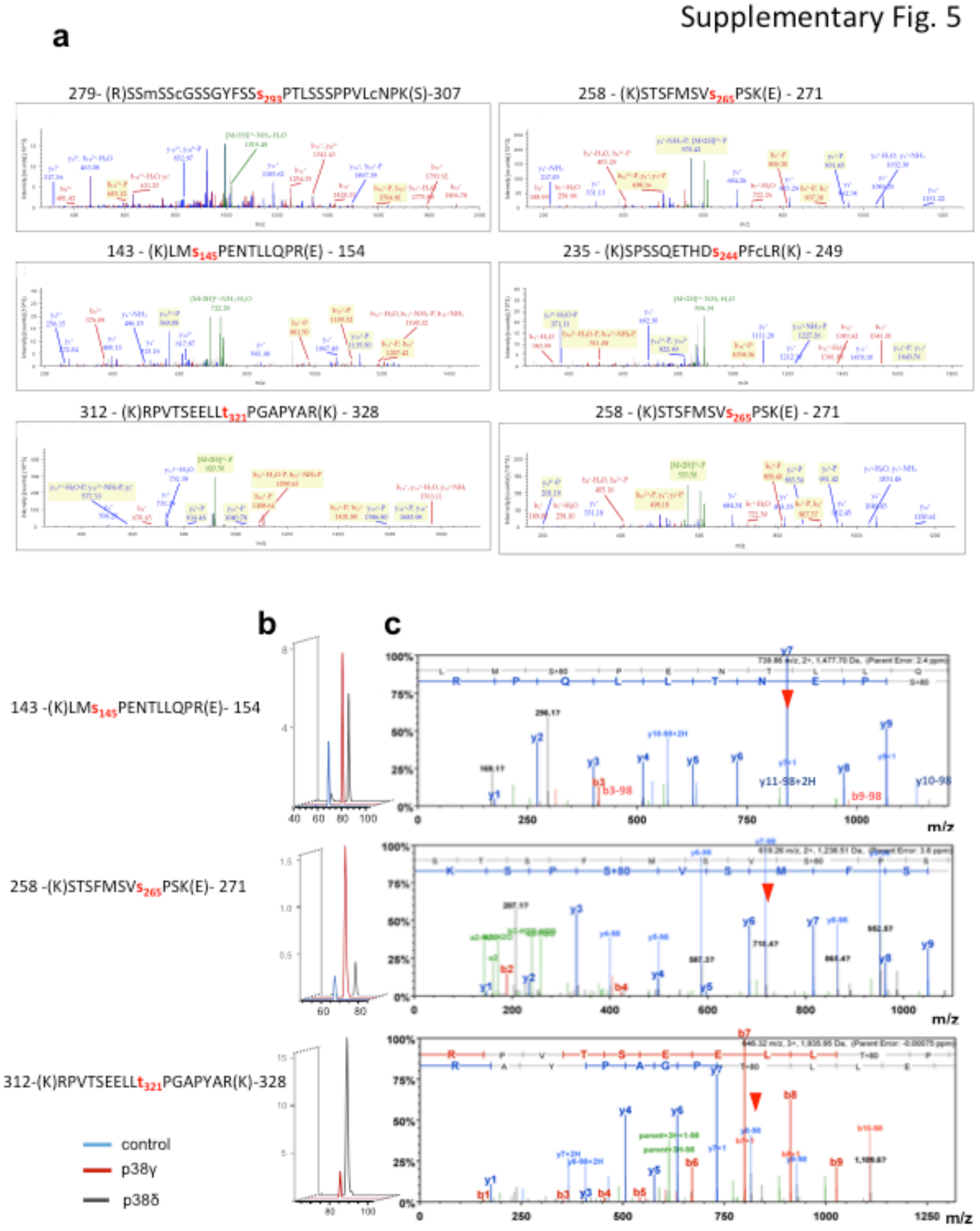

Supplementary Figure 5. DEPTOR is specifically phosphorylated by p38γ and p38δ in vitro and in vivo.

(a) ESI-MS analysis of DEPTOR phosphorylation by constitutively active p38γ or p38δ mutants. In an in vitro kinase assay, recombinant human DEPTOR protein (1μg) was incubated

alone or in the presence of p38γ or p38δ kinases (1μg) and 0.2mM of cold ATP.

(b-c) ESI-MS analysis of DEPTOR phosphorylation by constitutively active p38γ or p38δ mutants. In an in vivo kinase assay, human Flag-DEPTOR was expressed in HEK-293T cells alone or together with constitutively active p38γ or p38δ mutants. HEK-293T cells starved for 30h. DEPTOR phosphorylation was analyzed from Flag immunoprecipitates by mass spectrometry. After trypsin digestion, phosphopeptides previously detected in the *in vitro* experiments and their non-phosphorylated counterparts were monitored using the parallel reaction monitoring (PRM) mode in a Q Exactive hybrid quadrupole-orbitrap mass spectrometer (Thermo Scientific). **(b)** Extracted ion chromatogram traces (XIC) of the fragments indicated with a red triangle in **(c)**. The relative intensity of DEPTOR-derived phosphopeptides in each experiment is expressed as percentage, taking the intensity of the corresponding unmodified peptide as a reference. **(c)** MS/MS spectra interpreted using Scaffold at the peak apex of each phosphorylated peptide. Note that the MS/MS spectra confirm peptide phosphorylation at Ser and Thr residues in the consensus sequences SP and TP, respectively. For peptide (K)STSFMSVsPSK(E), treatment with MG132 was necessary to prevent their proteolysis.

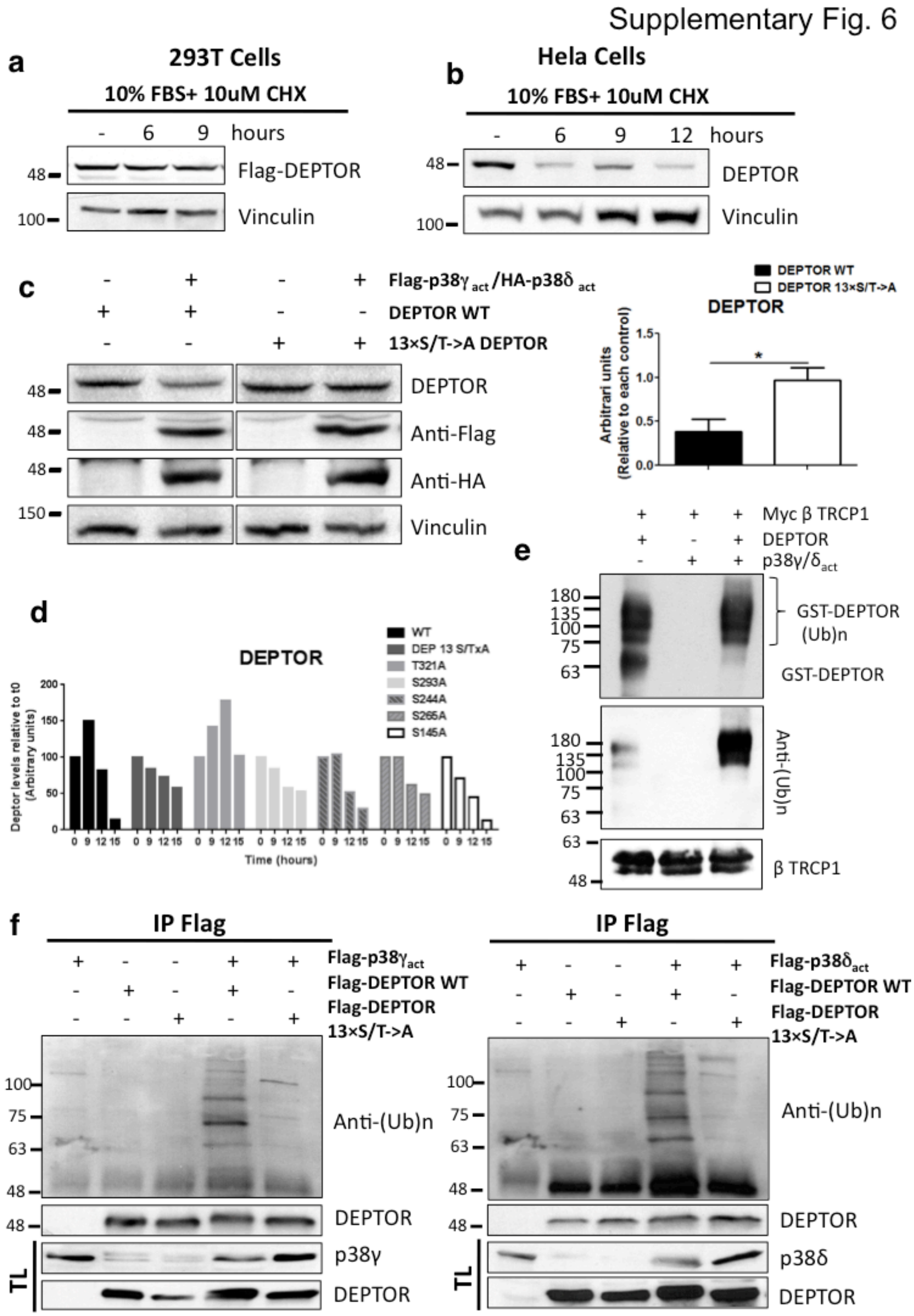

Supplementary Figure 6. DEPTOR degradation is dependent of p38γ and p38δ phosphorylation.

(a) Flag-DEPTOR was expressed in HEK-293T cells, which were serum starved for 30 hr. Fresh serum-free or 10% FBS medium was then added together with 10μM cycloheximide (CHX) for the times indicated. Flag-DEPTOR levels were analyzed by immunoblotting with the indicated antibodies.

(b) HeLa cells were serum starved for 30 hr and fresh serum-free or 10% FBS medium was then added together with 10μM cycloheximide (CHX) for the times indicated. Endogenous DEPTOR levels were then analyzed by immunoblotting with the indicated antibodies.

(c) Constitutively active p38γ and p38δ mutants induce DEPTOR degradation but not 13xS/T→A DEPTOR mutant degradation. Blots were quantified with ImageJ relative to vinculin levels. Data are means ± SEM. *P<0.05 (*t*-test).

(d) Quantification of DEPTOR protein levels from immunoblot assay. DEPTOR phosphorylation mutants were expressed in HEK-293T alone or together with constitutively active p38γ and p38δ mutants. Fresh media without serum was added together with 10μM cycloheximide (CHX) and cells harvested at the times indicated. DEPTOR degradation was analyzed by immunoblot.

(e) SCF E3 was prepared by FLAG beads IP using 293 cells transfected with βTrCP. Recombinant GST-DEPTOR was in vitro phosphorylated or not with active p38γ and p38δ. SCF E3, and DEPTOR as the substrate, were added into a reaction mixture containing ATP, ubiquitin, E1 and E2, followed by constant mixing for 60 min. The reaction mixture was then loaded onto PAGE gel for IB with the indicated antibodies.

(f) Constitutively active p38γ and p38δ mutants induce ubiquitination of DEPTOR but not the 13xS/T→A DEPTOR mutant. Flag-DEPTOR or Flag- 13xS/T→A DEPTOR was expressed in HEK-293T cells alone or together with constitutively active p38γ and p38δ mutants. HEK-293T cells starved for 30 hr and were incubated for 12 hr with 10μM MG132. DEPTOR was inmunoprecipitated using anti-Flag antibody. Inmunoprecipitates and cell lysates were analyzed by immunoblotting with the indicated antibodies.

Data are means ± SEM (n=3). *P<0.05 (*t*-test).

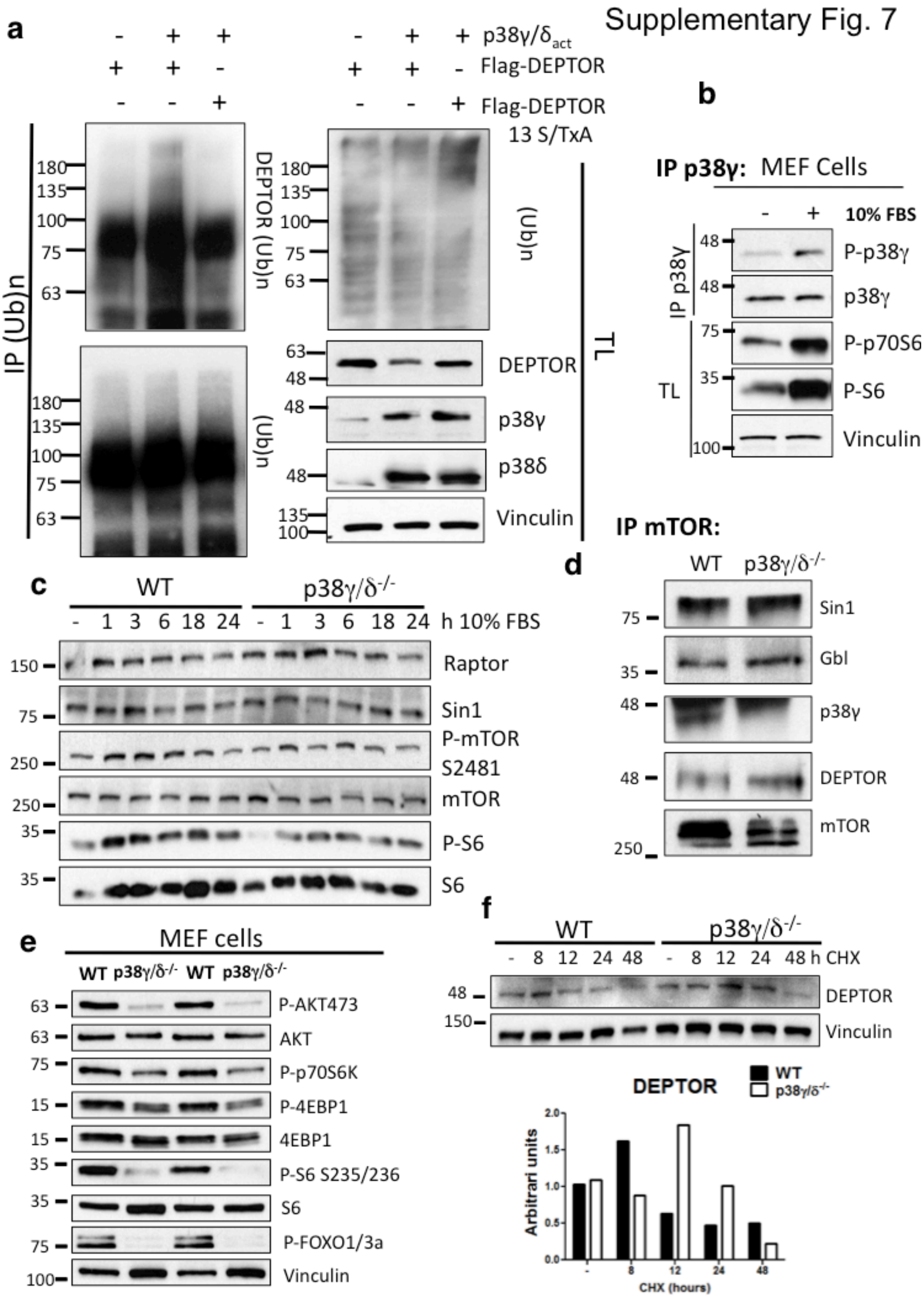

Supplementary Figure 7. p38γ and p38δ control mTOR pathway through the regulation of DEPTOR degradation.

(a) Constitutively active p38γ and p38δ mutants induce ubiquitination of DEPTOR but not the 13xS/T→A DEPTOR mutant. Flag-DEPTOR or Flag- 13xS/T→A DEPTOR was expressed in

HEK-293T cells alone or together with constitutively active p38γ and p38δ mutants and HA-Ub. Cells were serum starved and incubated for 12 hr with 10μM MG132. Poly-Ubiquitinated proteins were IP from cell lysates with bead-conjugated anti-HA Ab and immunoprecipitates were analyzed by immunoblotting with the indicated antibodies.

(b) p38γ is activated upon serum stimulation. MEF cells were stimulated with 10% FBS during 30 min. Then cells were harvested and p38γ immunoprecipitated. Total lysates (TL) and p38γ immunoprecipitates analyzed by immunoblot.

(c) p38γ/$\delta^{-/-}$ MEFs have below-normal serum-induced mTOR activation. WT and p38γ/$\delta^{-/-}$ MEFs were serum-starved for 30 hr, followed by serum addition. Cells were harvested at the indicated time points for immunoblotting with the indicated antibodies.

(d) p38γ/$\delta^{-/-}$ MEFs have below-normal activation of the mTOR pathway in starvation conditions. Confluent WT and p38γ/$\delta^{-/-}$ MEFs were serum-starved for 16 hr and harvested for immunoblotting with the indicated antibodies.

(e) Higher amounts of endogenous DEPTOR co-immunoprecipitate with mTOR in p38γ/$\delta^{-/-}$ MEFs. Endogenous mTOR was immunoprecipitated from WT and p38γ/$\delta^{-/-}$ MEFs and immunoprecipitates were analyzed by SDS-PAGE.

(f) DEPTOR degradation is impaired in p38γ/$\delta^{-/-}$ MEF cells. WT and p38γ/$\delta^{-/-}$ MEFs were treated with 10μM cycloheximide (CHX), harvested at the times indicated, and endogenous DEPTOR levels were analyzed by immunoblotting. Western blot was quantified.

Supplementary Fig. 8

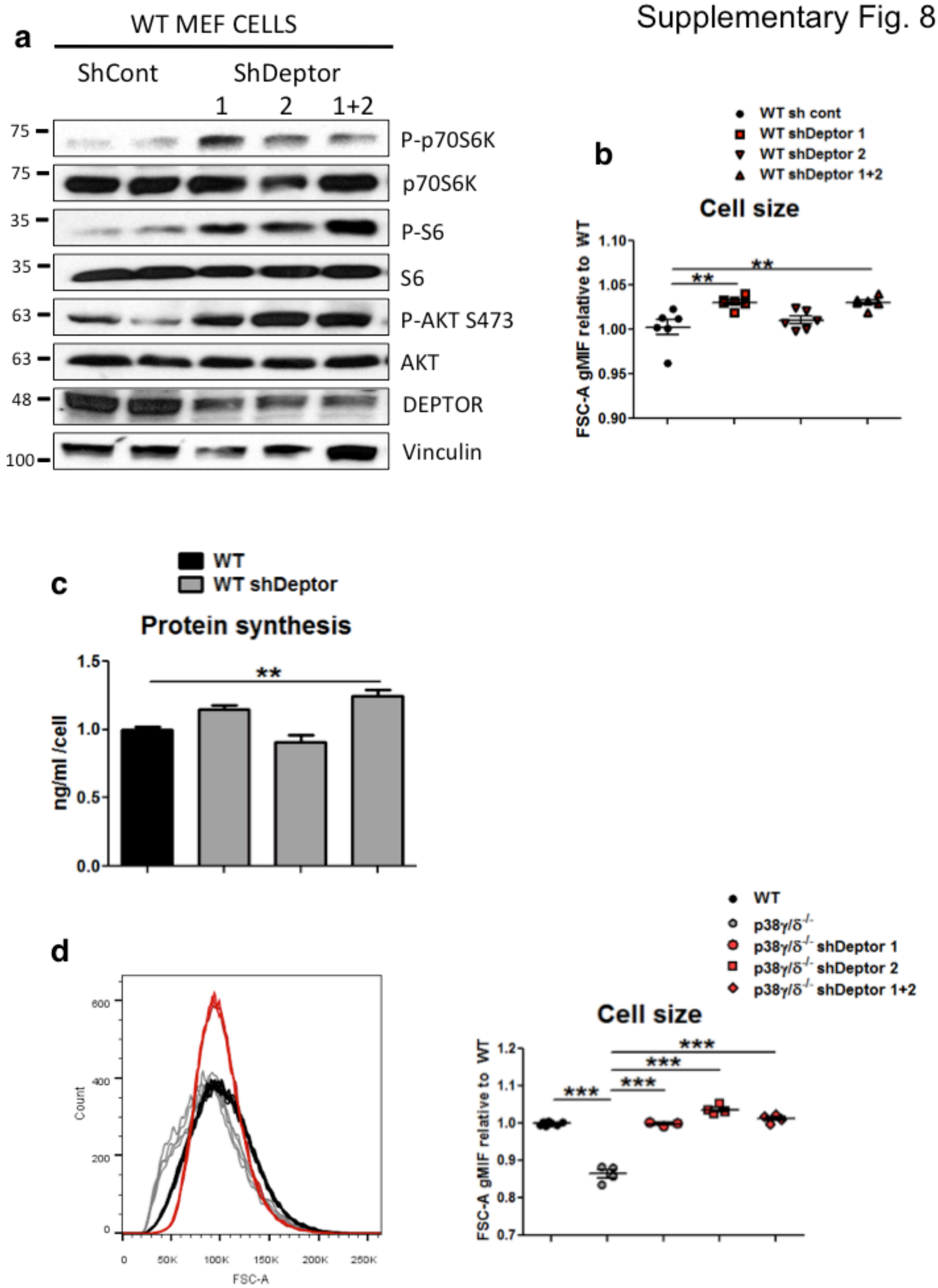

Supplementary Figure 8. Deptor silencing leads to mTOR pathway hyperactivation and

reverts p38γ/δ$^{-/-}$ reduced cell size.

(a, b & c) Silencing DEPTOR in WT MEFs results in mTOR hyperactivation and a slight increase in protein syinthesis. MEFs were singly or doubly infected with two different DEPTOR lentiviral shRNA constructs for 24 hr. Uninfected cells were eliminated by selection with 3 µg/ml puromycin for 1 week. (**a**) The resulting cell lines were then serum-starved for 24 hr before harvesting. Equal amounts of whole cell lysates were immunoblotted with the indicated antibodies. (**c**) Cell size was measured by flow cytometry (forward scatter). *Left*: Representative histogram. *Right*: Quantification graph of the forward scatter mean fluourescence intensity (FSC-A MFI) relative to WT. (**c**) In the resulting cell lines the protein concentration per cell was measured by Bradford method. (n=4-6)

(d) Silencing DEPTOR in p38γ/δ$^{-/-}$ MEFs rescues normal cell size. MEFs were infected with two DEPTOR lentiviral shRNA constructs alone or in combination for 24 hr. Uninfected cells were eliminated by selection with 3 µg/ml puromycin for 1 week. In the resulting cell lines cell size was measured by flow cytometry (forward scatter). *Top*: Representative histogram. *Bottom*: Quantification of forward scatter mean fluorescence intensity (FSC-A MIF) relative to WT cells. (n=4)

Data are means ± SEM (n=6). **P<0.01; ***P<0.001 (1-way ANOVA coupled to Bonferroni's post test).

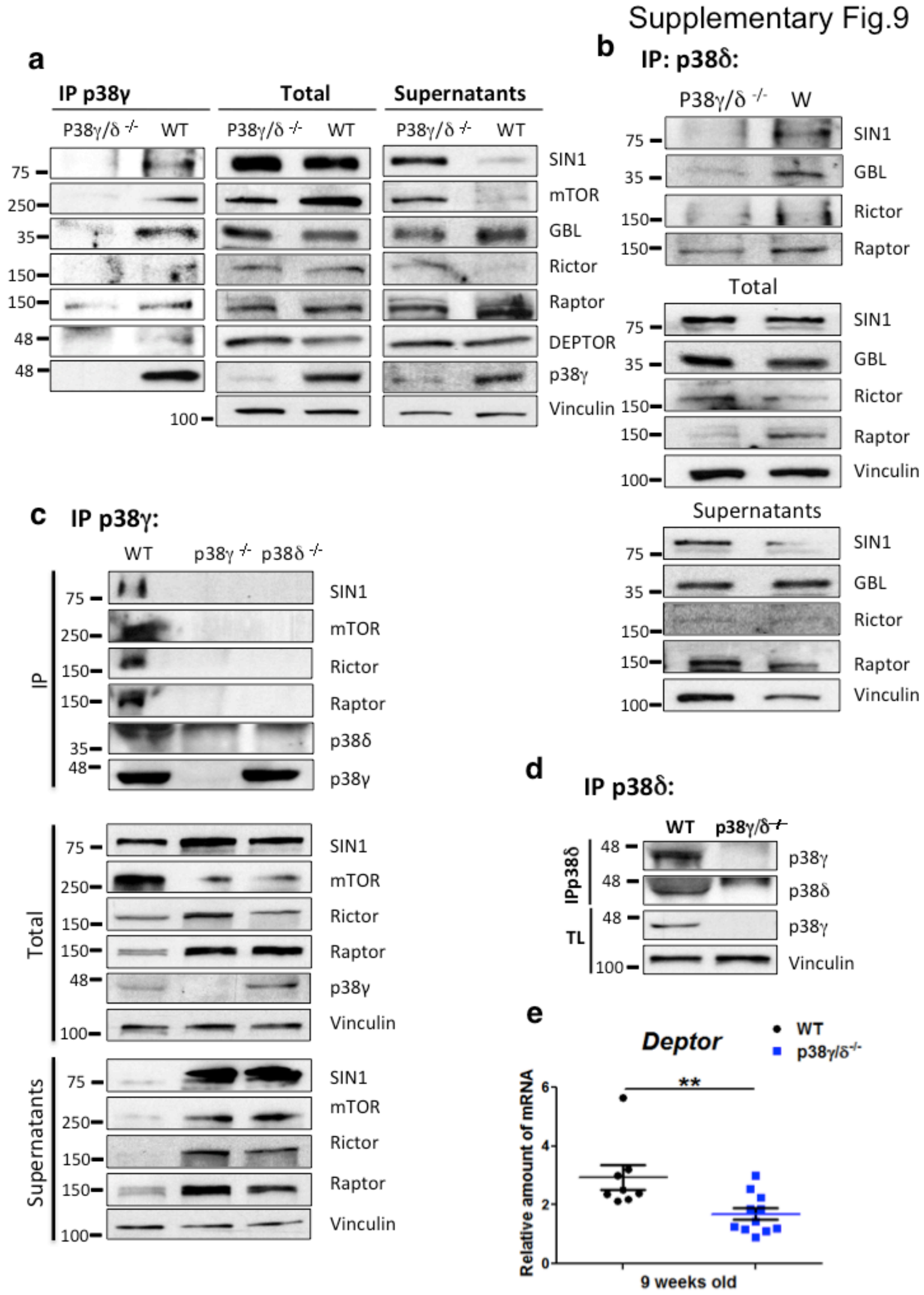

Supplementary Figure 9. p38γ and δ are mutually necessary for their interaction with mTOR complexes in heart tissue.

(a) Endogenous cardiac mTOR, GβL, Raptor, Rictor and Sin-1 co-immunoprecipitate with endogenous p38γ. p38γ immunoprecipitates (IP), total lysates (Total) and supernatants from WT and p38γ/$\delta^{-/-}$ heart lysates were analyzed by SDS-PAGE.

(b) Endogenous cardiac mTOR, GβL, Raptor, Rictor and Sin-1 co-immunoprecipitate with endogenous p38δ. p38δ immunoprecipitates (IP), total lysates and Co-IP supernatants from WT and p38γ/$\delta^{-/-}$ heart lysates were analyzed by SDS-PAGE.

(c) Endogenous cardiac mTOR, Raptor, Rictor, Sin-1 and p38δ co-immunoprecipitate with endogenous p38γ only when both p38γ and p38δ are present. p38γ immunoprecipitates (IP), total lysates and Co-IP supernatants from WT, p38$\gamma^{-/-}$ and p38$\delta^{-/-}$ heart lysates were analyzed by SDS-PAGE.

(d) Endogenous p38γ co-immunoprecipitates with endogenous p38δ in heart lysates. p38δ immunoprecipitates (IP) and total lysates (TL) from WT and p38γ/δ-/- heart lysates were analyzed by SDS-PAGE.

(e) p38γ/$\delta^{-/-}$ hearts present reduced DEPTOR mRNA levels. DEPTOR mRNA was measured by qRT-PCR and normalized to GADPH mRNA levels. (n=7-11).

Data are means ± SEM (n=7-10). **P<0.01 (*t*-test).

Supplementary Fig.10

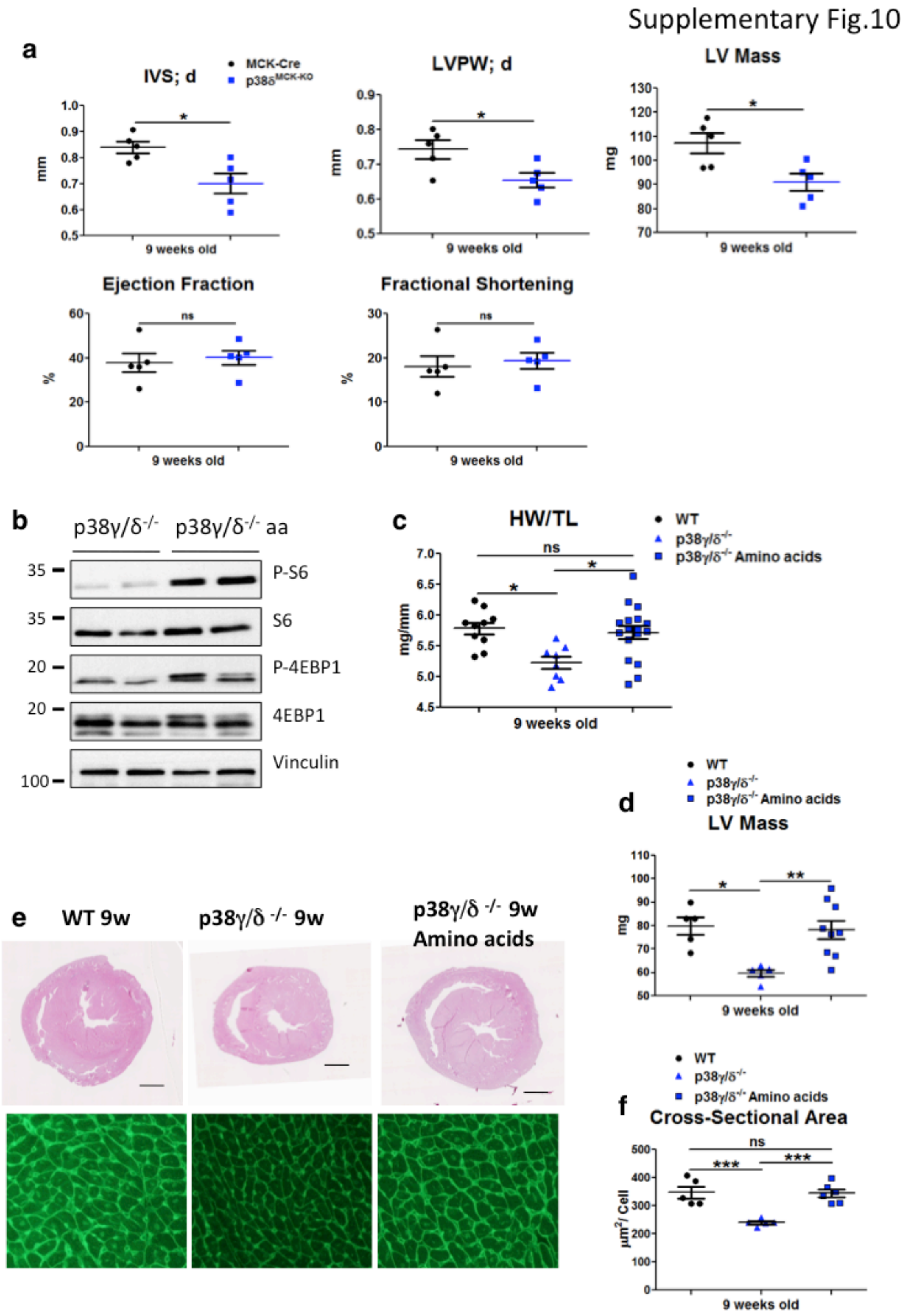

Supplementary Figure 10. Amino acid induced mTOR activation in mice deficient in p38γ and δ is sufficient to revert its small heart phenotype.

(a) Echocardiography results for 9-week-old MCK-Cre and MCK p38δ KO (p38δ$^{MCK\text{-}KO}$) mice. IVS;d (inter-ventricular septum in diastole); LVPW;d (left ventricle posterior wall in diastole); LV (left ventricle). (n=5).

(b, c, d & e) Amino acids (aa) treatment reverts reduced heart size in $p38\gamma/\delta^{-/-}$ mice. WT and $p38\gamma/\delta^{-/-}$ mice were injected daily with a mixture of amino acids i.p or Saline until 9 weeks of age. **(b)** Amino acid treatment activated mTOR pathway in $p38\gamma/\delta^{-/-}$ 9 weeks old hearts. Heart lysates were analyzed by immunoblot for mTOR pathway activation. **(c)** Heart weight to tibia length ratio. **(g)** Echocardiography analysis LV Mass at 9 weeks of age in saline or amino acid treated mice. **(e)** *Top*: Representative H&E stained transverse heart sections from 9-week-old WT and $p38\gamma/\delta^{-/-}$ mice after amino acid or saline treatment. Scale bar, 1mm. *Bottom*: Representative FITC-WGA staining (green) in hearts from 9-week-old 9-week-old WT and $p38\gamma/\delta^{-/-}$ mice after amino acid or saline treatment. **(f)** Cardiomyocyte cross-sectional area quantification from the FITC-WGA staining (green) in hearts from 9-week-old

Data are means ± SEM (n=5). *P<0.05; **P<0.01; ***P<0.001 (2-way ANOVA coupled to Bonferroni's post test or *t*-test).

Supplementary Fig. 11

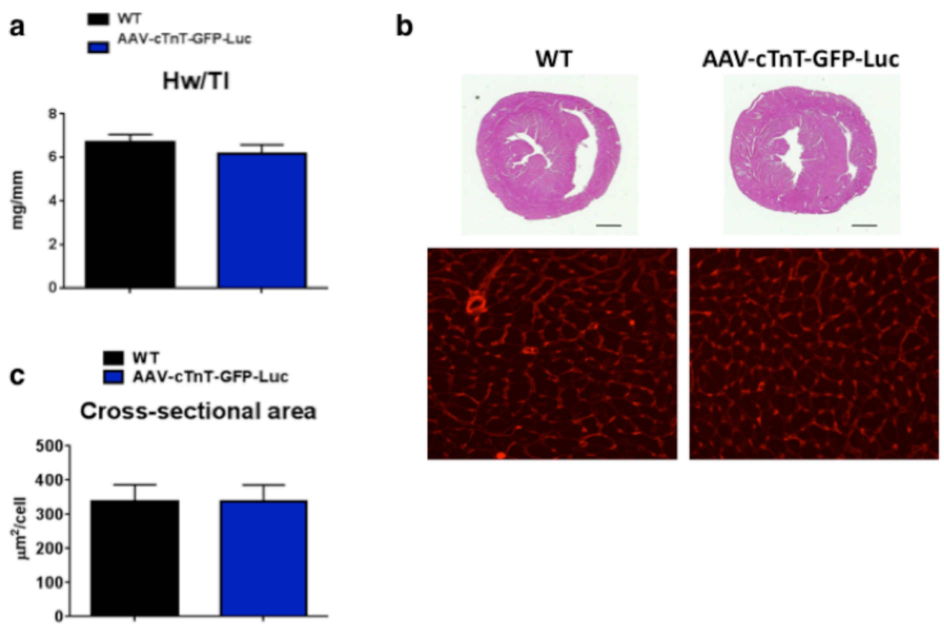

Supplementary Figure 11. Adeno-associated control virus injection does not affect cardiac growth.

(a, b & c) Adeno-associated serotype 9-virus control injection does not affect cardiac growth. WT mice were injected or not at 4 weeks old with AAV-cTnT-GFP-Luc virus and heart phenotype analyzed at 9 weeks of age. **(a)** Heart weight to tibia length ratio. **(b)** *Top*: Representative H&E stained transverse heart sections from WT and AAV-cTnT-GFP-Luc injected mice. Scale bar, 1mm. *Bottom*: Representative WGA (red) and dapi (blue) staining in hearts from WT and AAV-cTnT-GFP-Luc injected mice. **(c)** Cardiomyocyte cross-sectional area quantification from the WGA staining (red) in heart sections.

Data are means ± SEM (n=5), (*t*-test).

Supplementary Fig. 12

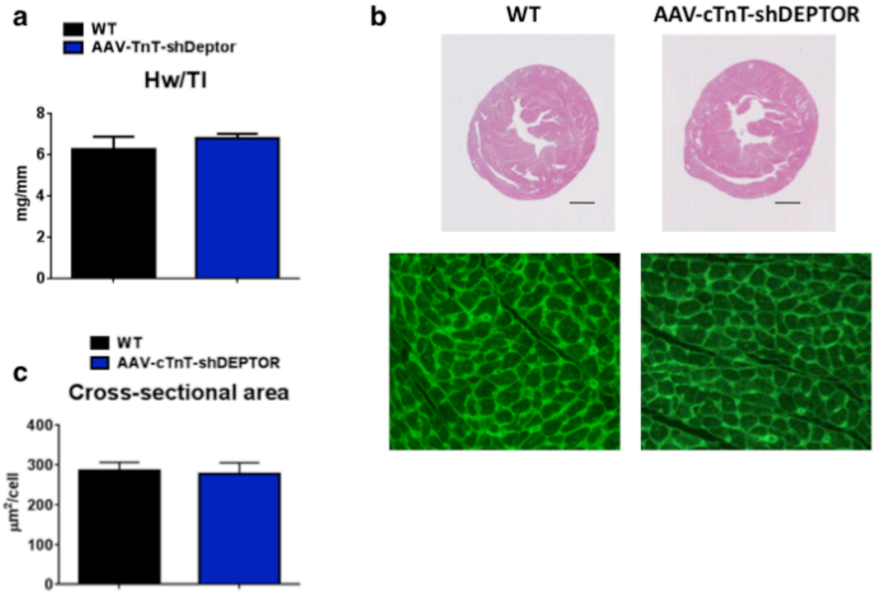

—

Supplementary Figure 12. Adeno-associated shDEPTOR virus injection in WT mice does not affect cardiac growth.

(a, b & c) Adeno-associated serotype 9-virus shDEPTOR was injected to 4 weeks-old mice and heart phenotype analyzed at 8 weeks-old. **(a)** Heart weight to tibia length ratio. **(b)** *Top*: Representative H&E stained transverse heart sections from 8-week-old WT and AAV-cTnT-shDEPTOR injected mice. Scale bar, 1mm. *Bottom*: Representative FITC-WGA staining (green) in hearts from 8-weeks-old WT and AAV-cTnT-shDEPTOR injected mice. **(c)** Cardiomyocyte cross-sectional area quantification from the FITC-WGA staining (green) in hearts from 8-week-old.

Data are means ± SEM (n=5), (*t*-test).

Supplementary Figure 13. Western blots full scans.

Figure 1A

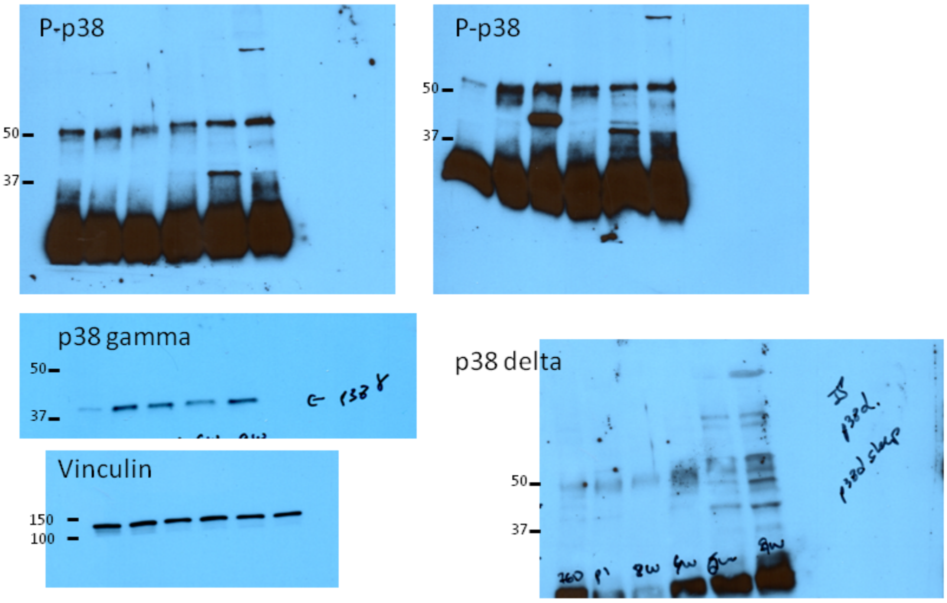
P-p38
50
37
P-p38
50
37
p38 gamma
50
37
p38 delta
50
37
Vinculin
150
100

Figure 2A

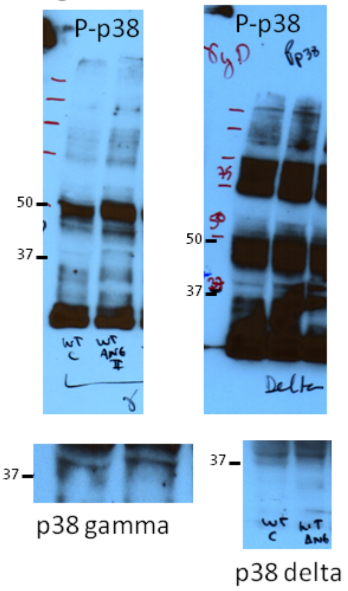

Figure 3A

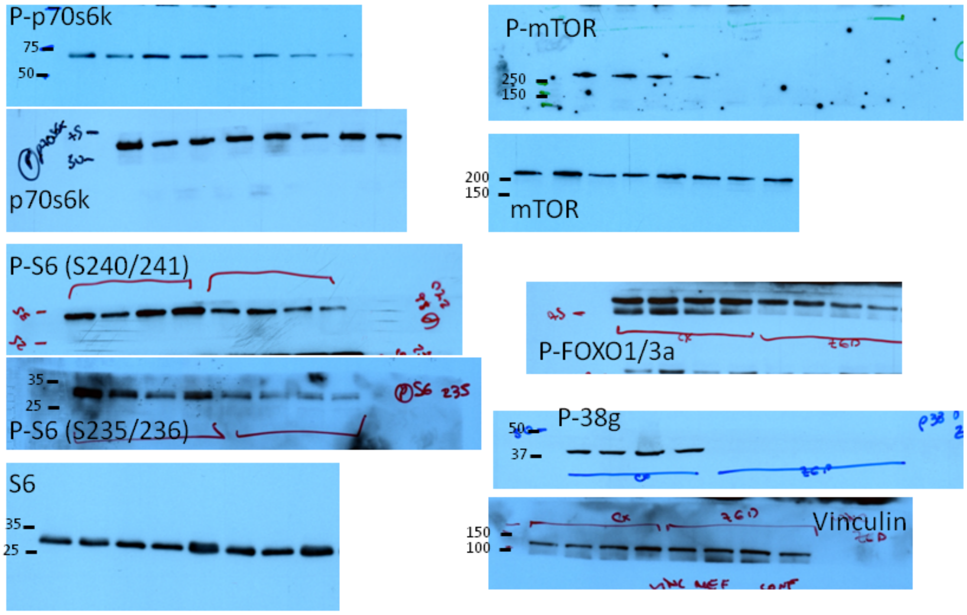

Figure 3B

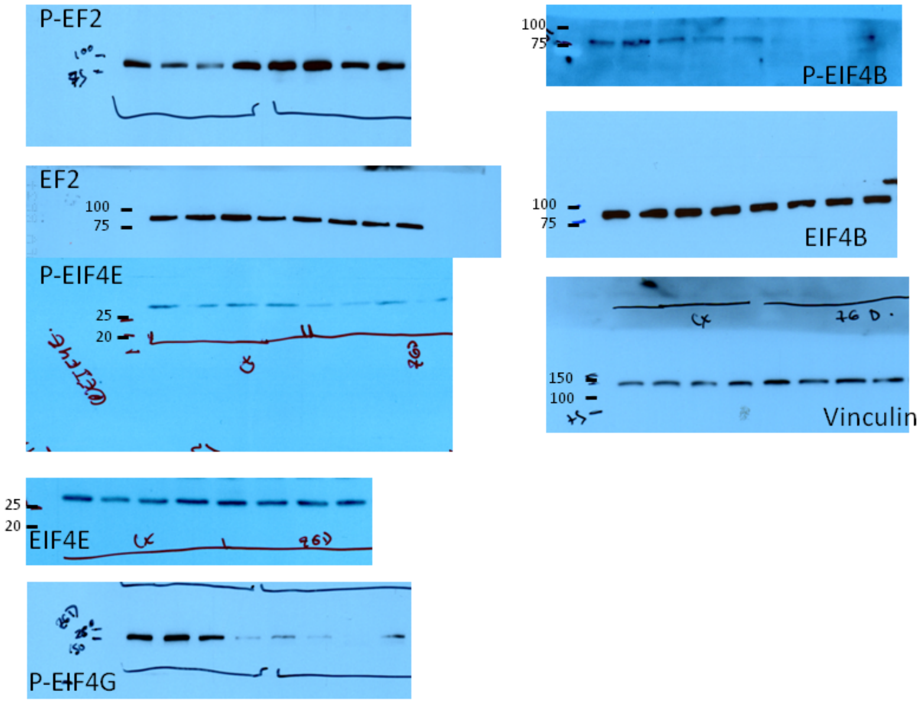

Figure 3C

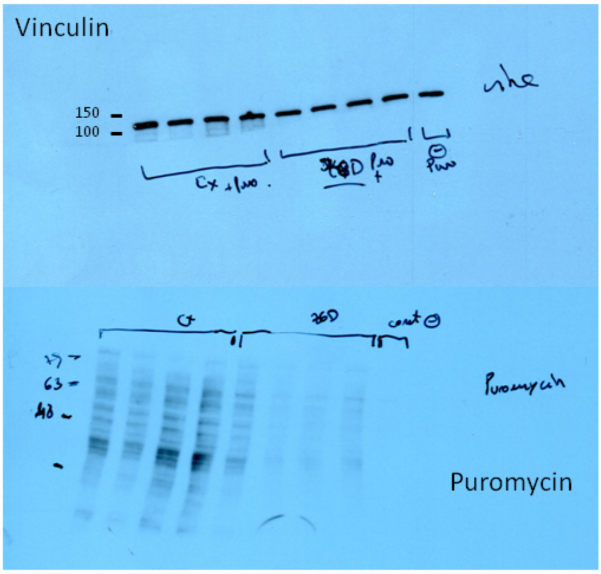

Figure 4A

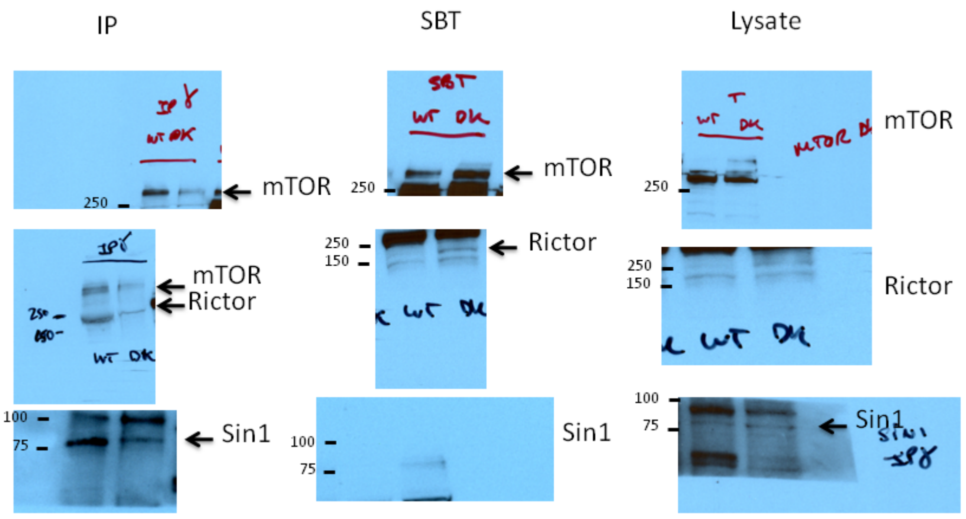

Figure 4A

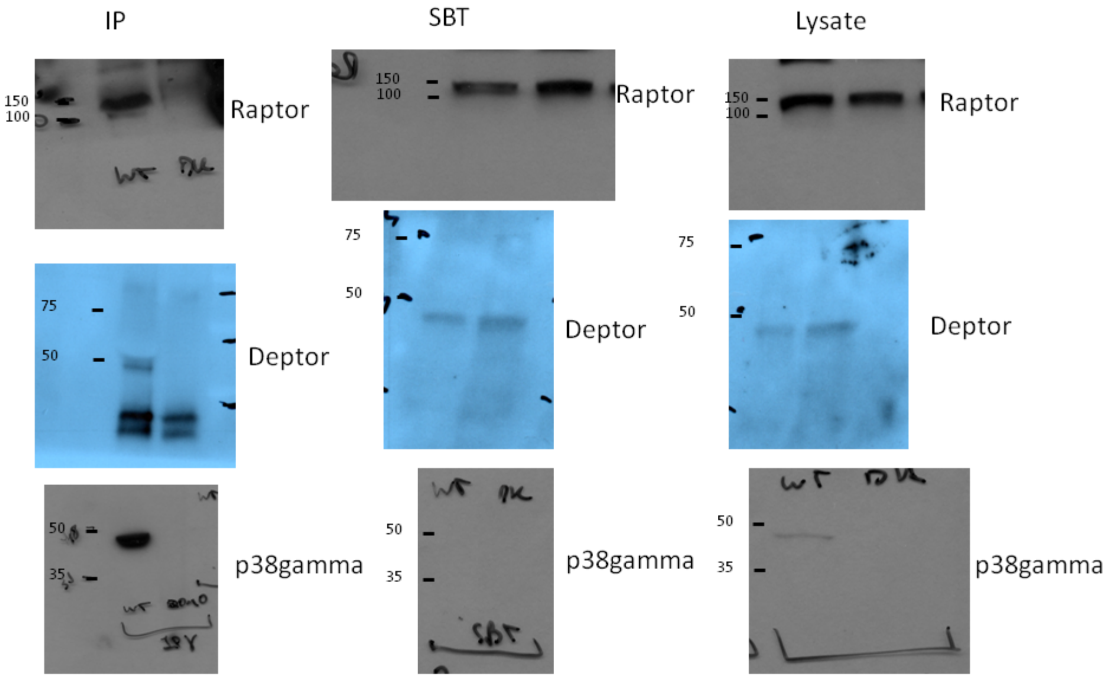

Figure 4B

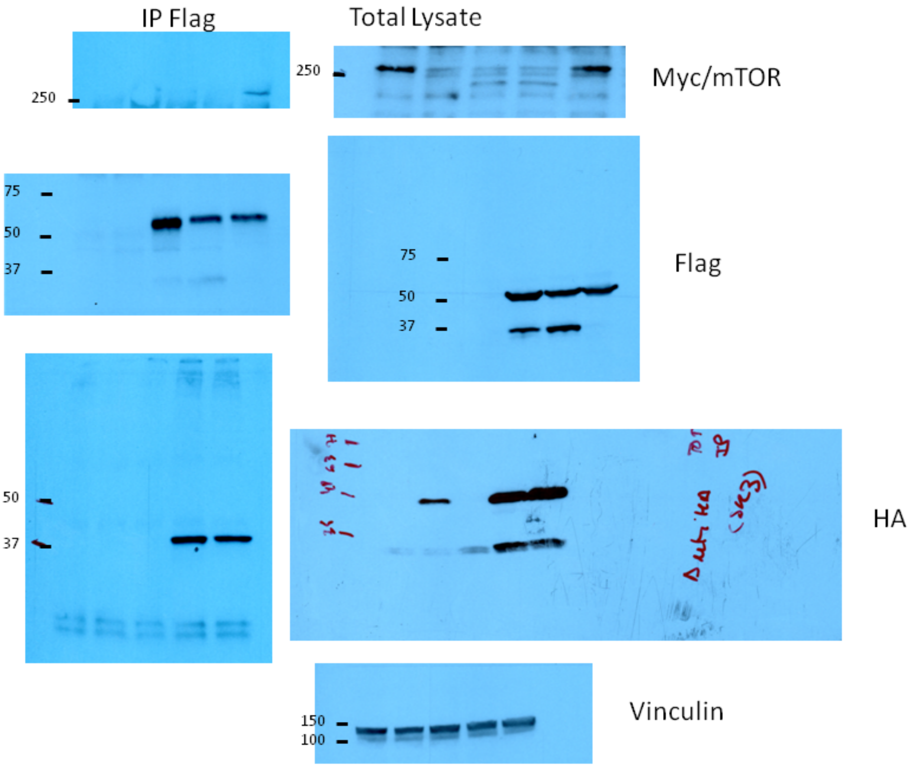

Figure 4C

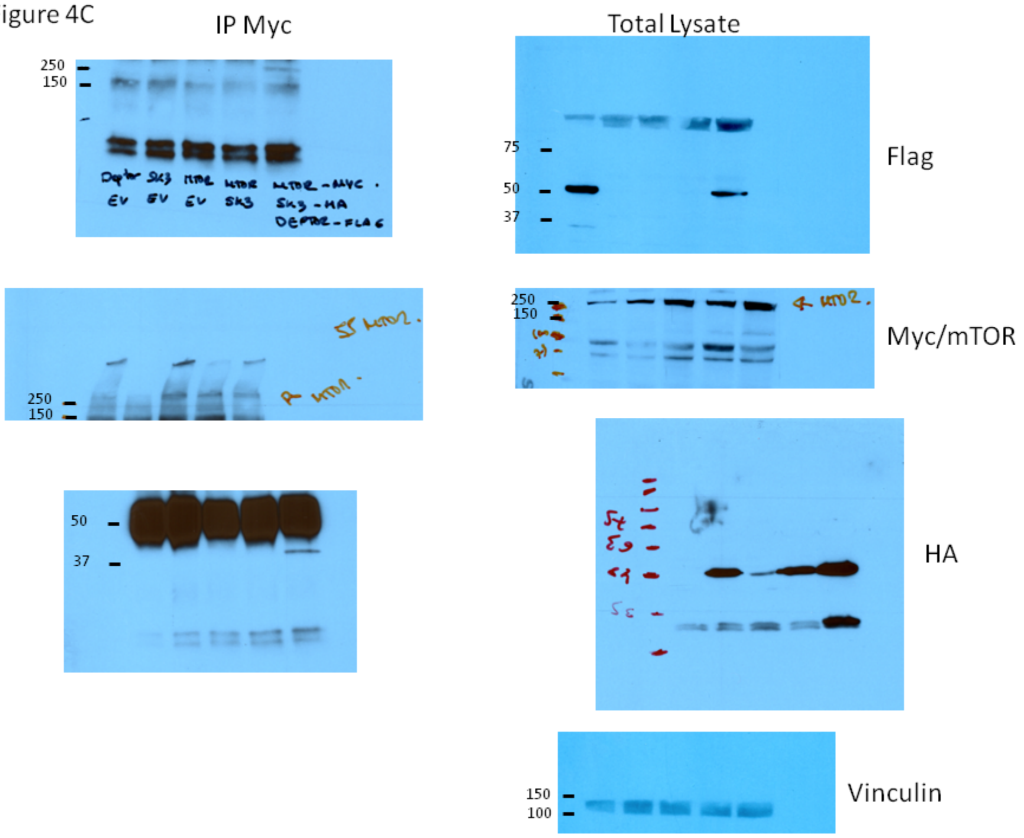

Figure 4D

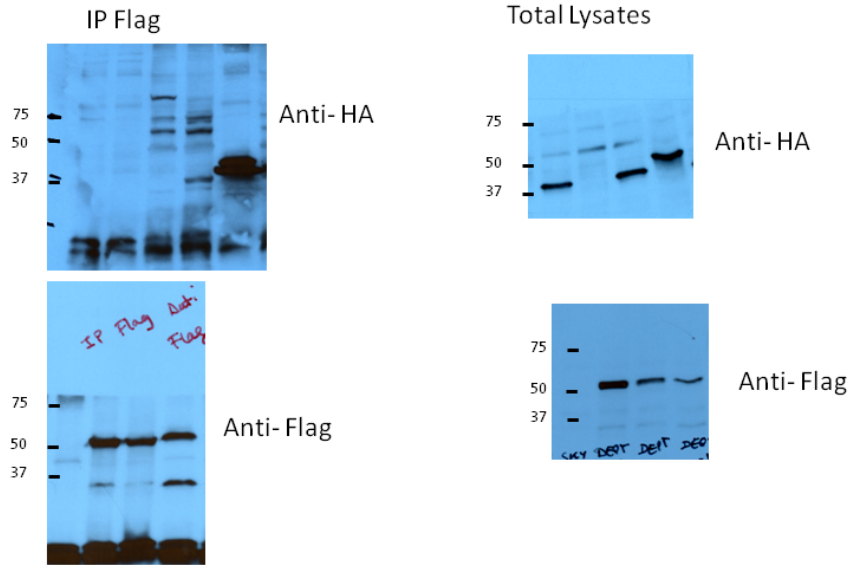

Figure 4E

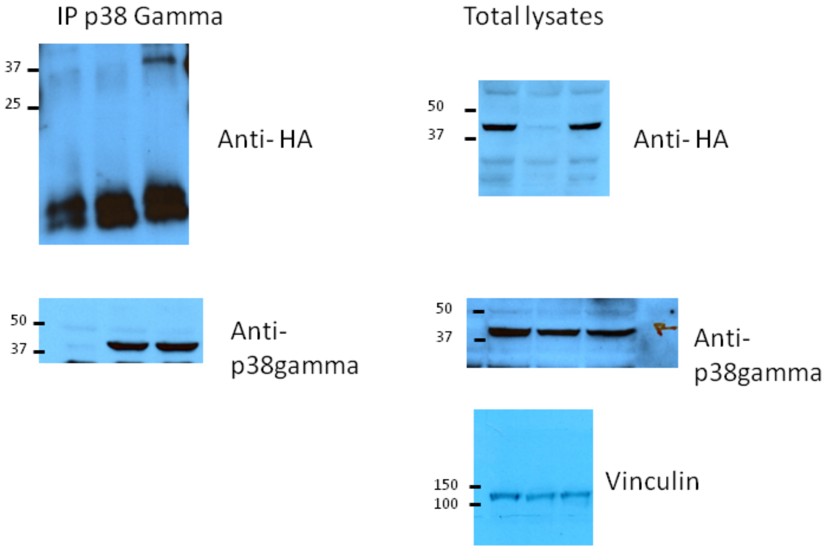

Figure 5B

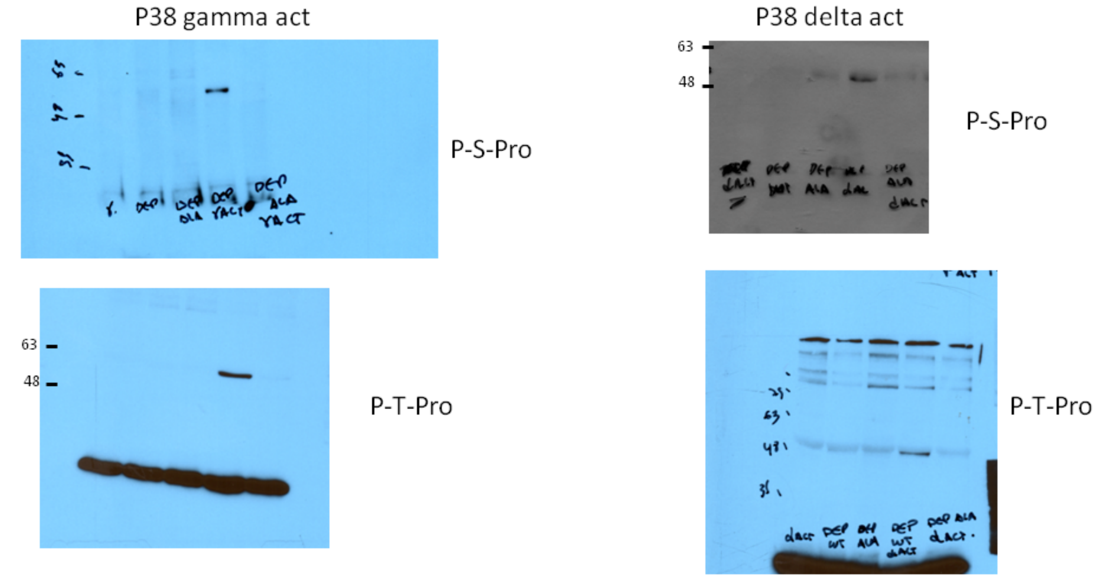

Figure 5B

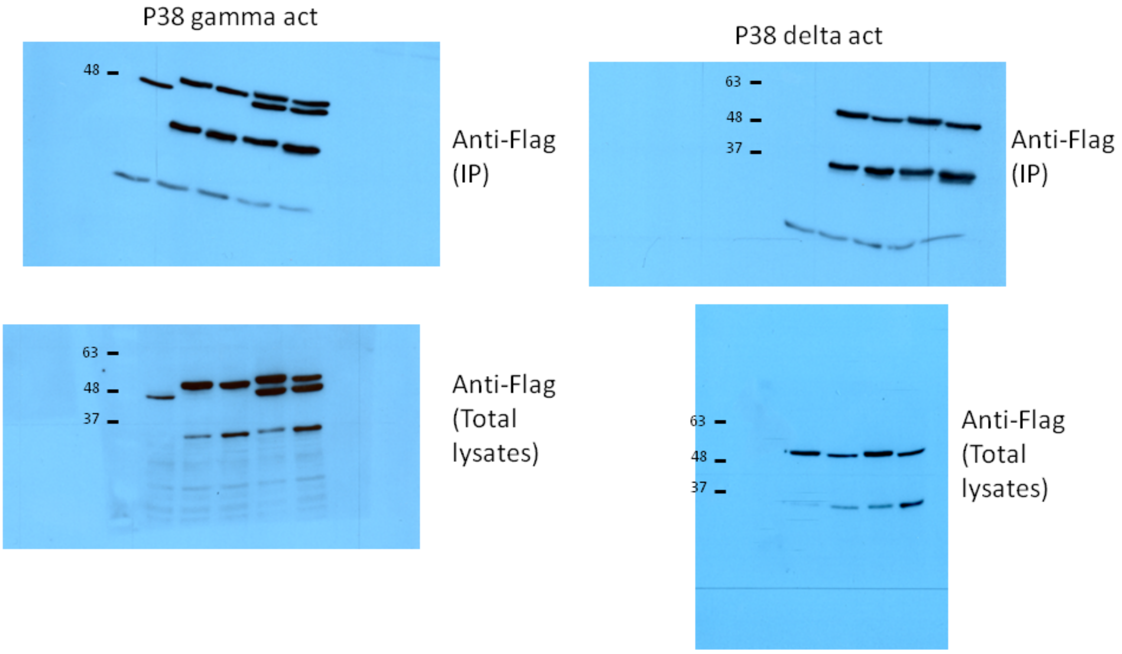

Figure 5C

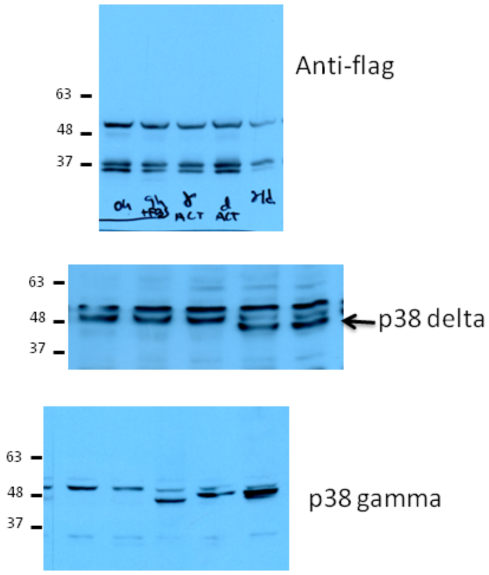

Figure 5D

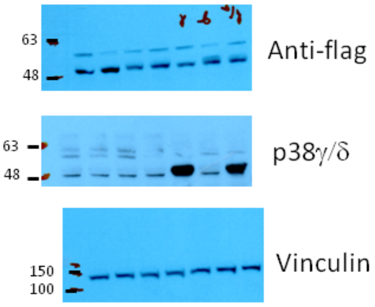

Figure 5E

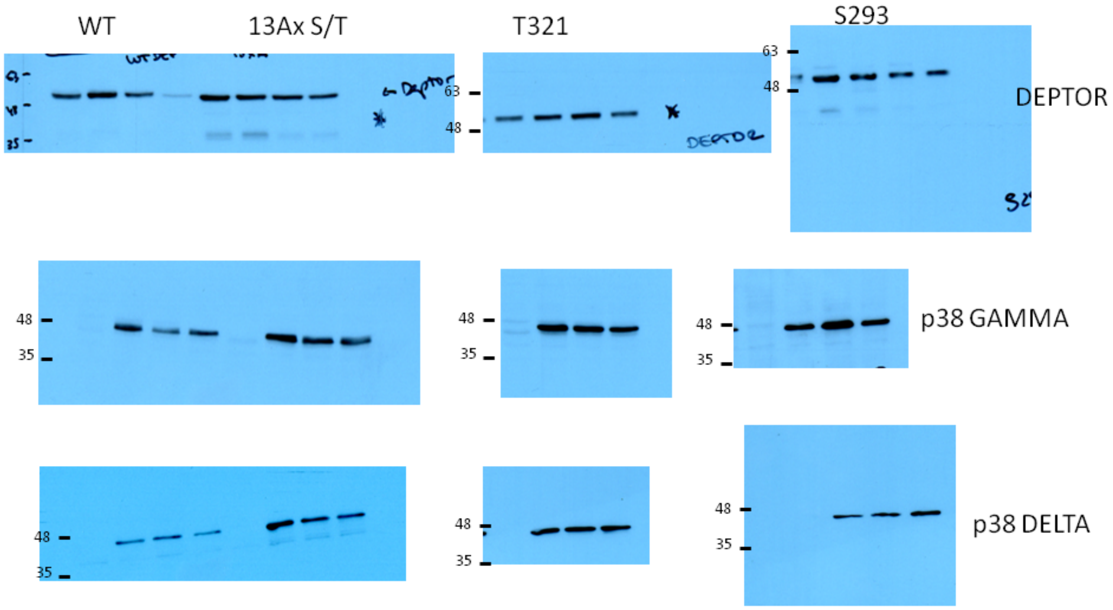

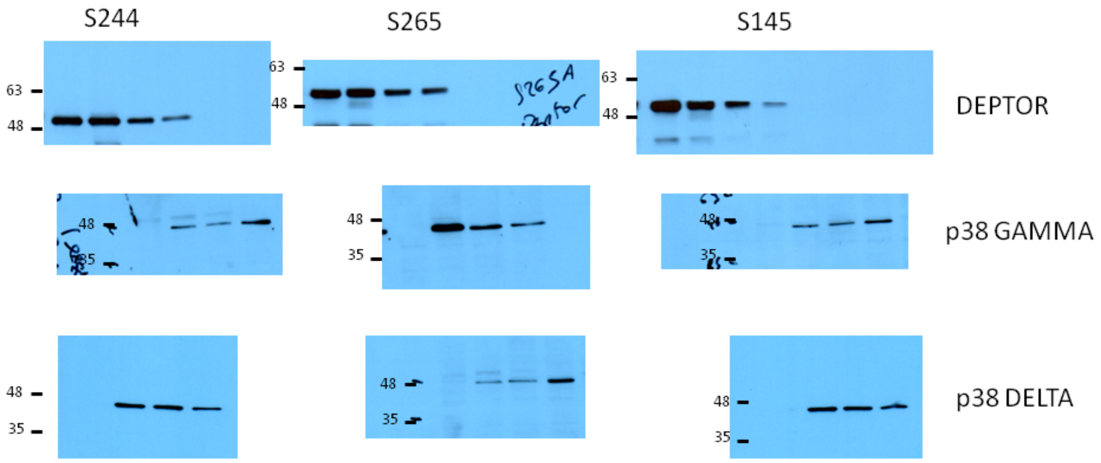

Figure 6A

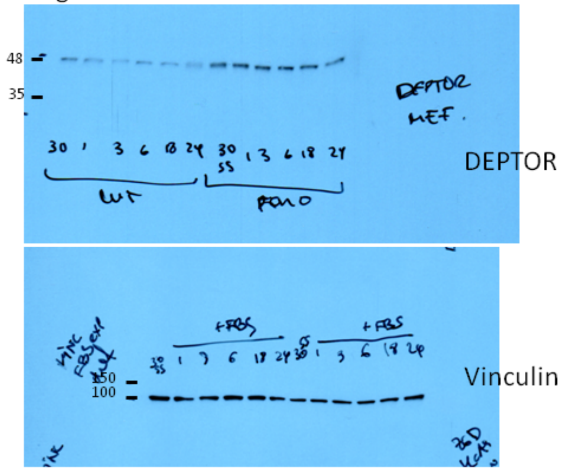

Figure 6D

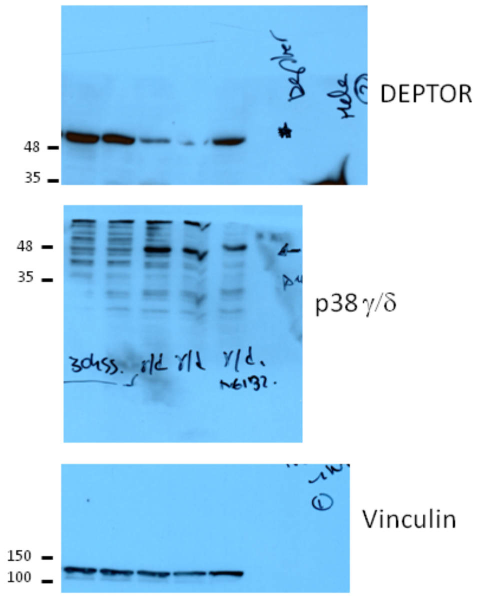

Figure 6E

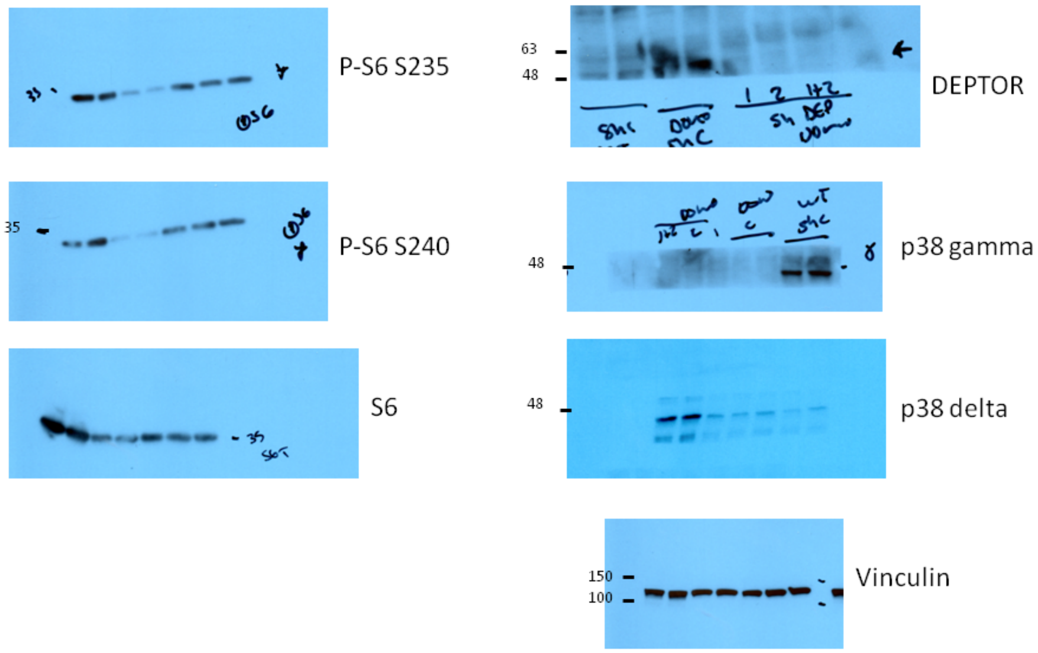

Figure 7A

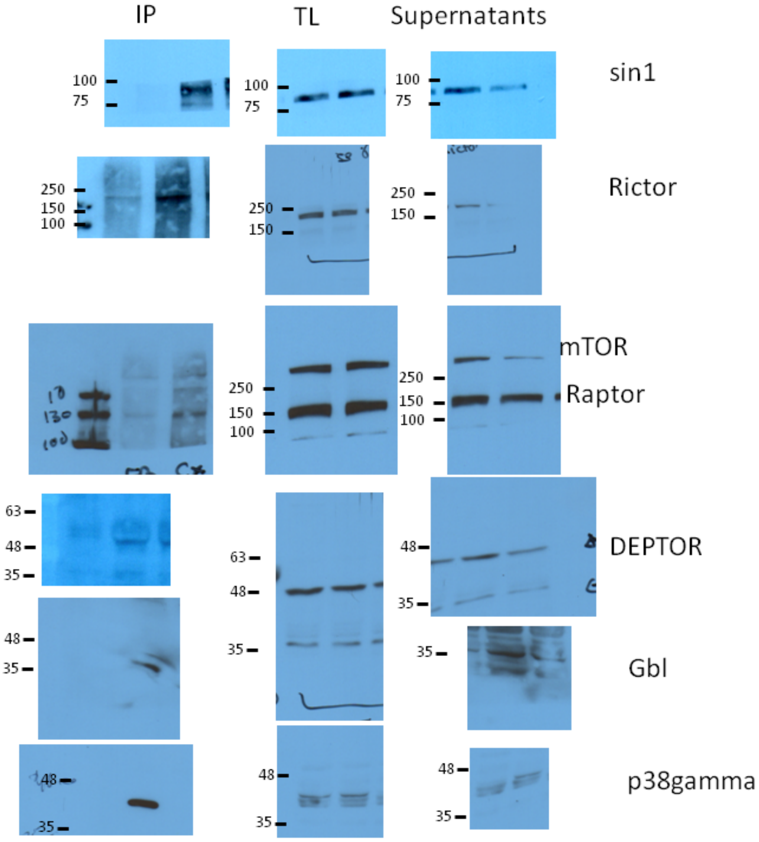

Figure 7b

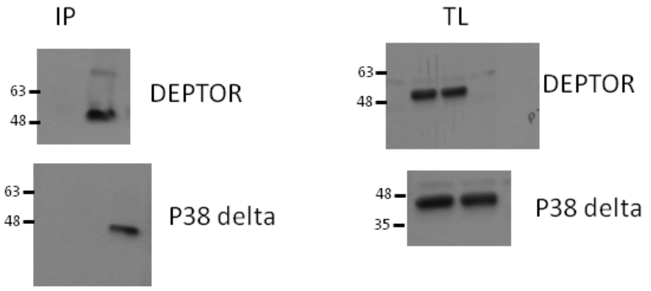

Figure 7d

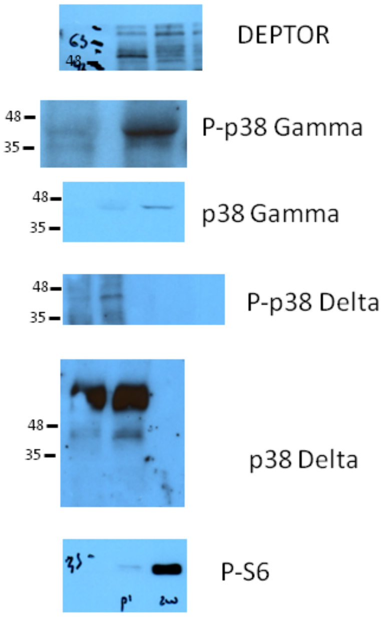

Figure 7c

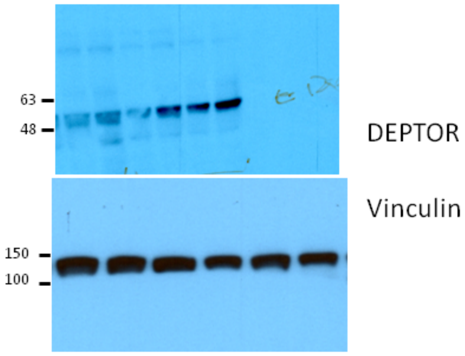

Figure 7e

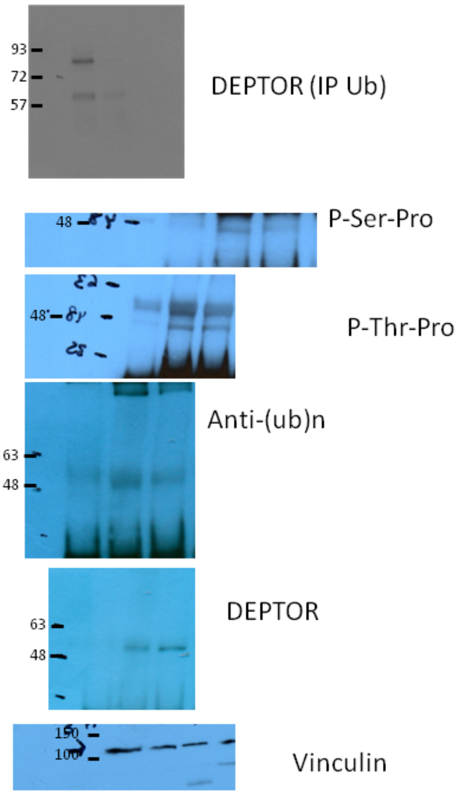

Figure 7f

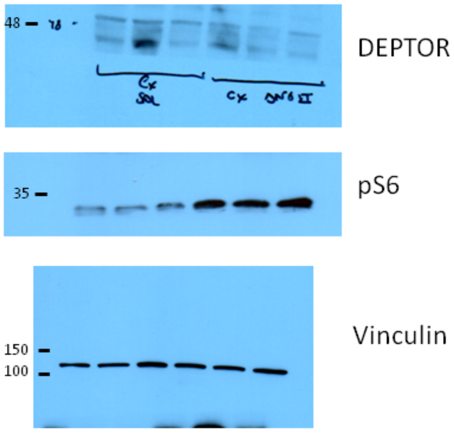

Figure 7j

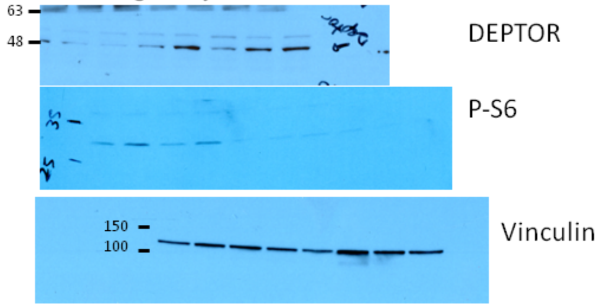

Figure 8A

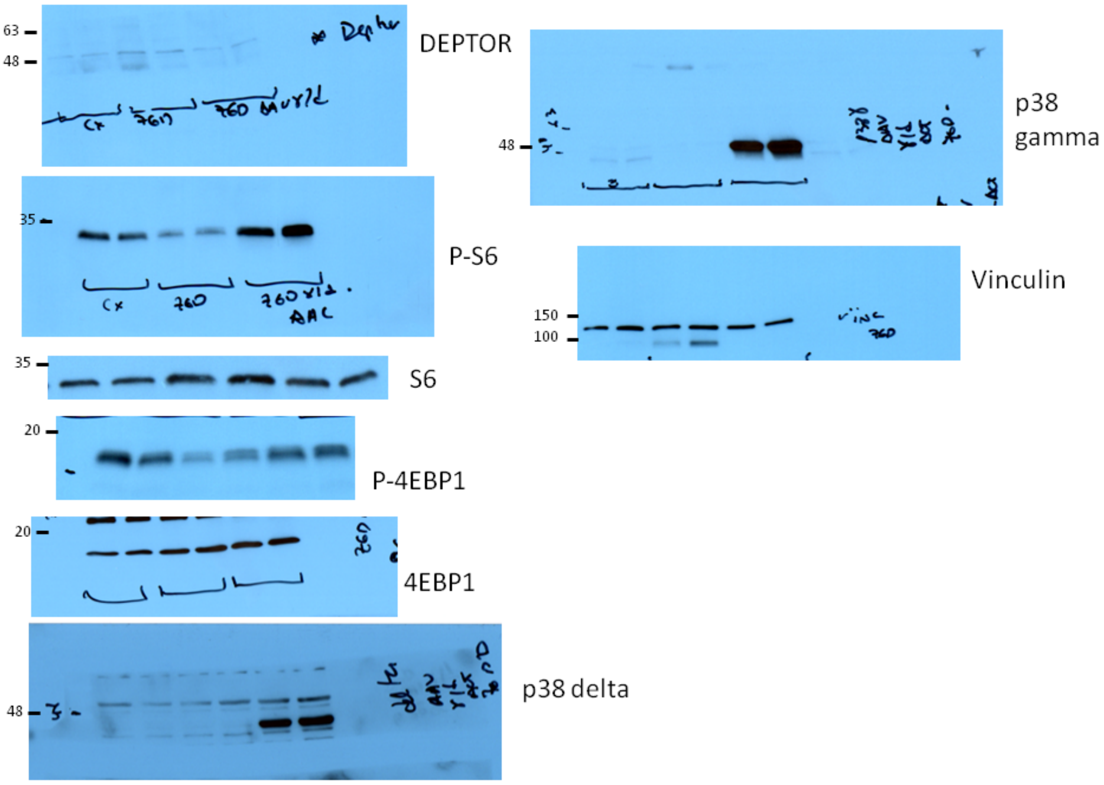

Figure 9A

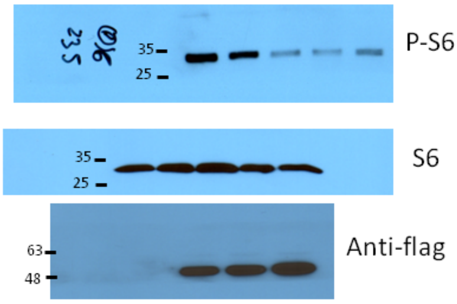

Figure 9E

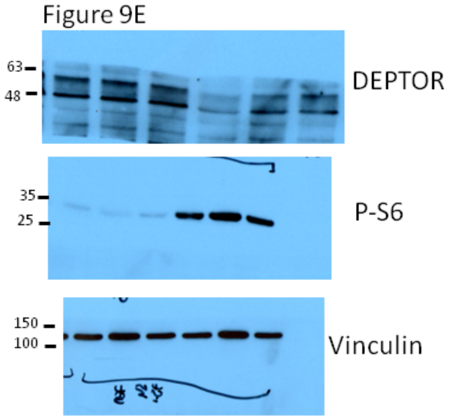

Figure S1 A

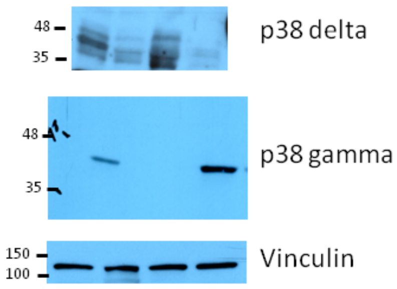

Figure S3A

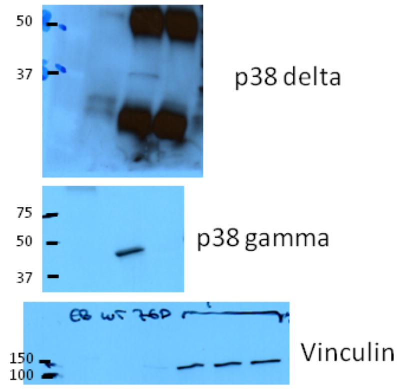

Figure S3B

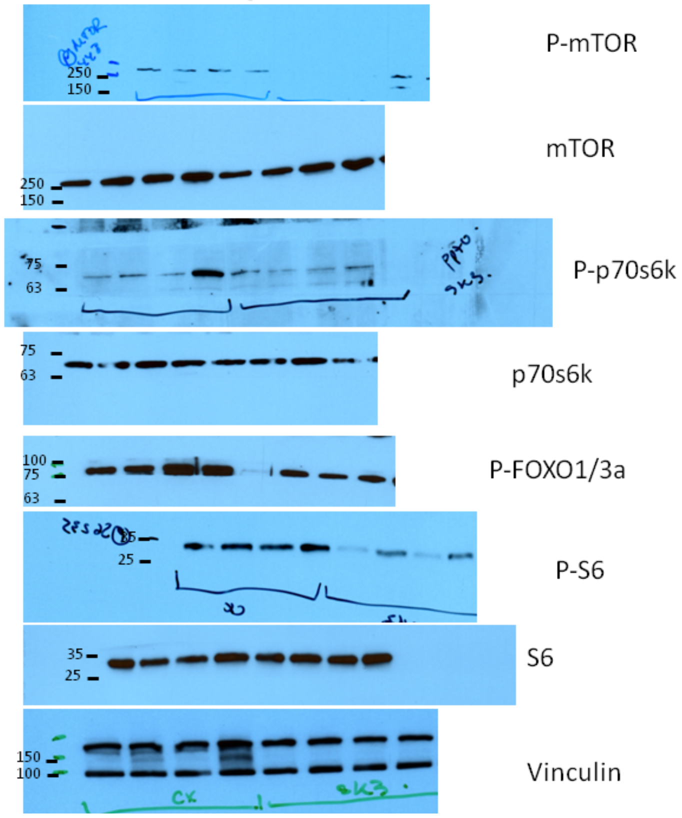

Figure S3C

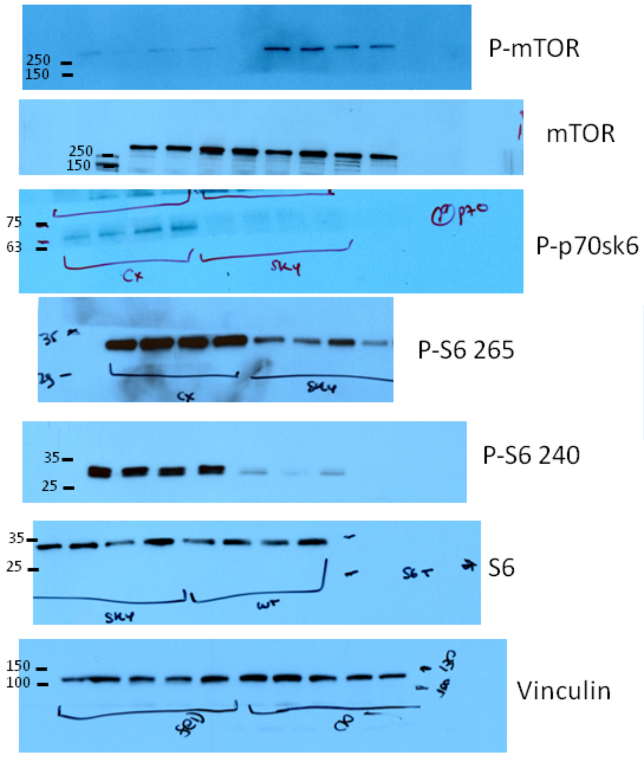

Figure S3D

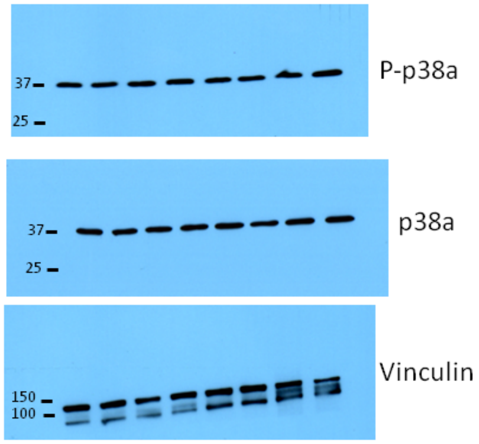

Figure S4A

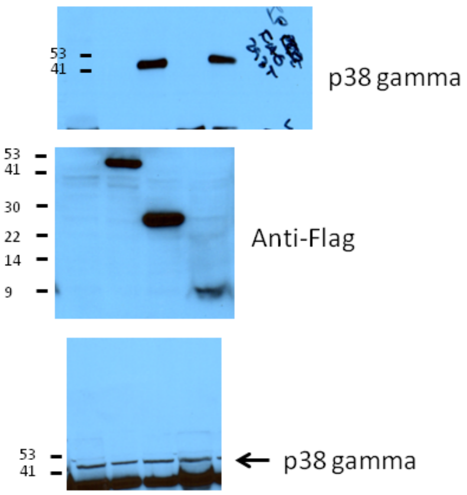

Figure S4B

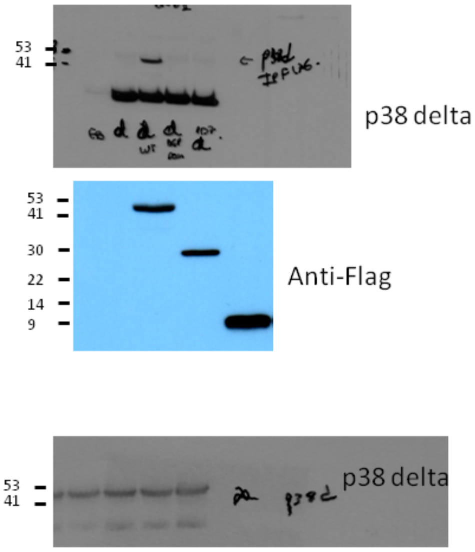

Figure S4C

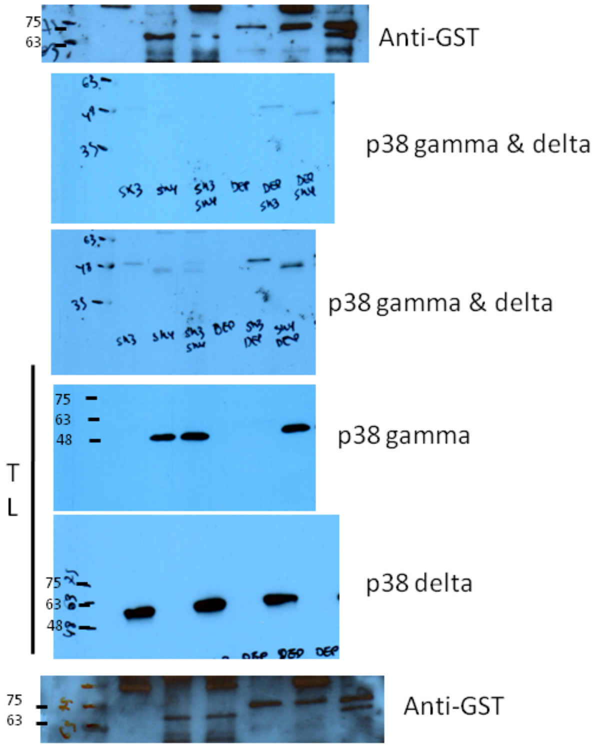

Figure S6A

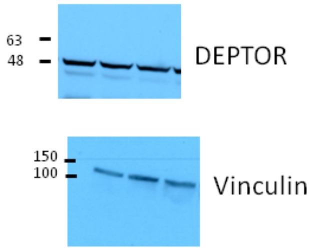

Figure S6B

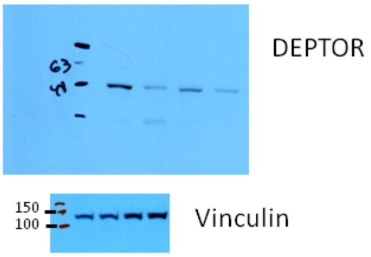

Figure S6C

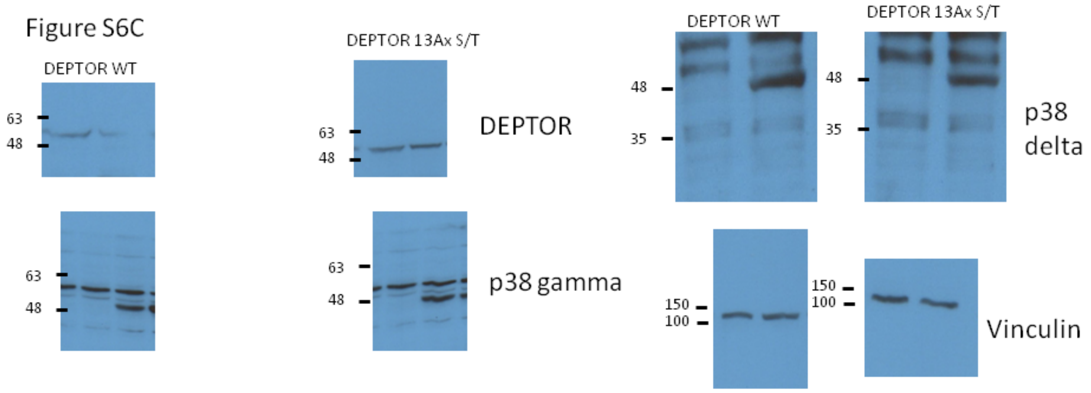

Figure S6E

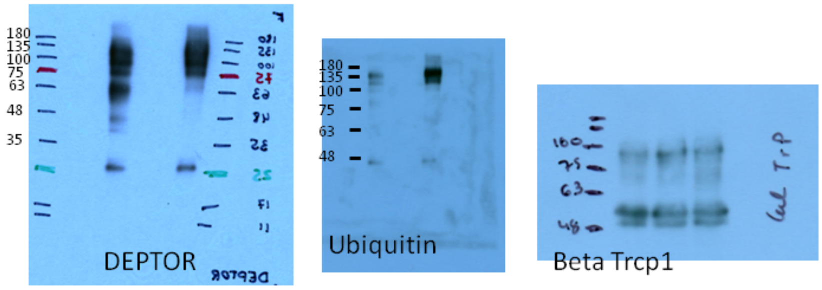

Figure S6F

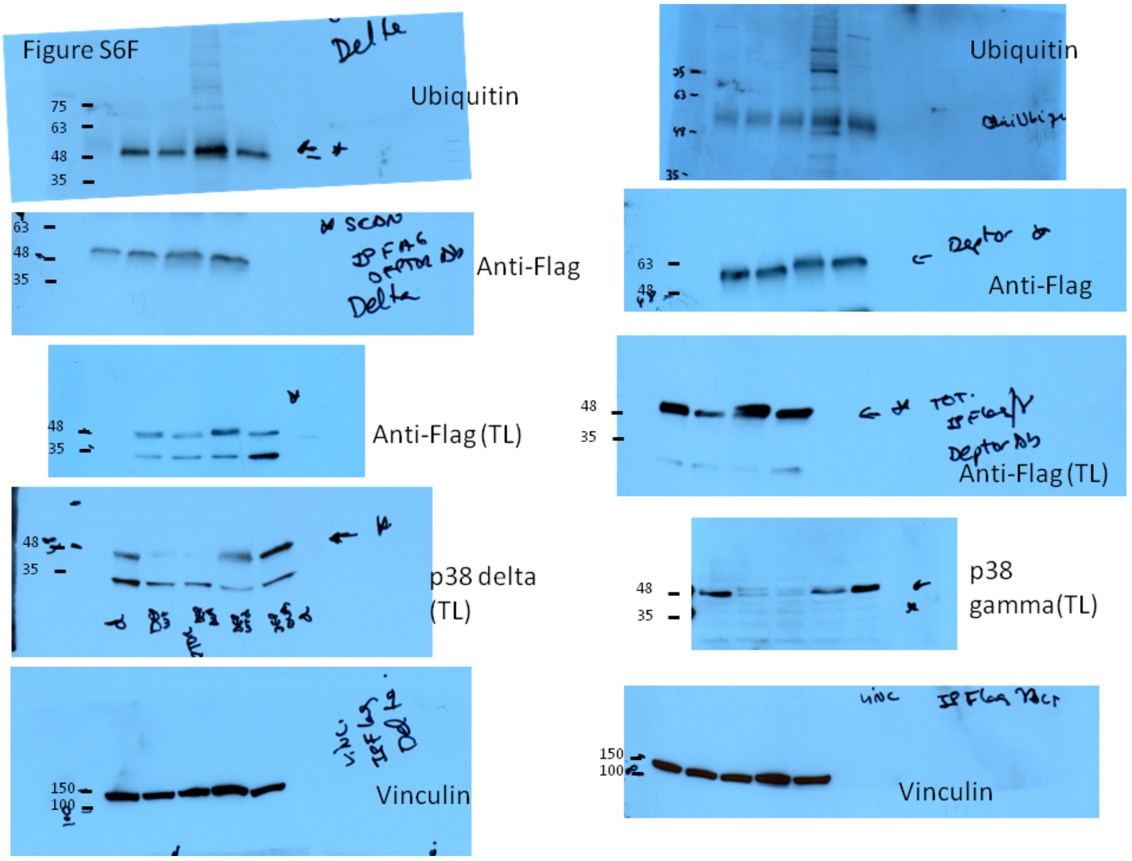

Figure S7A

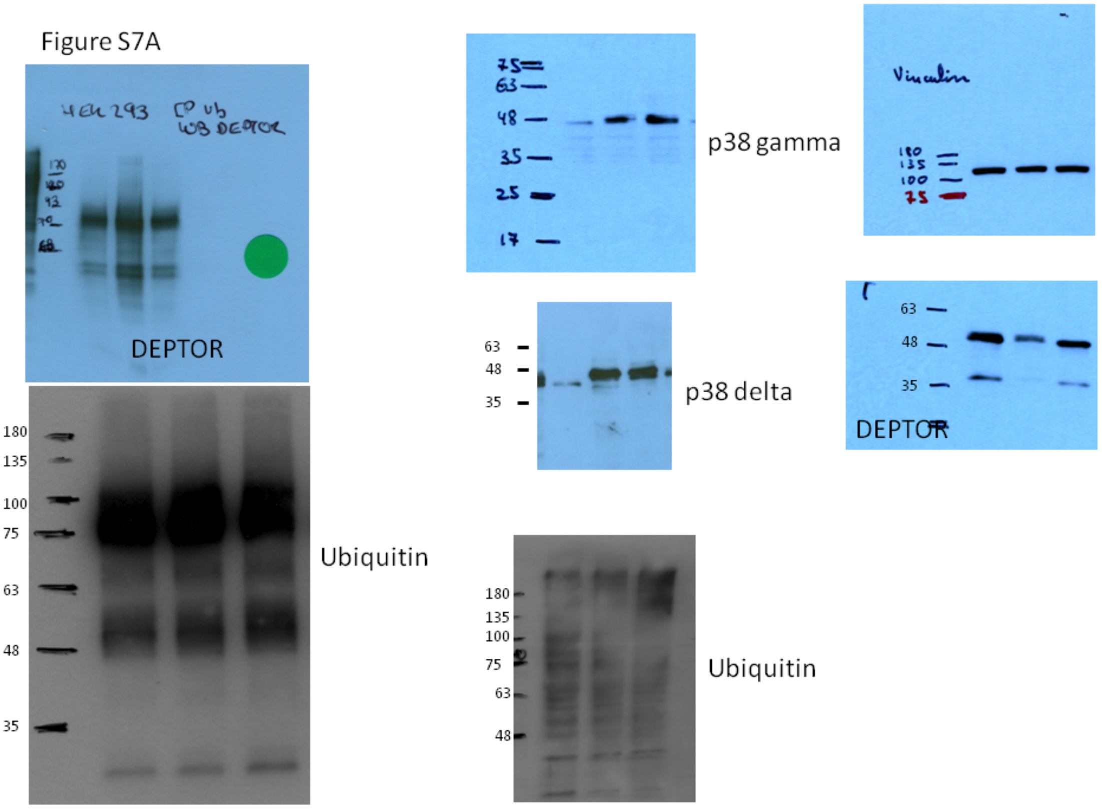

Figure S7B

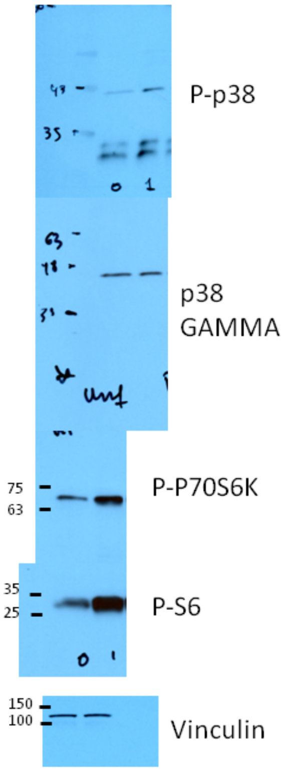

Figure S7C

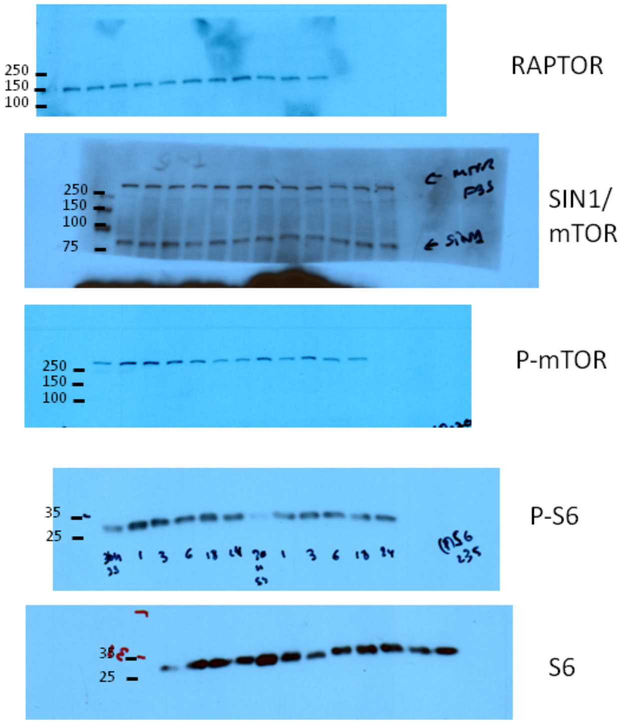

Figure S7D

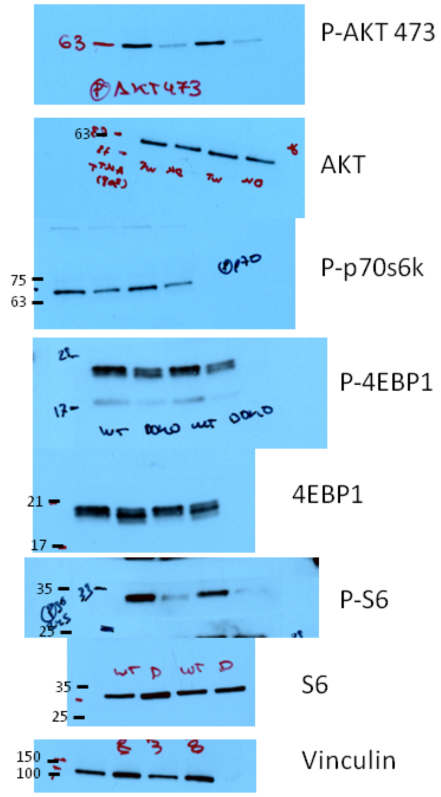

Figure S7E

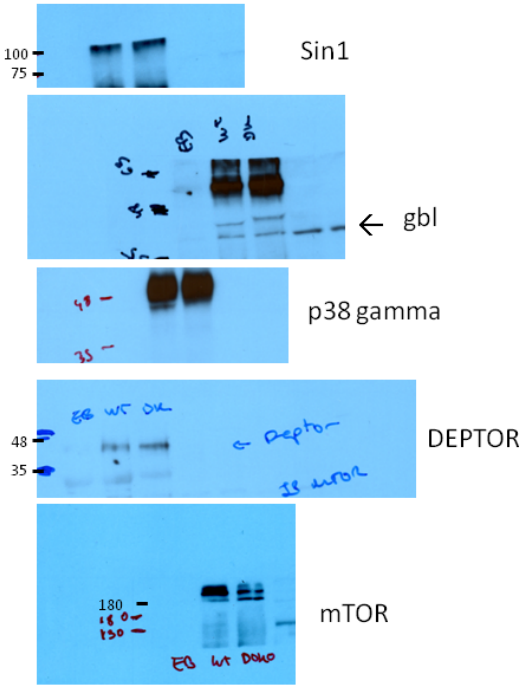

Figure S7F

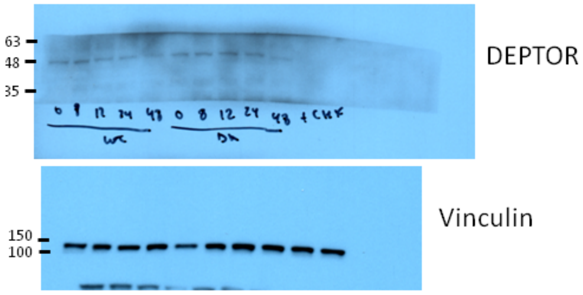

Figure S8A

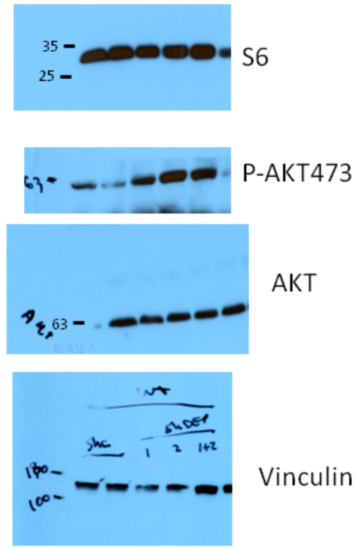

Figure S8A

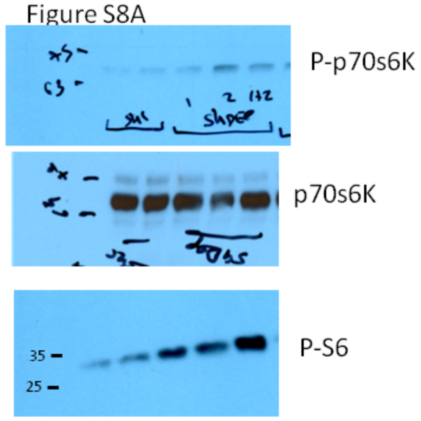

Figure S9A

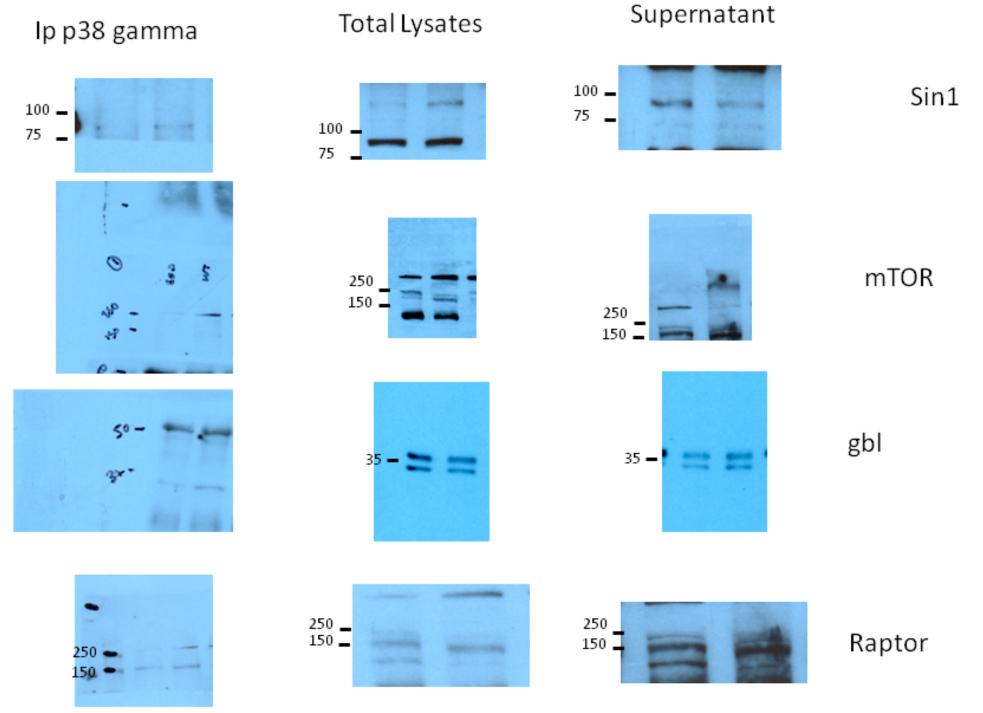

Figure S9A

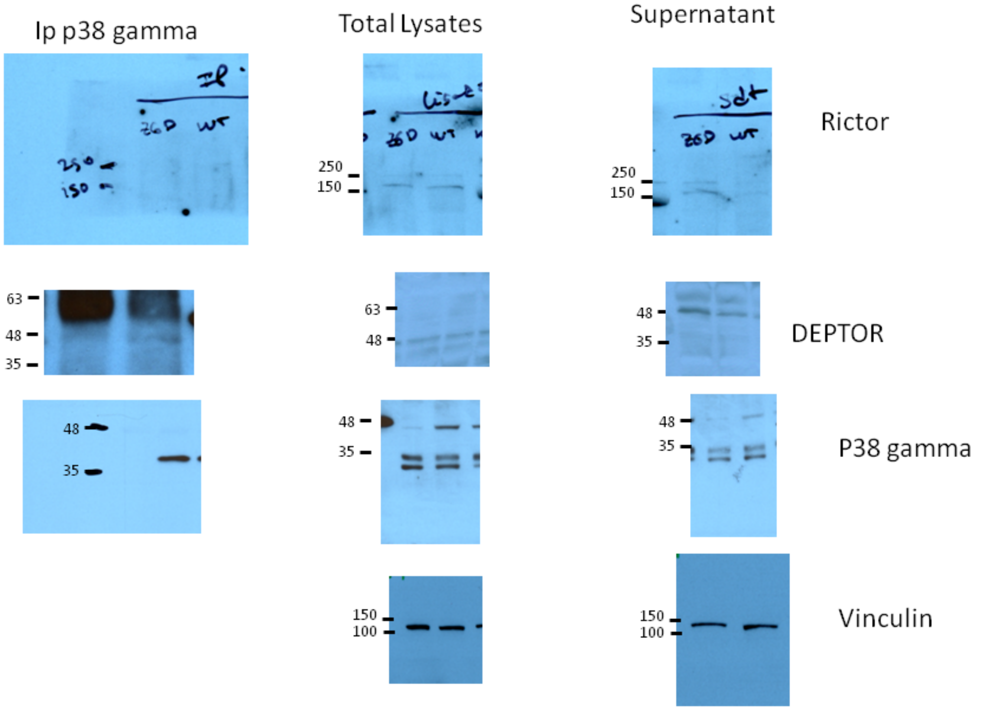

Fig S9 B

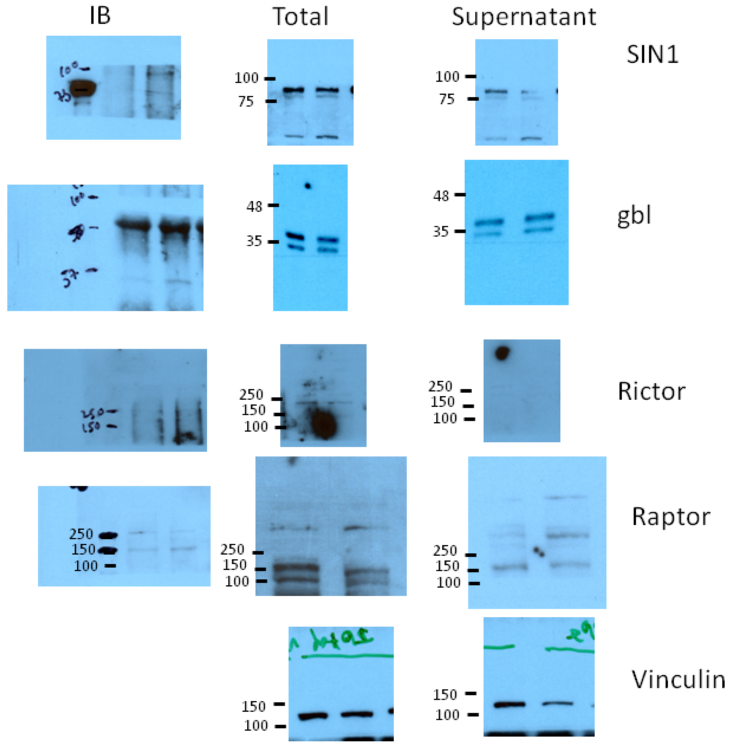

Fig S9 C

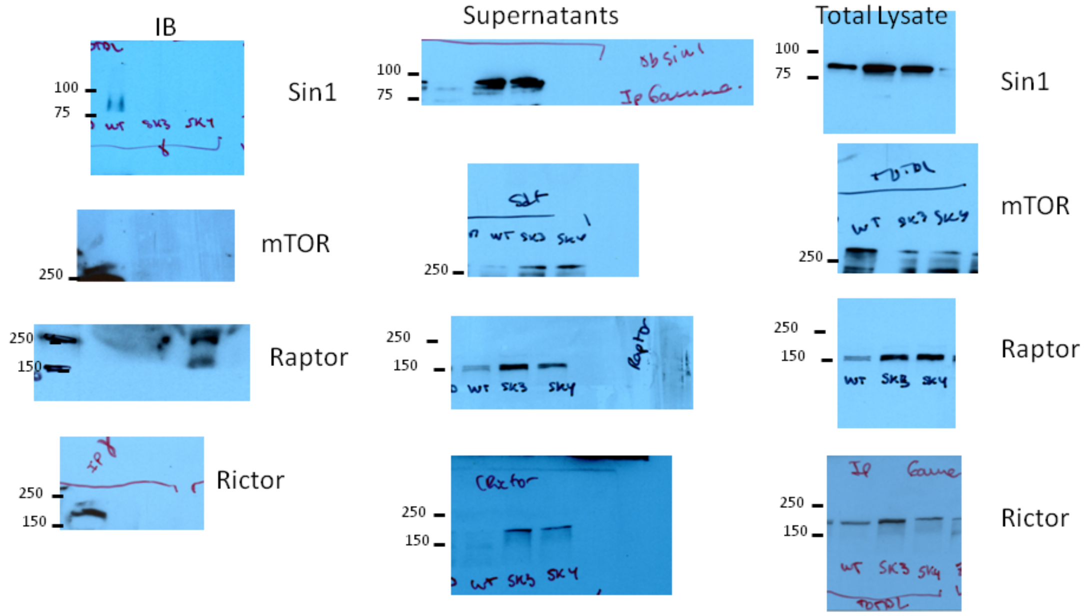

Fig S9 C

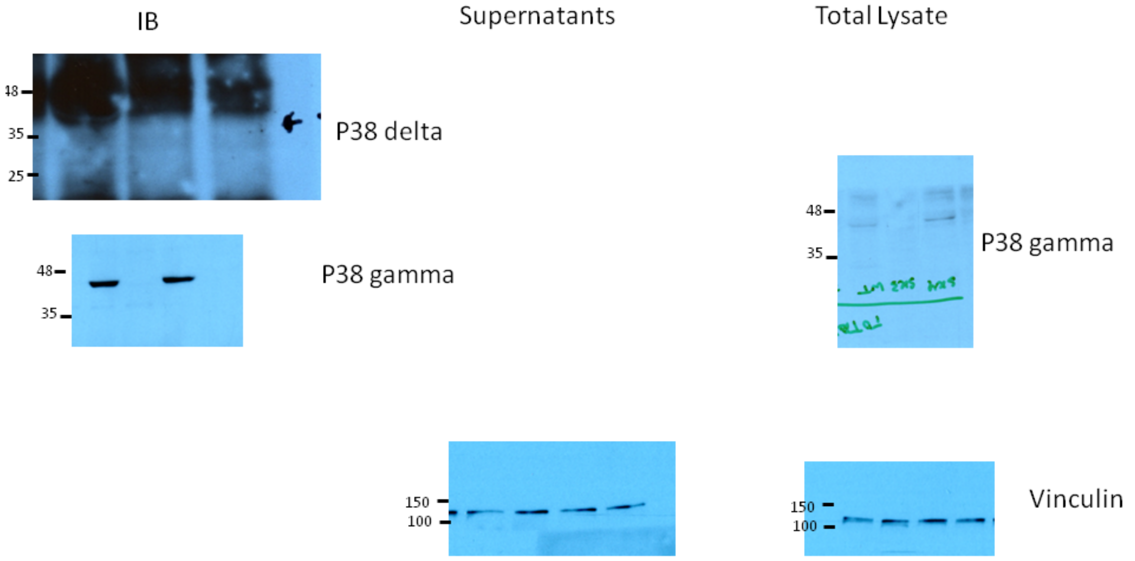

Fig S9 D

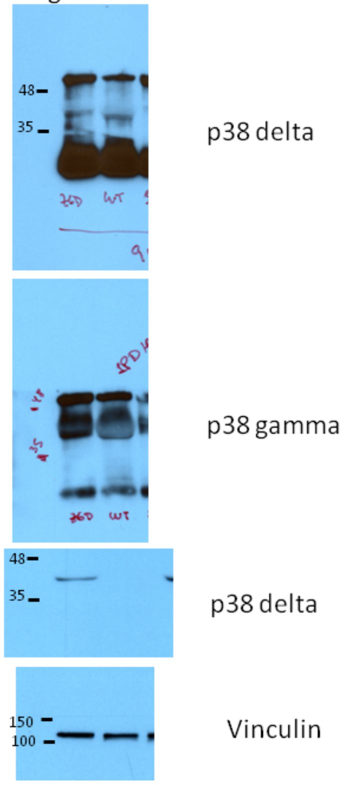

Fig S10 B

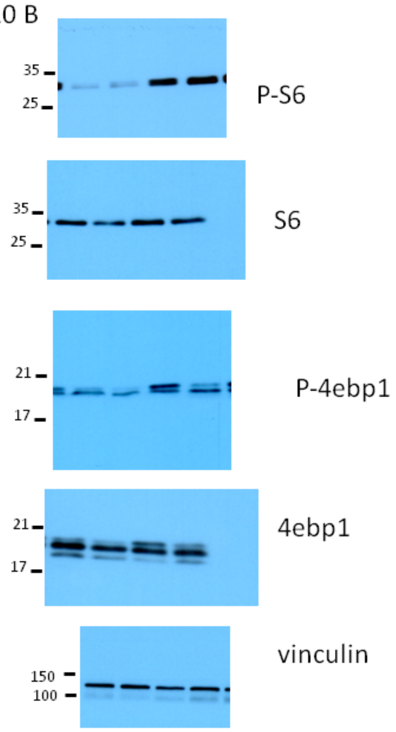

m/z (mi)	MH+2(mi)	MH+3(mi)	Mods	Start	End	Miss	Sequence	length
1494.7022	747.8547	498.9056	1Oxidation 1Phospho	143	154	0	(K)LMSPENTLLQPR(E)	12
1398.741	699.8741	466.9185		143	154	0	(K)LMSPENTLLQPR(E)	12
1414.7359	707.8716	472.2501	1Oxidation	143	154	0	(K)LMSPENTLLQPR(E)	12
1478.7073	739.8573	493.5739	1Phospho	143	154	0	(K)LMSPENTLLQPR(E)	12
2070.0536	1035.5304	690.6894		143	160	1	(K)LMSPENTLLQPREEEGVK(Y)	18
2086.0485	1043.5279	696.021	1Oxidation	143	160	1	(K)LMSPENTLLQPREEEGVK(Y)	18
2166.0148	1083.5111	722.6765	1Oxidation 1Phospho	143	160	1	(K)LMSPENTLLQPREEEGVK(Y)	18
2150.0199	1075.5136	717.3448	1Phospho	143	160	1	(K)LMSPENTLLQPREEEGVK(Y)	18
1936.9528	968.98	646.3225	1Phospho	312	328	0	(K)RPVTSEELLTPGAPYAR(K)	17
1856.9865	928.9969	619.667		312	328	0	(K)RPVTSEELLTPGAPYAR(K)	17
2065.0478	1033.0275	689.0208	1Phospho	312	329	1	(K)RPVTSEELLTPGAPYARK(T)	18
1985.0815	993.0444	662.3653		312	329	1	(K)RPVTSEELLTPGAPYARK(T)	18
1157.5507	579.279	386.5218		258	268	0	(K)STSFMSVSPSK(E)	11
1253.5119	627.2596	418.5088	1Oxidation 1Phospho	258	268	0	(K)STSFMSVSPSK(E)	11
1173.5456	587.2764	391.8534	1Oxidation	258	268	0	(K)STSFMSVSPSK(E)	11
1237.517	619.2622	413.1772	1Phospho	258	268	0	(K)STSFMSVSPSK(E)	11
1527.7723	764.3898	509.929		258	271	1	(K)STSFMSVSPSKEIK(I)	14
1623.7336	812.3704	541.916	1Oxidation 1Phospho	258	271	1	(K)STSFMSVSPSKEIK(I)	14
1543.7672	772.3873	515.2606	1Oxidation	258	271	1	(K)STSFMSVSPSKEIK(I)	14
1607.7387	804.373	536.5844	1Phospho	258	271	1	(K)STSFMSVSPSKEIK(I)	14
2364.2323	1182.6198	788.7489	1Phospho	308	328	1	(K)SVLKRPVTSEELLTPGAPYAR(K)	21
2284.266	1142.6366	762.0935		308	328	1	(K)SVLKRPVTSEELLTPGAPYAR(K)	21
1285.6457	643.3265	429.2201		257	268	1	(R)KSTSFMSVSPSK(E)	12
1381.6069	691.3071	461.2072	1Oxidation 1Phospho	257	268	1	(R)KSTSFMSVSPSK(E)	12
1301.6406	651.3239	434.5517	1Oxidation	257	268	1	(R)KSTSFMSVSPSK(E)	12
1365.612	683.3096	455.8755	1Phospho	257	268	1	(R)KSTSFMSVSPSK(E)	12
2011.9922	1006.4998	671.3356	1Phospho	139	154	1	(R)LYEKLMSPENTLLQPR(E)	16
2027.9872	1014.4972	676.6672	1Oxidation 1Phospho	139	154	1	(R)LYEKLMSPENTLLQPR(E)	16
1932.0259	966.5166	644.6802		139	154	1	(R)LYEKLMSPENTLLQPR(E)	16
1948.0208	974.5141	650.0118	1Oxidation	139	154	1	(R)LYEKLMSPENTLLQPR(E)	16

Supplementary table 1. Peptides selected in this study for the in vitro and in vivo analysis of p38γ and p38δ phosphorylation of human DEPTOR by PRM

Monoisotopic masses for singly (m/z), doubly (MH+2) or triply (MH+3) charged ions from trypsin-digested peptides of human DEPTOR (Uniprot entry Q8TB45 - DPTOR_HUMAN), containing SP or TP consensus phosphorylation motifs of p38γ and p38δ, and their unmodified or Met-oxidized counterparts. In red, selected masses for each sequence. Parent and fragment masses for later analyses were generated using the ProteinProspector Tools (http://prospector.ucsf.edu/prospector/mshome.htm).

Gene	Primer	Sequence
Deptor	Fw	ATAGACGGCACCATCTCAA
	Rev	GTCGGCTAATTTCTGCATG
S145A mutation	Fw	GGCTATATGAAAAGCTGATG**GC**CCCTGAAAACACACTCCTGC
	Rev	GCAGGAGTGTGTTTTCAGGGGCCATCAGCTTTTCATATAGCC
S244A mutation	Fw	CCAGGAAACTCATGAC**GC**TCCCTTCTGCCTGAGG
	Rev	CCTCAGGCAGAAGGGAGCGTCATGAGTTTCCTGG
S265A mutation	Fw	CCAGCTTTATGTCAGTG**GC**CCCCAGCAAGGAGATC
	Rev	GATCTCCTTGCTGGGGGCCACTGACATAAAGCTGG
S293A mutation	Fw	GCTACTTCAGCAGC**GC**CCCCACCCTCAGC
	Rev	GCTGAGGGTGGGGGCGCTGCTGAAGTAGC
T321A mutation	Fw	**CC**TCTGAGGAACTCCTT**G**CTCCCGGGGCTCCG
	Rev	CGGAGCCCCGGGAGCAAGGAGTTCCTCAGAGG

Supplementary Table 2. Primers sequences.

SUPPLEMENTARY METHODS

Detection of DEPTOR Phosphorylation Sites *In Vivo*

HEK-293T cells were transfected with FLAG-DEPTOR using the calcium phosphate method. At 30 hr post-transfection, 293T cells were treated with 10 µM MG132 for 16 hr to block the 26S proteasome pathway prior to collecting the whole cell lysates for FLAG-immunoprecipitation. After extensive washing with PBS 1x buffer, 10µl of sample buffer was added to FLAG-immunoprecipitates, which were boiled for 5 minutes at 95ºC. The samples were reduced, alkylated, digested with trypsin, and desalted on C18 reverse phase microcolumns as before [1,2]. Analyses were performed on a nano HPLC system coupled with a hybrid quadrupole orbitrap mass spectrometer (QExactive, Thermo Scientific). For LC analysis, each sample was loaded in Buffer A (0.1% formic acid) and on-line desalted on a 2 cm packed pre-column (Thermo Acclaim

PepMap 100). Analytical separation was performed over a 50 cm column (Thermo Acclaim PepMap 100, 75 µm ID x 500 mm) at 200 nL/min with a 180 min gradient from 8 to 31% of Buffer B (0.1% formic acid / 90 % acetonitrile) using an EASY-nLC 1000 HPLC (Thermo Scientific). Samples were analysed randomly with an extensive column wash between each sample to minimize carry-over. Phosphorylated peptides detected in the *in vitro* experiments, and their non-phosphorylated counterparts, were monitored using the parallel reaction monitoring (PRM) mode. In all experiments, a full mass spectrum with 2 microscans at 140.000 resolution relative to m/z 200 (AGC target 1×10^6, 20 ms maximum injection time, m/z 400–1200) was followed by PRM scans at 17.500 resolution (AGC target 2×10^5, 120 ms maximum injection time, isolation width of ±2 Th) and triggered by a scheduled inclusion list of the selected peptide ions (Supplementary Table 1). Fragmentation was performed in the HCD collision cell using anormalized collision energy of 27%. Quantification was carried out post-acquisition using the extracted ion chromatograms (XICs) using a ±3 ppm mass tolerance, unless otherwise noted. Detection of the targeted peptide was based on the presence of the intact monoisotopic precursor signal, mass error within ±3 ppm, and manual verification of the MS/MS spectra. Proteome Discoverer 1.4.0.288 (Thermo Scientific) was also used together with Scaffold (version Scaffold_4.4.1, Proteome Software Inc., Portland, OR) to validate MS/MS based peptide identifications and fragment assignments.

Site-Directed Mutagenesis

The DEPTOR cDNA in pRK5 (plasmid #21334, Addgene) was mutagenized with the QuikChange II XL Site-Directed Mutagenesis Kit (Agilent Technologies). The primers used are specified in the Supplementary table 2.

qRT-PCR

The expression of mRNA was examined by qRT-PCR using a 7900 Fast Real Time thermocycler and FAST SYBR GREEN assays (Applied Biosystems). Relative mRNA expression was normalized to *Gapdh* mRNA measured in each sample. *Deptor* was amplified using the primers shown in Supplemental Table 2.

In vitro ubiquitin assay

Cullin-βTrCP E3 complex was precipitated from 293 cells overexpressing both proteins with myc. GST-DEPTOR protein (H00064798, Novus biologicals) was incubated ofor 30 minutes with 1 μg of active recombinant p38g and p38d or without kinases (provided by MRC Protein Phosphorylation and Ubiquitylation Unit, Dundee, UK) in the presence of 200 μM cold ATP followed by incubation with Cullin-βTrCP E3 complex in the presence of E1 and E2 in a ubiquitin reaction buffer (Abcam). Polyubiquitinated DEPTOR was resolved by SDS-PAGE and detected by IB with anti-DEPTOR Ab and antiubiquitin.

SUPPLEMENTARY REFERENCES

1 Inuzuka, H. *et al.* Phosphorylation by casein kinase I promotes the turnover of the Mdm2 oncoprotein via the SCF(beta-TRCP) ubiquitin ligase. *Cancer Cell* **18**, 147-159 (2010).

2 Dibble, C. C., Asara, J. M. & Manning, B. D. Characterization of Rictor phosphorylation sites reveals direct regulation of mTOR complex 2 by S6K1. *Mol Cell Biol* **29**, 5657-5670 (2009).
